# Supplementary figures and images for: Adipocyte microRNA-802 promotes adipose tissue inflammation and insulin resistance by modulating macrophages in obesity (part 1 of 2)
Source: eLife. 2024 Nov 26;13:e99162. doi: 10.7554/eLife.99162 (PMC11651656; doi:10.7554/eLife.99162)

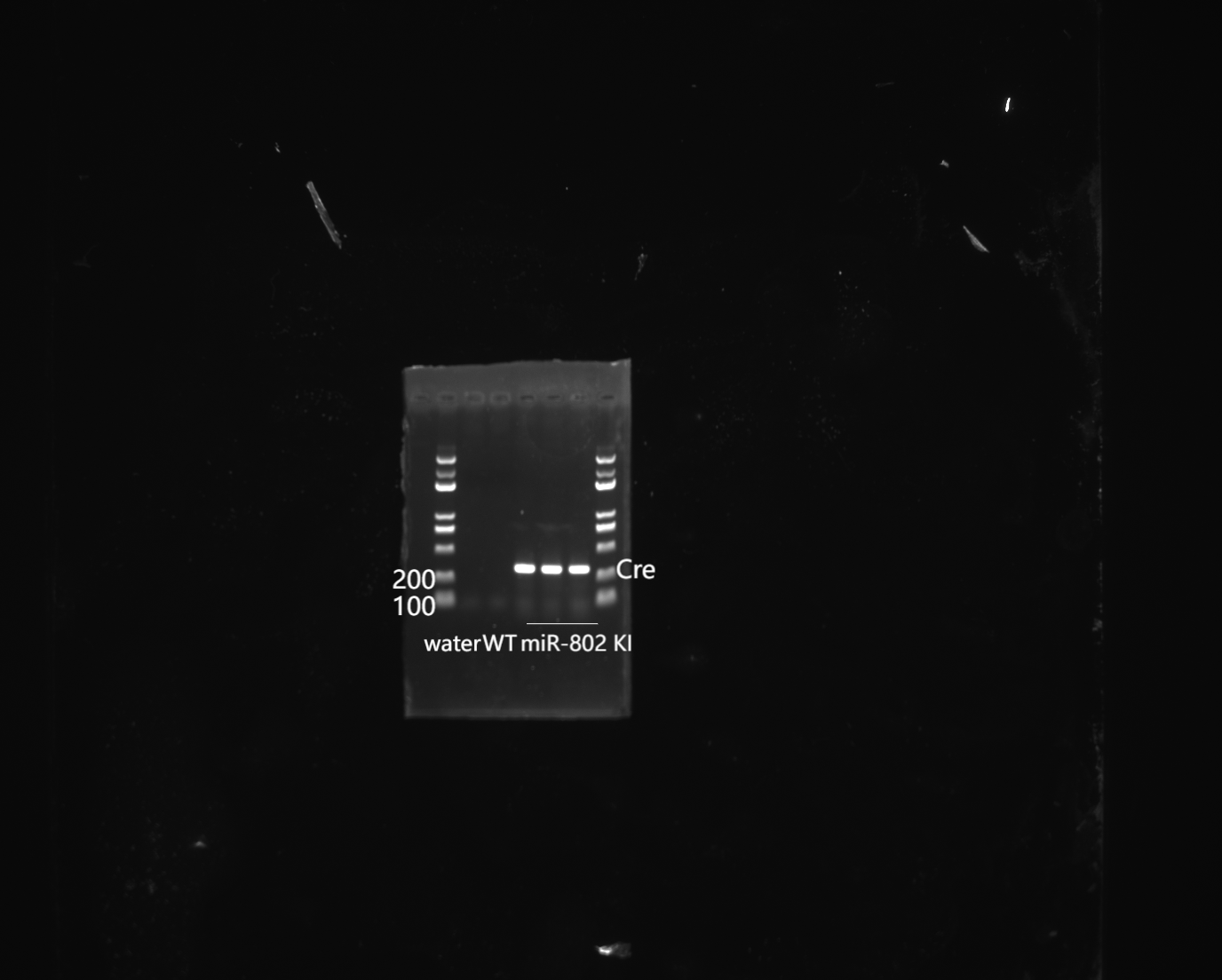

Supplement: Figure 2—figure supplement 1—source data 1. — The original files of the full raw unedited gels of Mir802 KI mice. [file elife-99162-fig2-figsupp1-data1.zip › Figure 2-figure supplement 1-source data 1/S2B-Cre.png]

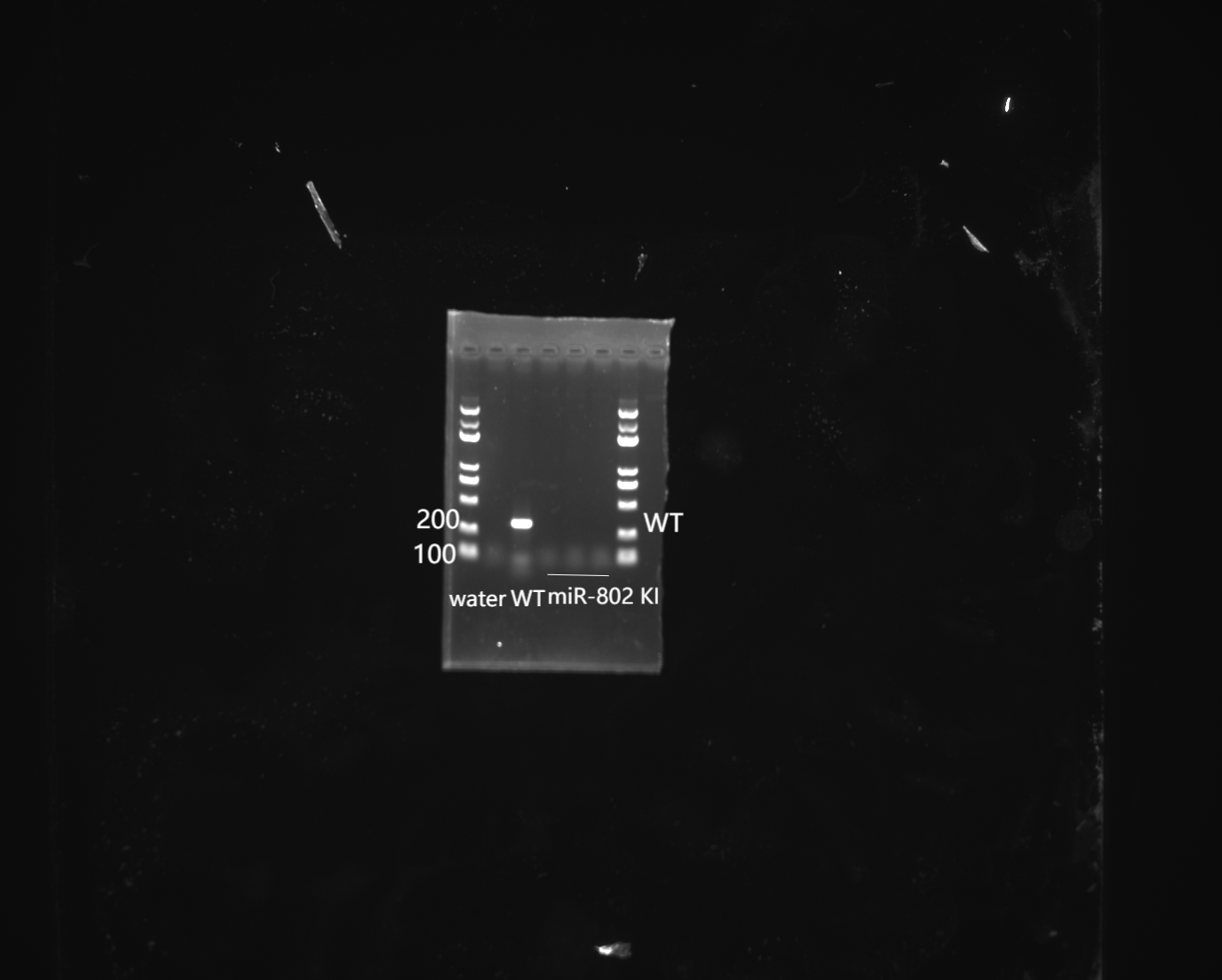

Supplement: Figure 2—figure supplement 1—source data 1. — The original files of the full raw unedited gels of Mir802 KI mice. [file elife-99162-fig2-figsupp1-data1.zip › Figure 2-figure supplement 1-source data 1/S2B-WT.png]

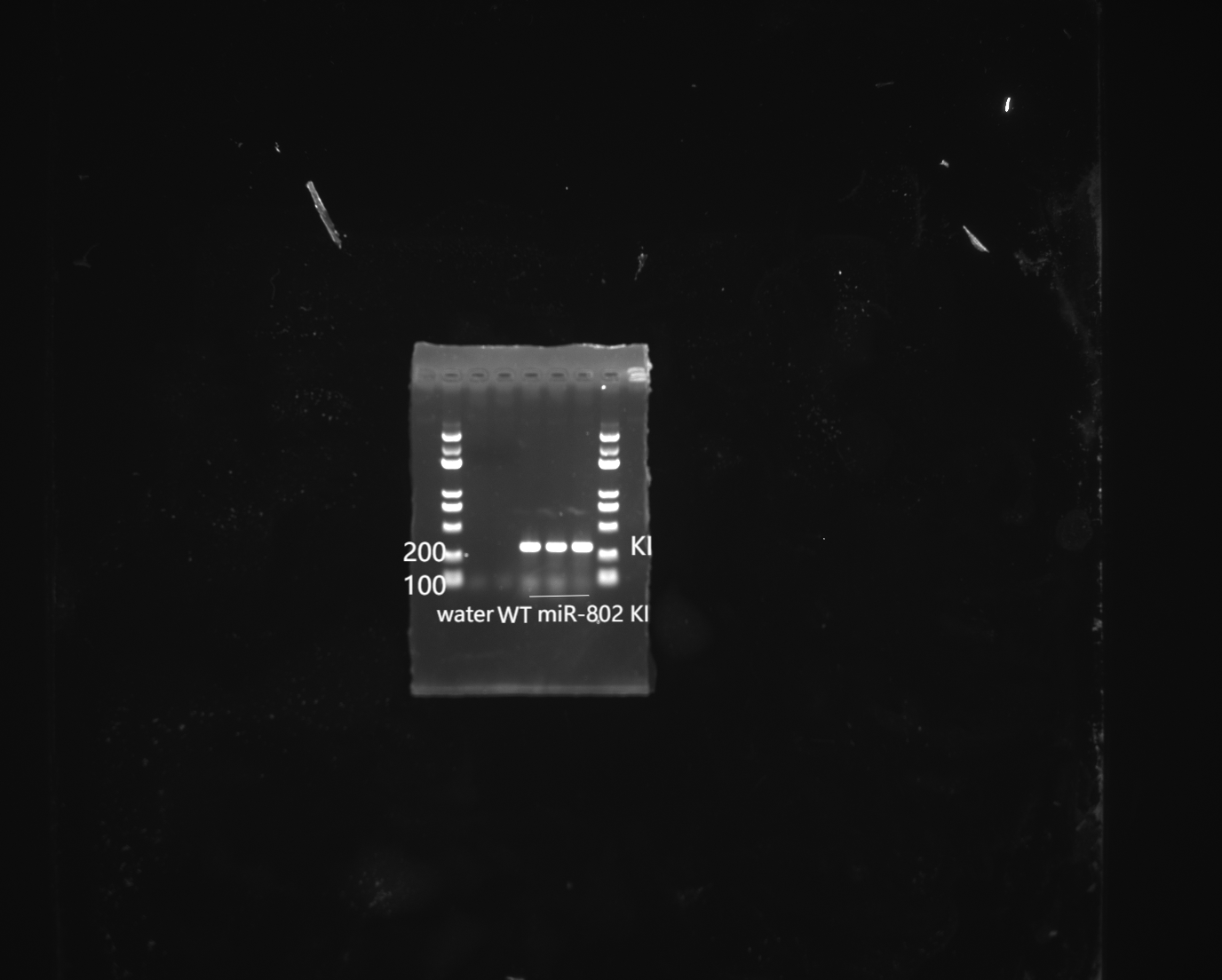

Supplement: Figure 2—figure supplement 1—source data 1. — The original files of the full raw unedited gels of Mir802 KI mice. [file elife-99162-fig2-figsupp1-data1.zip › Figure 2-figure supplement 1-source data 1/S2B-KI.png]

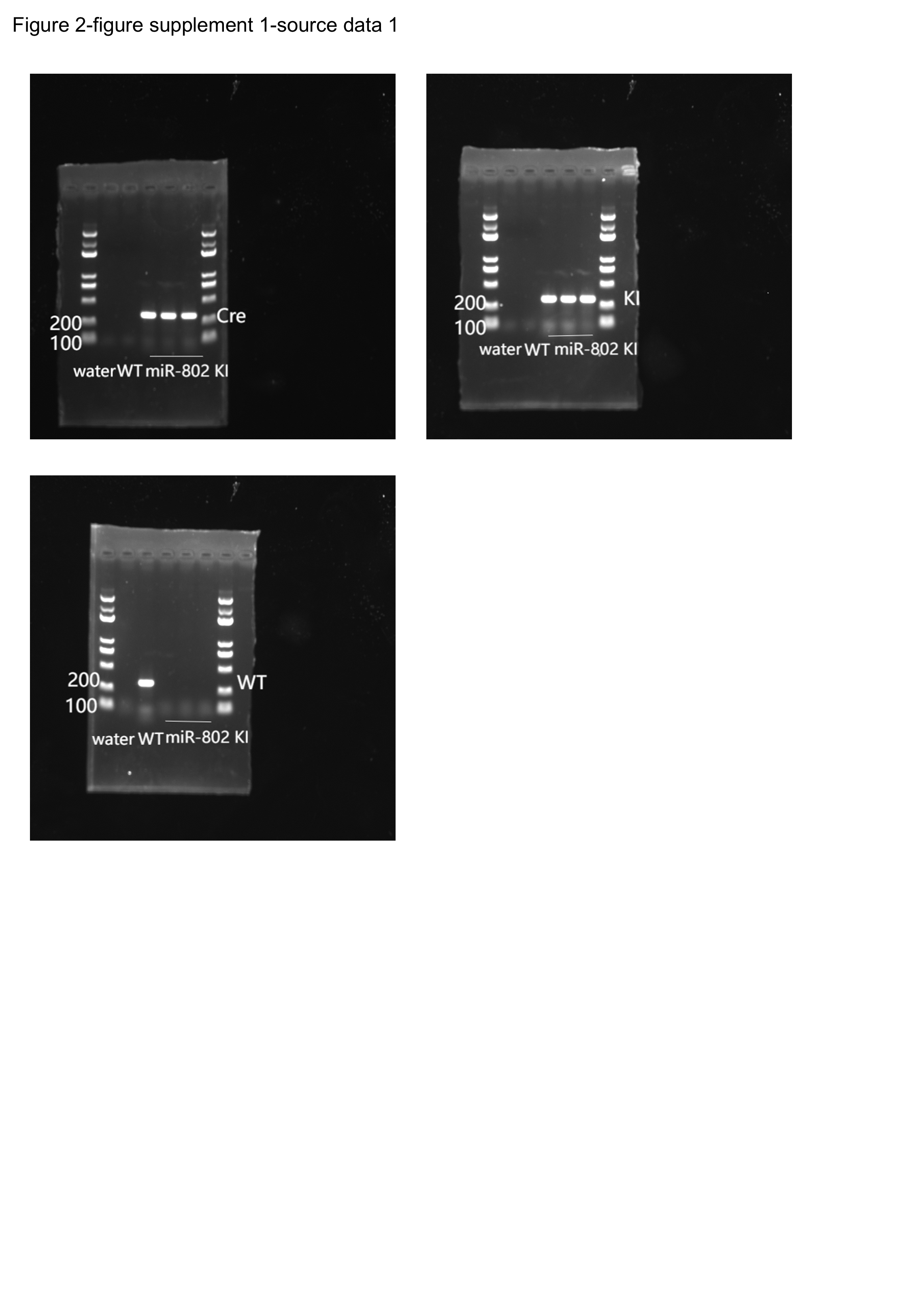

Supplement: Figure 2—figure supplement 1—source data 1. — The original files of the full raw unedited gels of Mir802 KI mice. [file elife-99162-fig2-figsupp1-data1.zip › Figure 2ΓÇöfigure supplement 1-source data 1.tif]

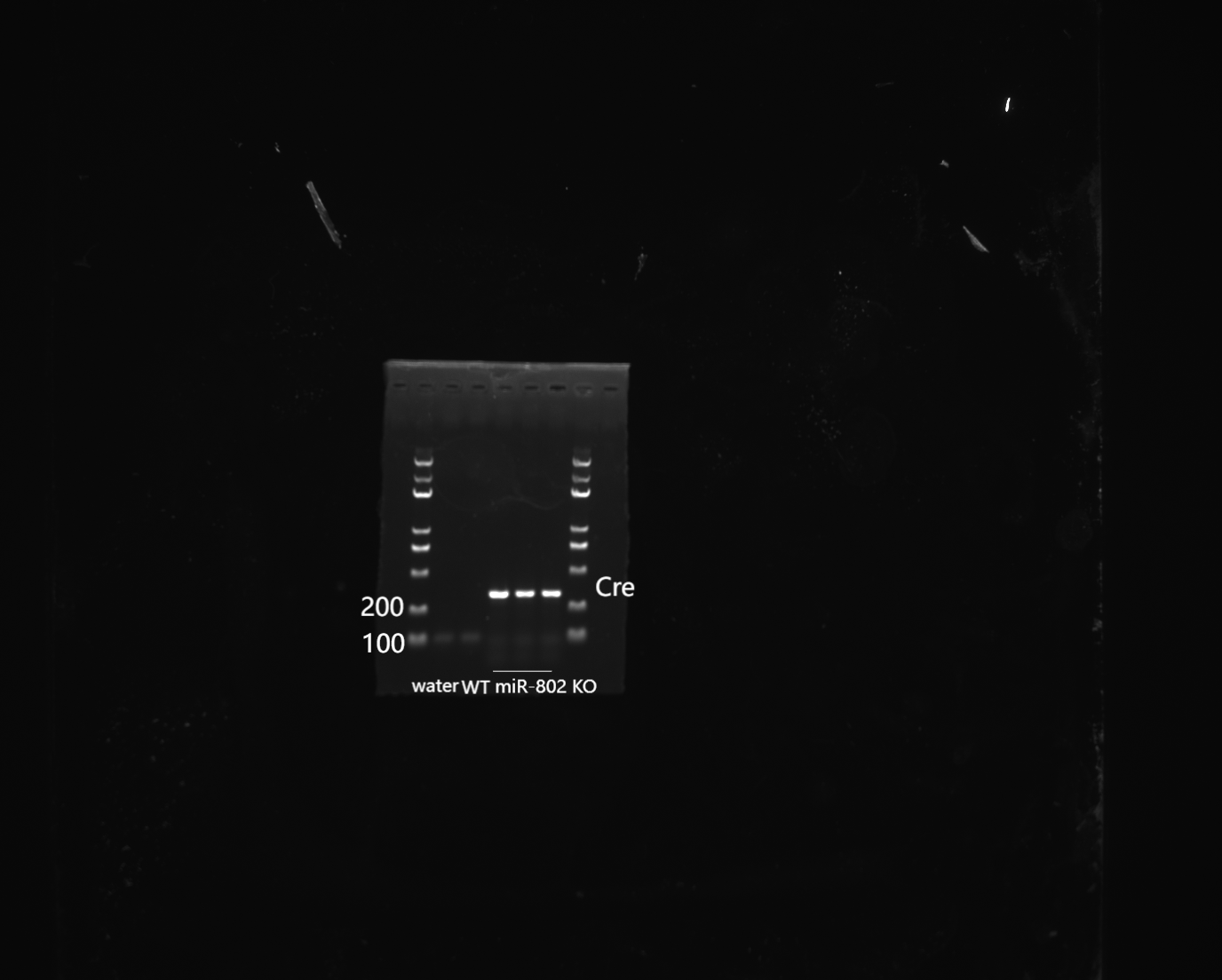

Supplement: Figure 3—figure supplement 1—source data 1. — The original files of the full raw unedited gels of Mir802 KO mice. [file elife-99162-fig3-figsupp1-data1.zip › 4.png]

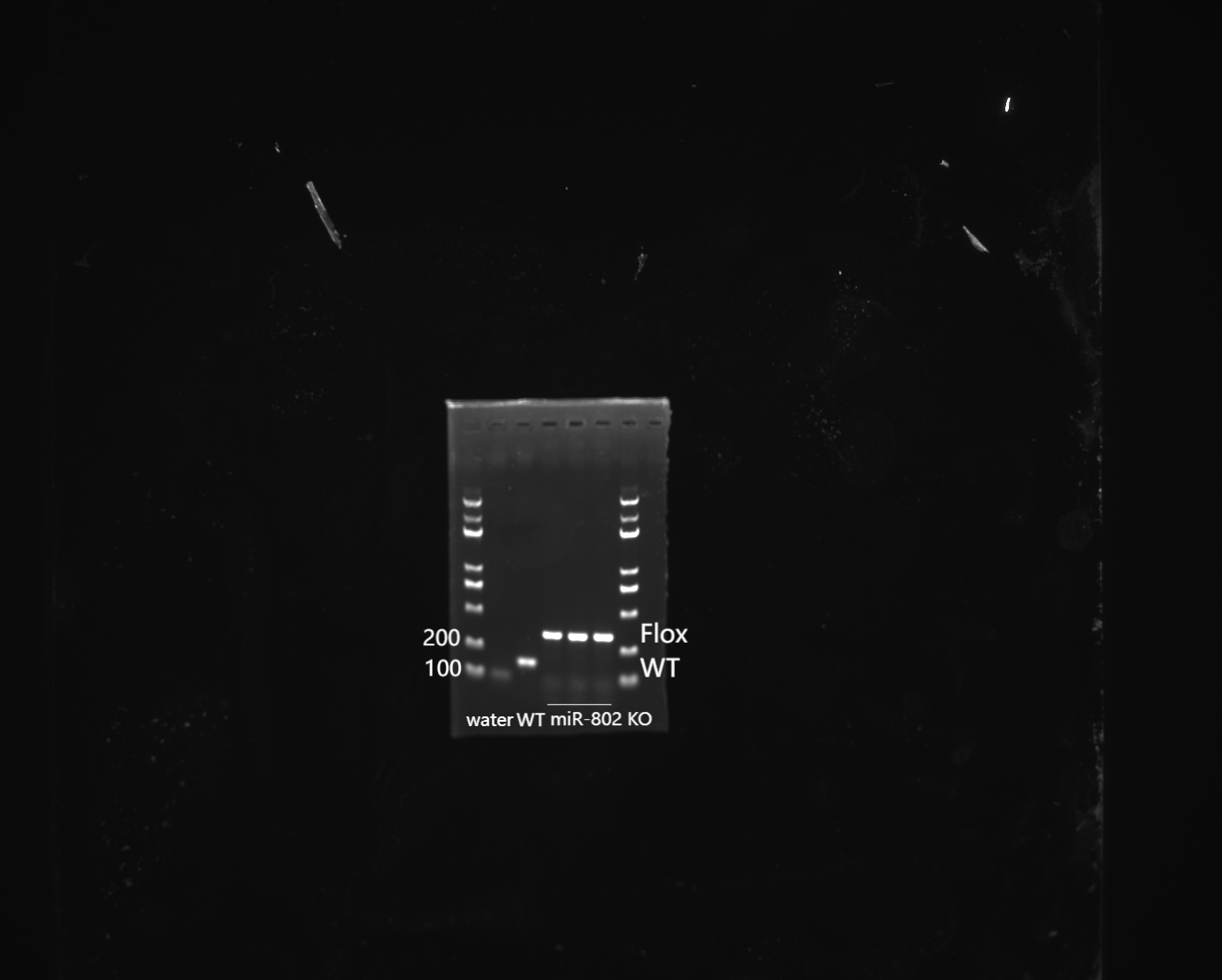

Supplement: Figure 3—figure supplement 1—source data 1. — The original files of the full raw unedited gels of Mir802 KO mice. [file elife-99162-fig3-figsupp1-data1.zip › 5.png]

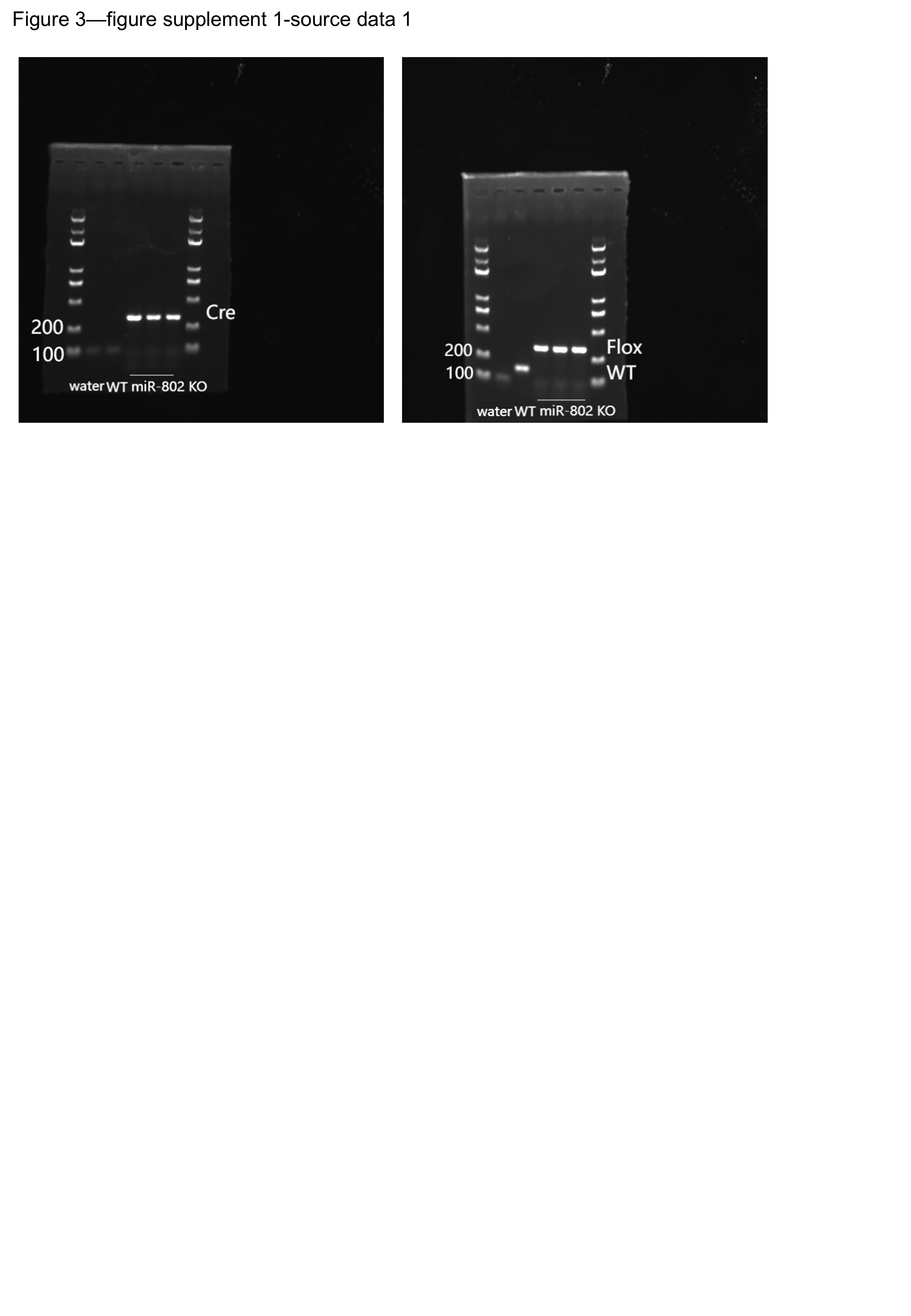

Supplement: Figure 3—figure supplement 1—source data 1. — The original files of the full raw unedited gels of Mir802 KO mice. [file elife-99162-fig3-figsupp1-data1.zip › Figure 3ΓÇöfigure supplement 1-source data 1.tif]

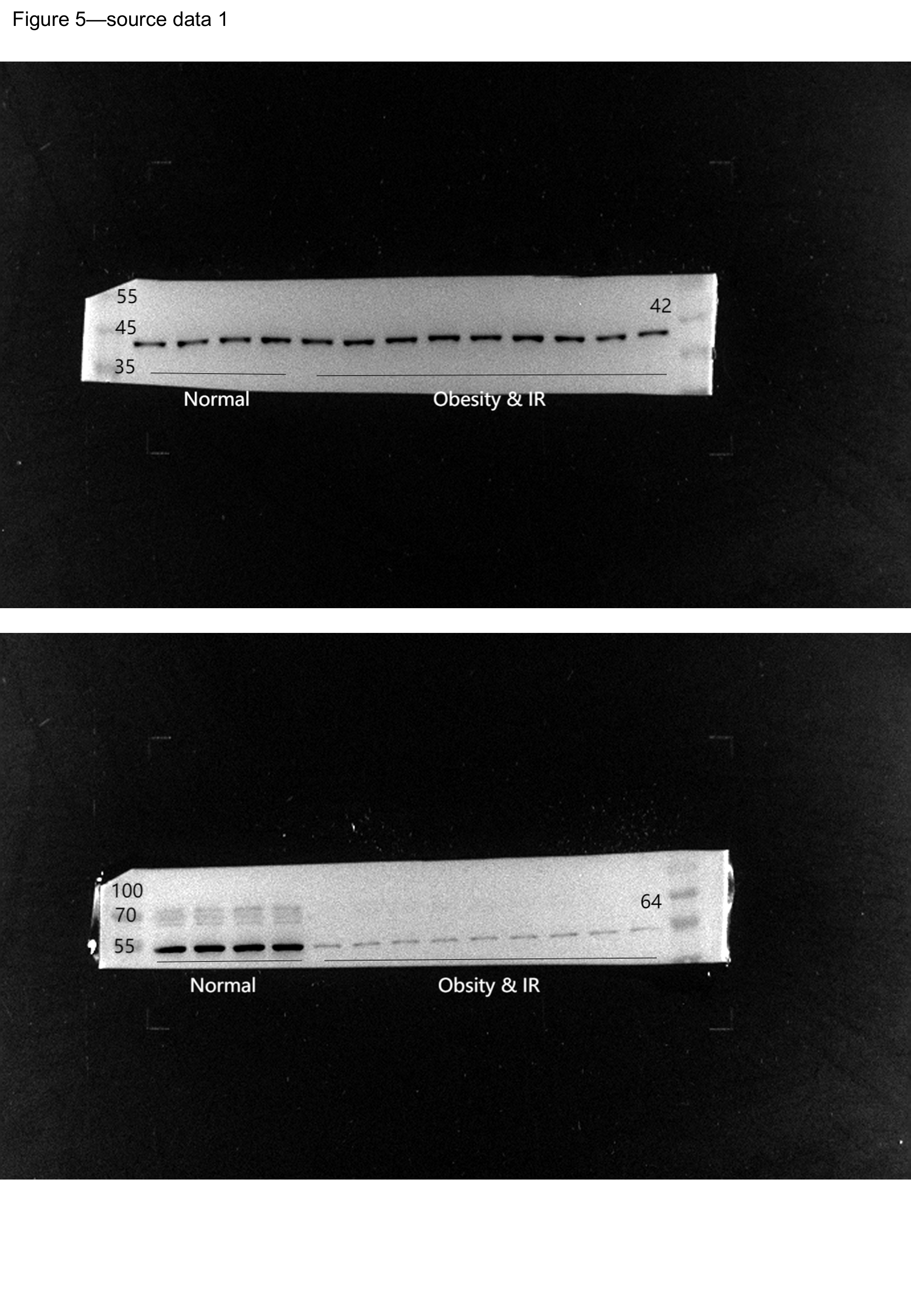

Supplement: Figure 5—source data 1. — The original files of the full raw unedited blots of TRAF3 and β-Actin in human subcutaneous adipose tissues from obese and normal individuals (nnormal = 4 and nobesity&IR=9). [file elife-99162-fig5-data1.zip › Figure 5ΓÇösource data 1.tif]

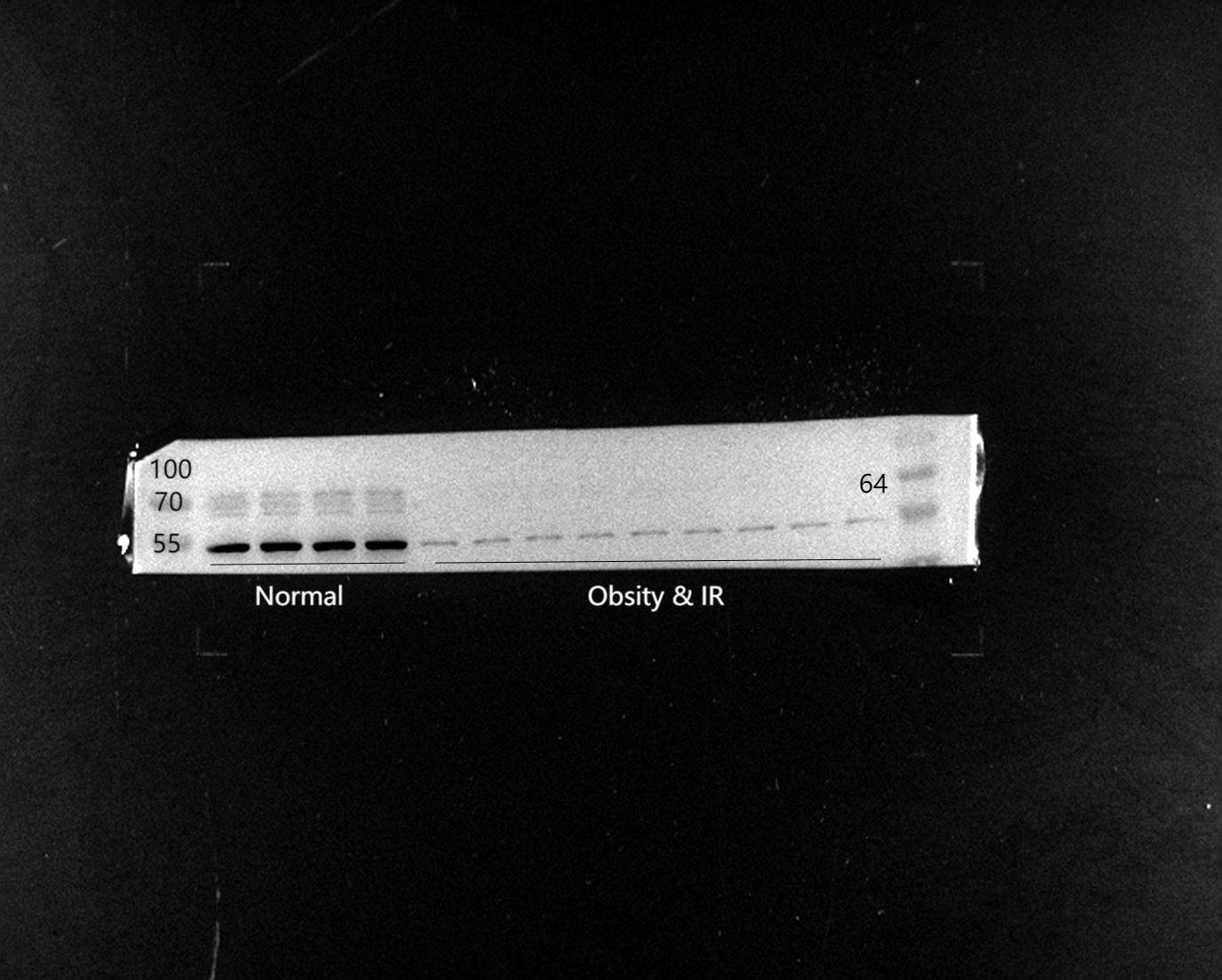

Supplement: Figure 5—source data 1. — The original files of the full raw unedited blots of TRAF3 and β-Actin in human subcutaneous adipose tissues from obese and normal individuals (nnormal = 4 and nobesity&IR=9). [file elife-99162-fig5-data1.zip › TRAF3.png]

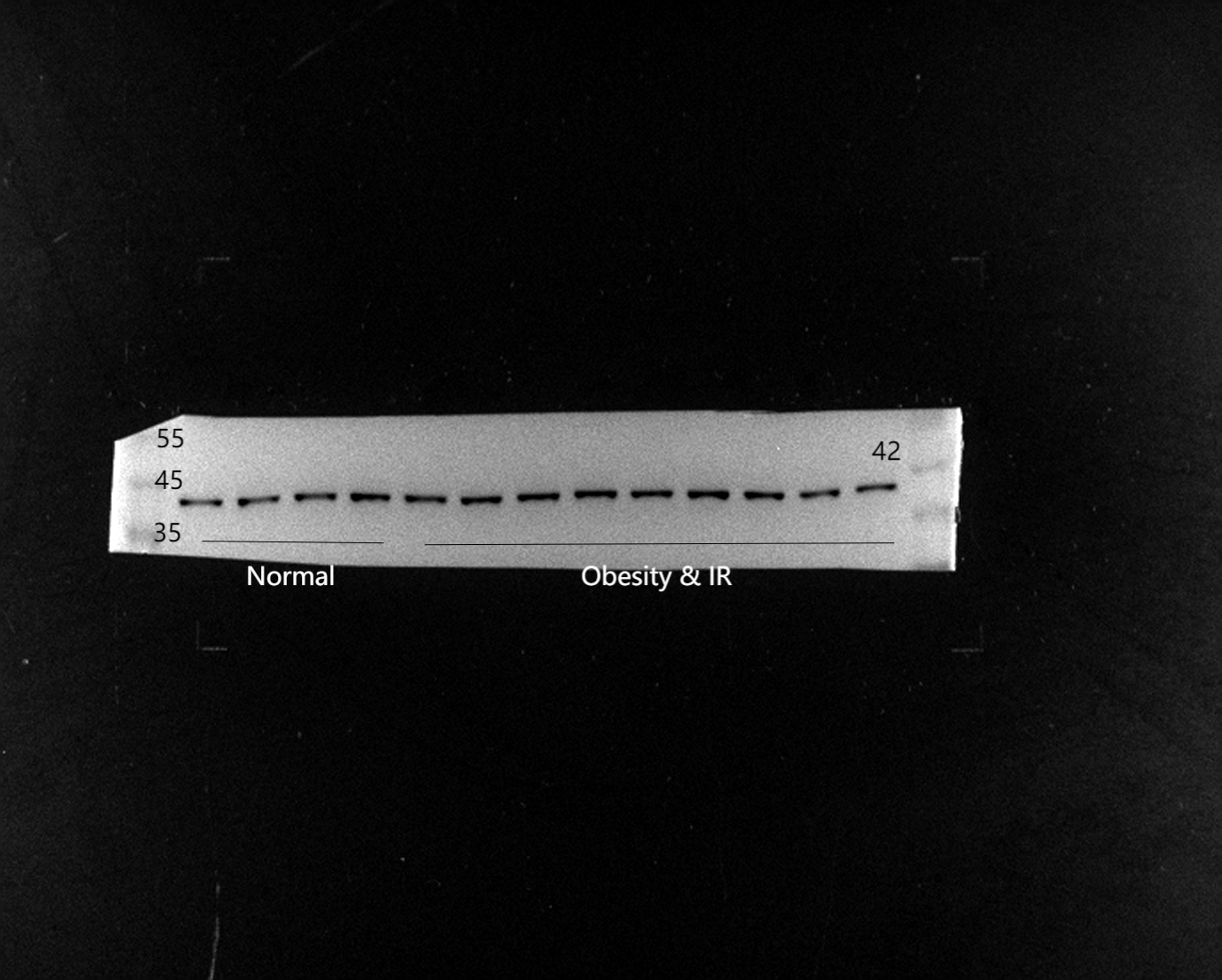

Supplement: Figure 5—source data 1. — The original files of the full raw unedited blots of TRAF3 and β-Actin in human subcutaneous adipose tissues from obese and normal individuals (nnormal = 4 and nobesity&IR=9). [file elife-99162-fig5-data1.zip › ╬▓-Actin.png]

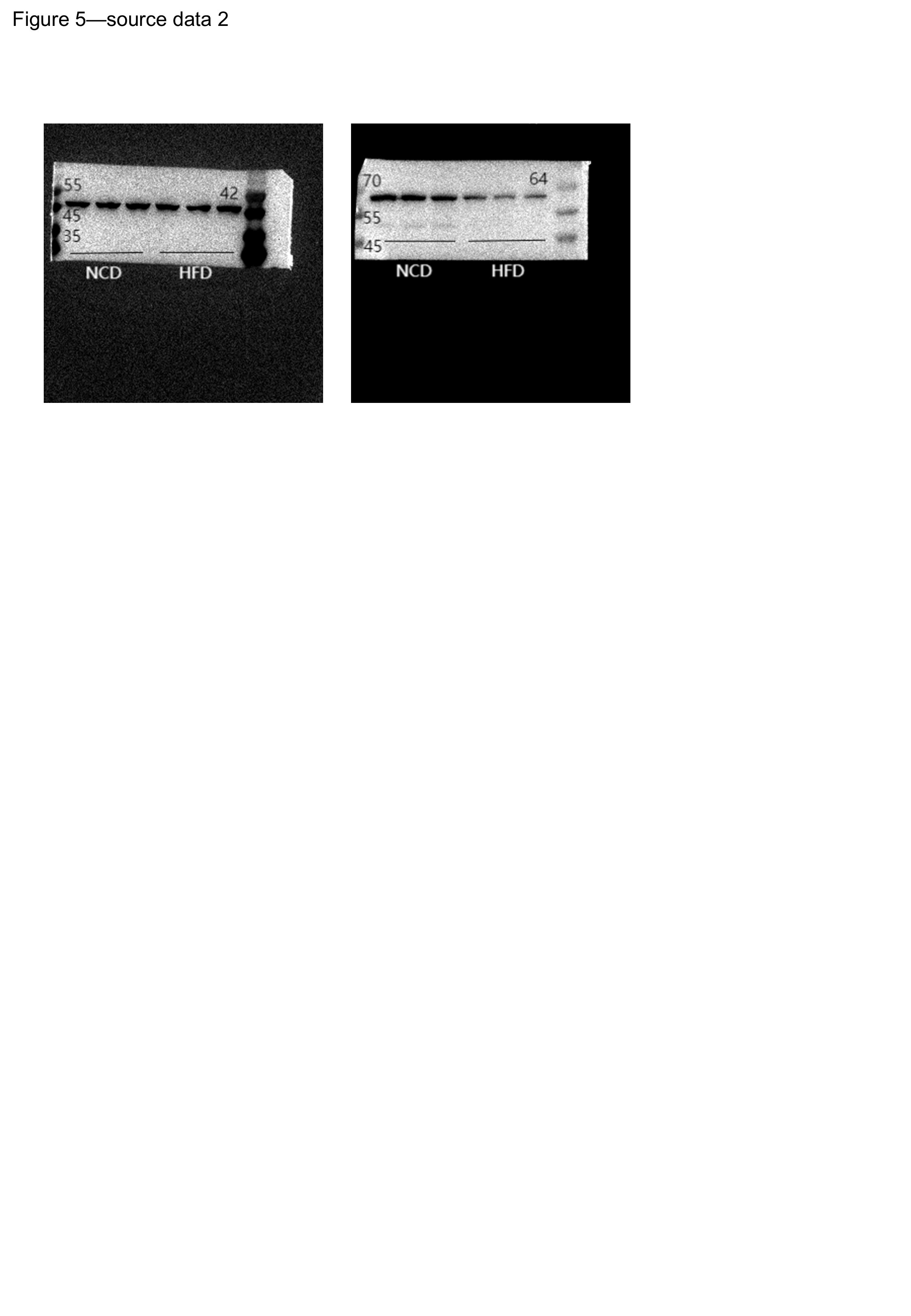

Supplement: Figure 5—source data 2. — The original files of the full raw unedited blots of TRAF3 and β-Actin in the epiWAT of HFD mice (n=3). [file elife-99162-fig5-data2.zip › Figure 5ΓÇösource data 2.tif]

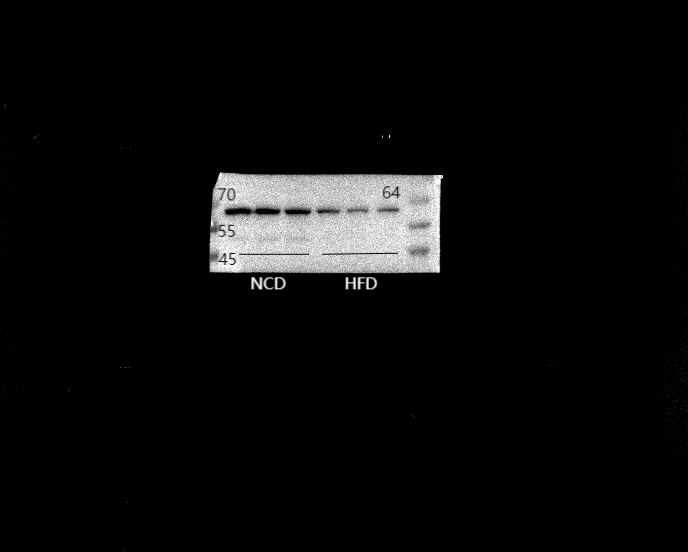

Supplement: Figure 5—source data 2. — The original files of the full raw unedited blots of TRAF3 and β-Actin in the epiWAT of HFD mice (n=3). [file elife-99162-fig5-data2.zip › TRAF3.png]

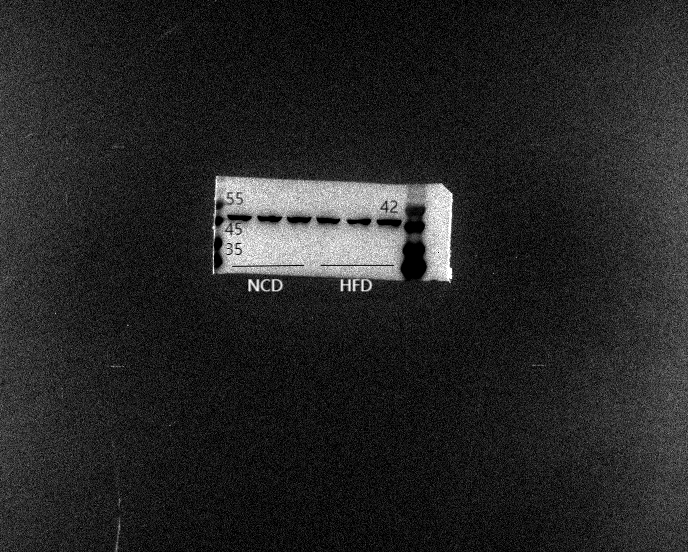

Supplement: Figure 5—source data 2. — The original files of the full raw unedited blots of TRAF3 and β-Actin in the epiWAT of HFD mice (n=3). [file elife-99162-fig5-data2.zip › ╬▓-Actin.png]

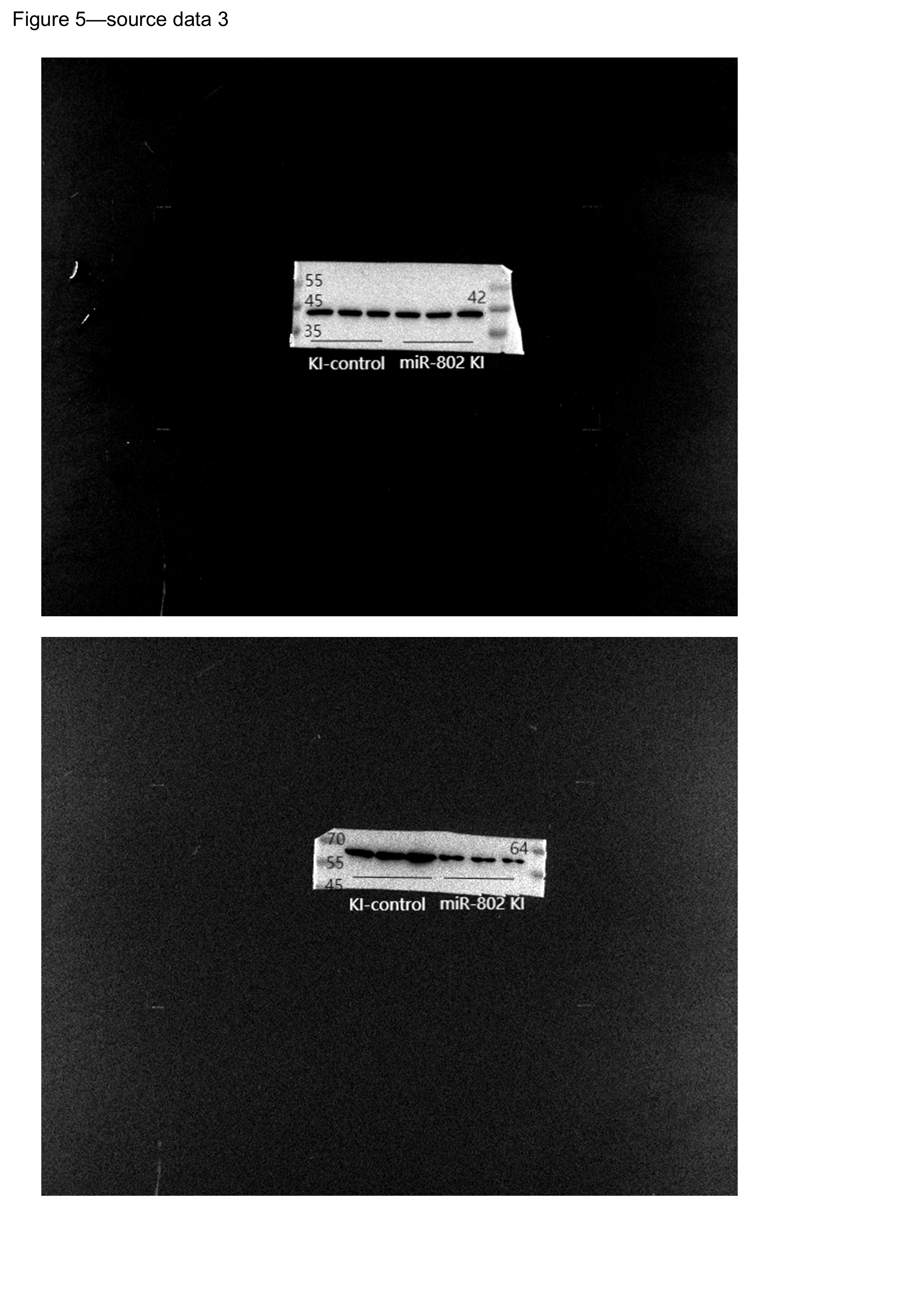

Supplement: Figure 5—source data 3. — The original files of the full raw unedited blots of TRAF3 and β-Actin in the epiWAT of Mir802 KI mice (n=3). [file elife-99162-fig5-data3.zip › Figure 5ΓÇösource data 3.tif]

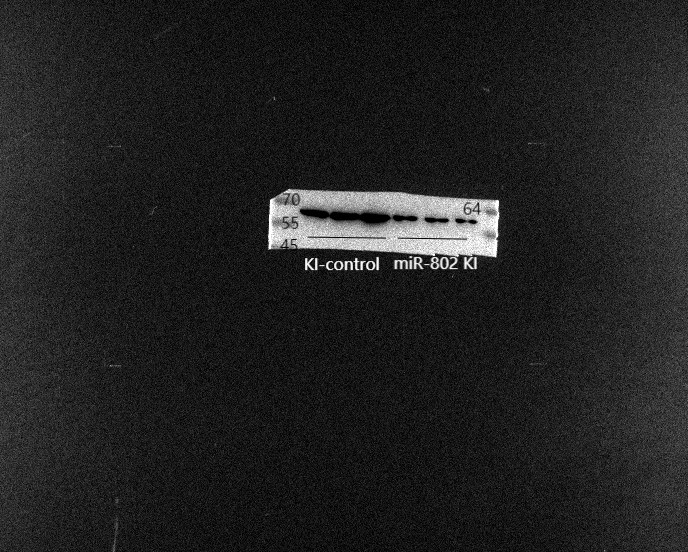

Supplement: Figure 5—source data 3. — The original files of the full raw unedited blots of TRAF3 and β-Actin in the epiWAT of Mir802 KI mice (n=3). [file elife-99162-fig5-data3.zip › TRAF3.png]

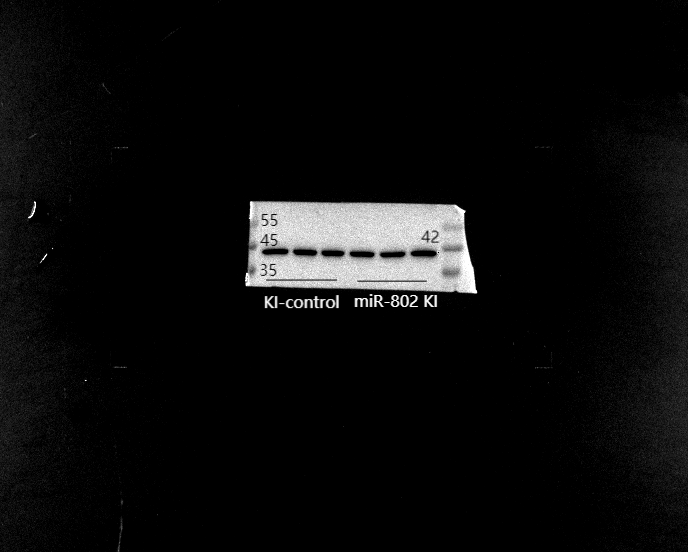

Supplement: Figure 5—source data 3. — The original files of the full raw unedited blots of TRAF3 and β-Actin in the epiWAT of Mir802 KI mice (n=3). [file elife-99162-fig5-data3.zip › ╬▓-Actin.png]

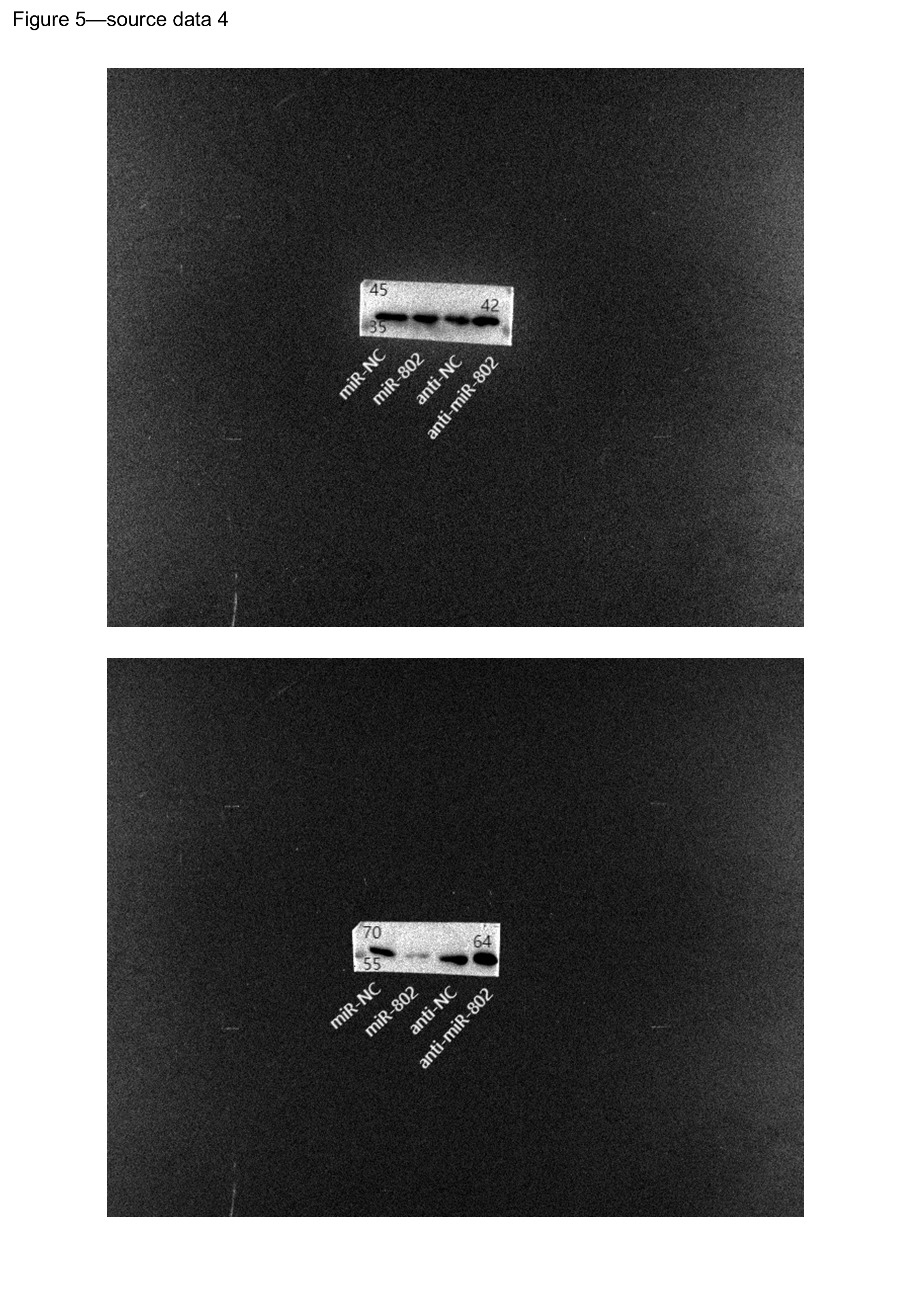

Supplement: Figure 5—source data 4. — The original files of the full raw unedited blots of TRAF3 and β-Actin in 3T3-L1 cells transfected with Mir802 mimics or Mir802 inhibitor. [file elife-99162-fig5-data4.zip › Figure 5ΓÇösource data 4.tif]

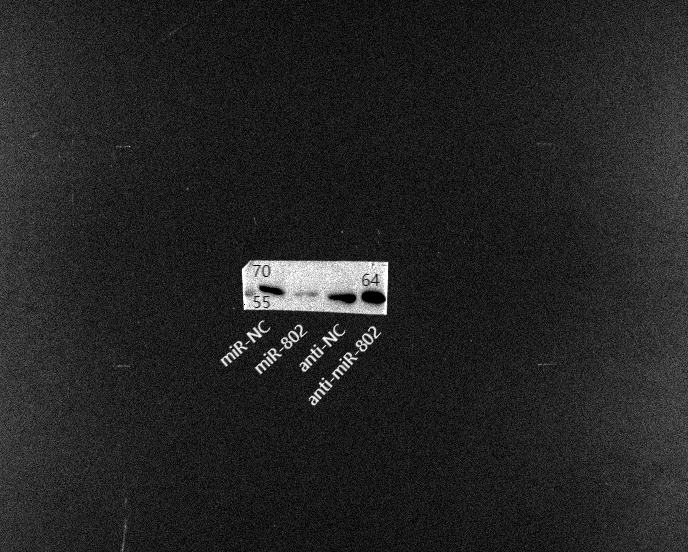

Supplement: Figure 5—source data 4. — The original files of the full raw unedited blots of TRAF3 and β-Actin in 3T3-L1 cells transfected with Mir802 mimics or Mir802 inhibitor. [file elife-99162-fig5-data4.zip › TRAF3.png]

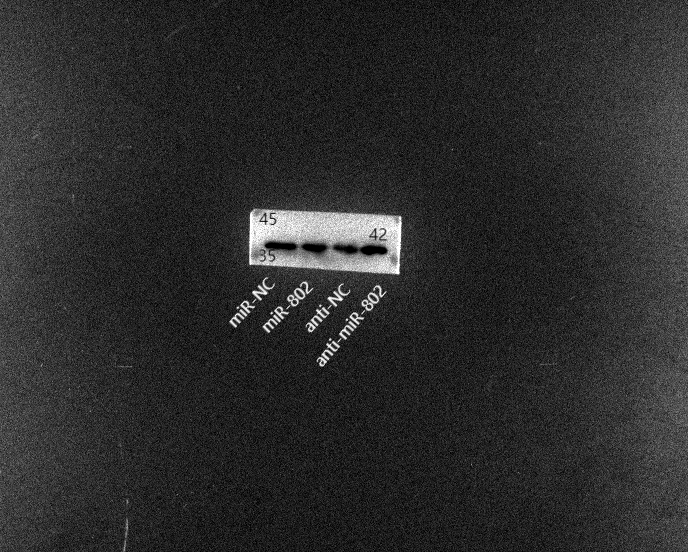

Supplement: Figure 5—source data 4. — The original files of the full raw unedited blots of TRAF3 and β-Actin in 3T3-L1 cells transfected with Mir802 mimics or Mir802 inhibitor. [file elife-99162-fig5-data4.zip › ╬▓-Actin.png]

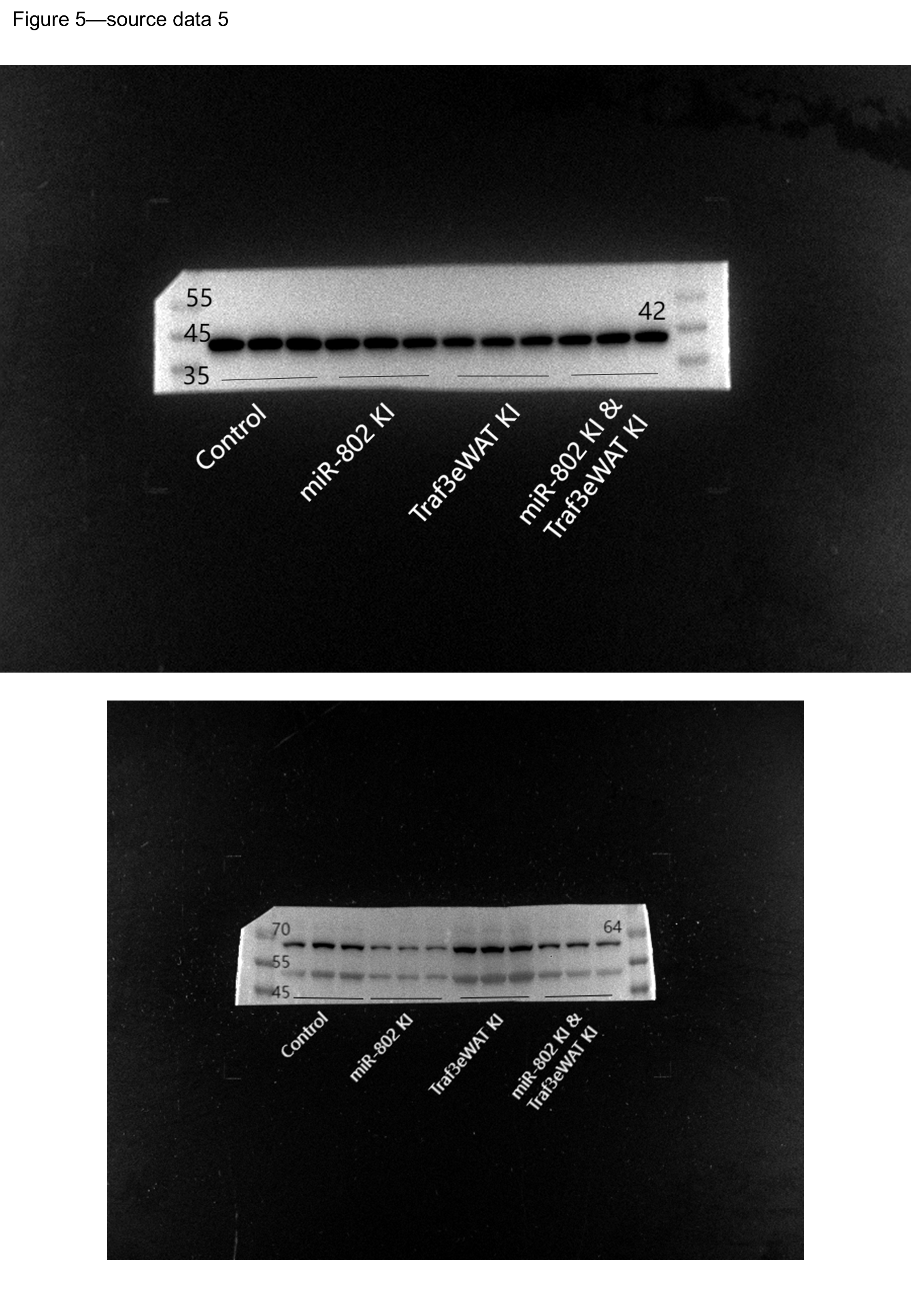

Supplement: Figure 5—source data 5. — The original files of the full raw unedited blots of TRAF3 and β-Actin in the epiWAT of control, Mir802 KI, Traf3 eWAT OE, and Mir802 KI and Traf3 eWAT OE mice (n=3). [file elife-99162-fig5-data5.zip › Figure 5ΓÇösource data 5.tif]

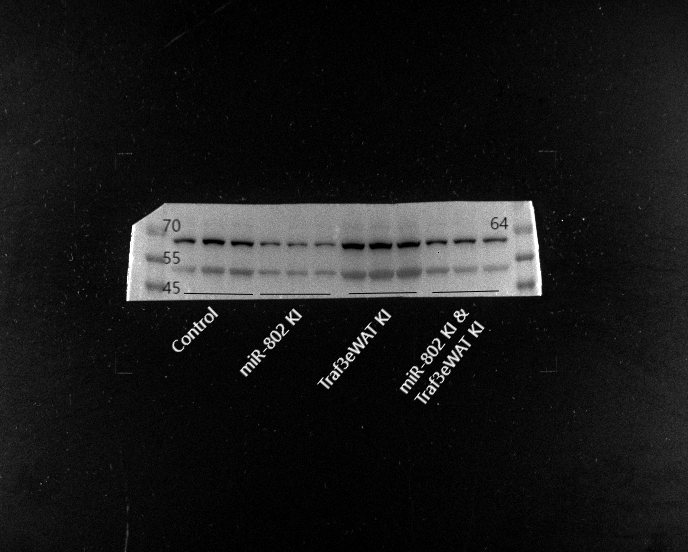

Supplement: Figure 5—source data 5. — The original files of the full raw unedited blots of TRAF3 and β-Actin in the epiWAT of control, Mir802 KI, Traf3 eWAT OE, and Mir802 KI and Traf3 eWAT OE mice (n=3). [file elife-99162-fig5-data5.zip › TRAF3.png]

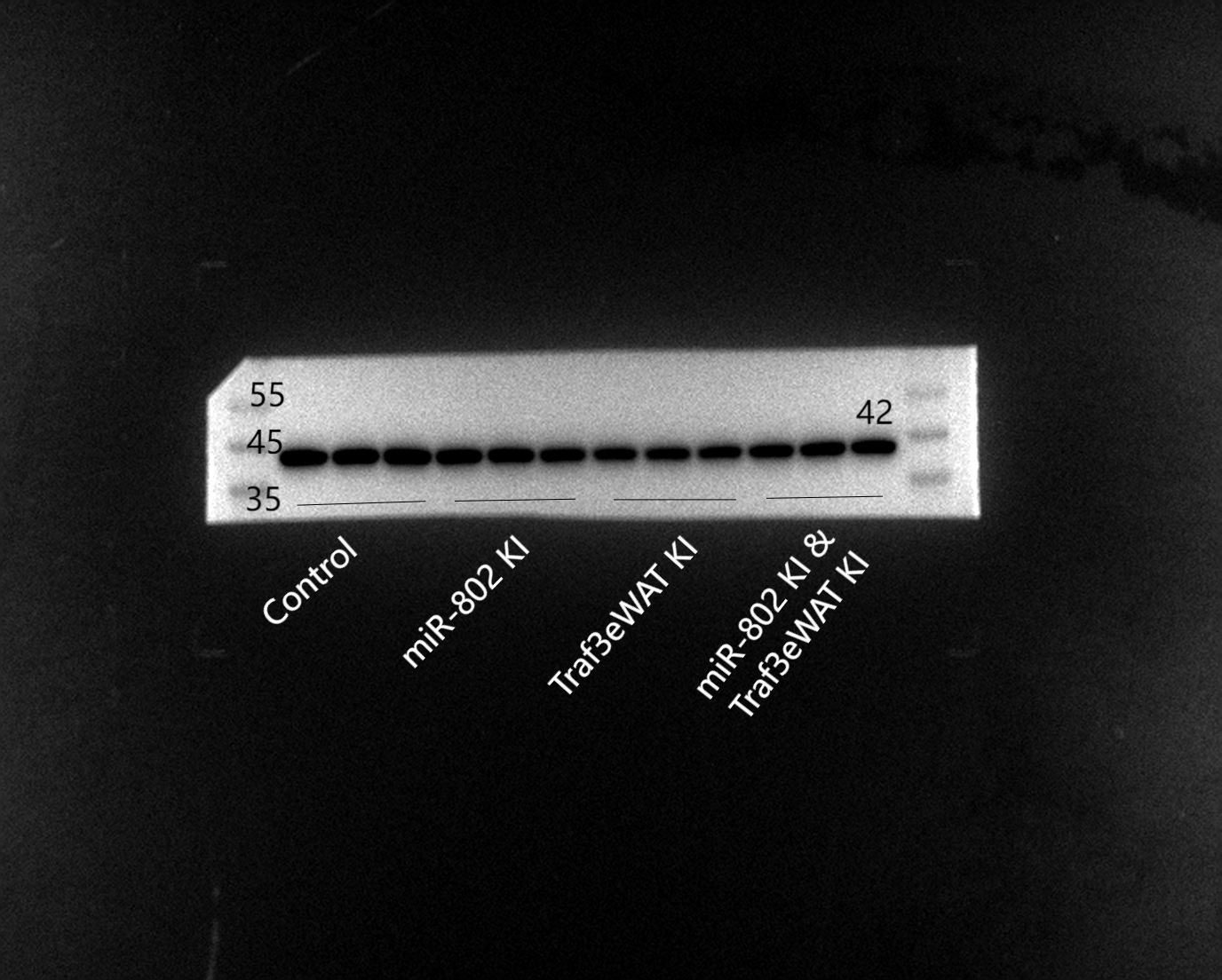

Supplement: Figure 5—source data 5. — The original files of the full raw unedited blots of TRAF3 and β-Actin in the epiWAT of control, Mir802 KI, Traf3 eWAT OE, and Mir802 KI and Traf3 eWAT OE mice (n=3). [file elife-99162-fig5-data5.zip › ╬▓-Actin.png]

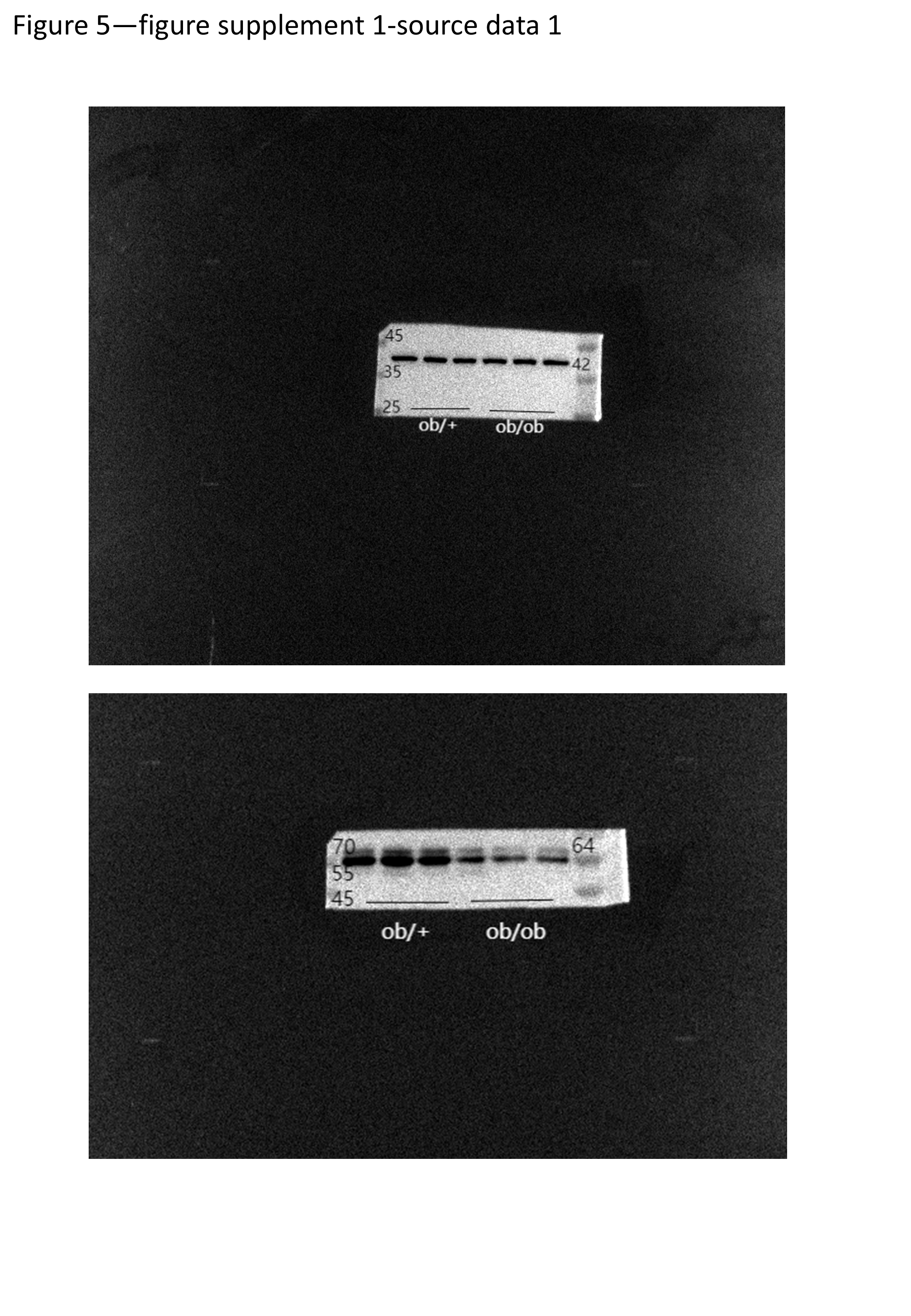

Supplement: Figure 5—figure supplement 1—source data 1. — The original files of the full raw unedited blots of TRAF3 and β-Actin in the epiWAT of Lepob/ob mice (n=3). [file elife-99162-fig5-figsupp1-data1.zip › Figure 5ΓÇöfigure supplement 1-source data 1.tif]

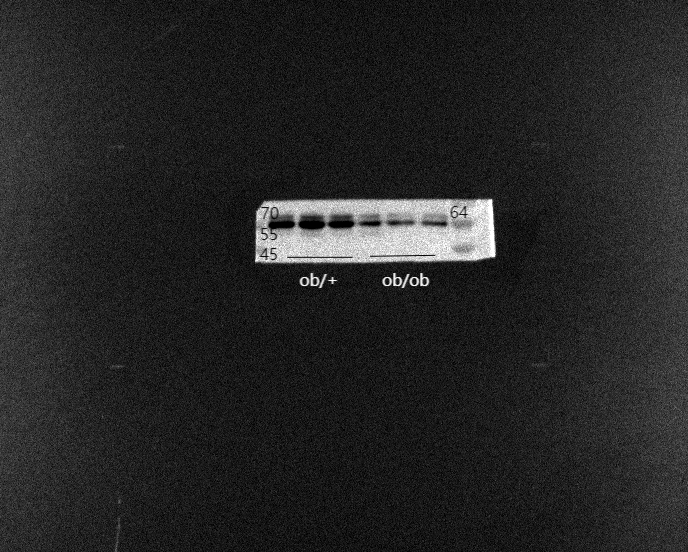

Supplement: Figure 5—figure supplement 1—source data 1. — The original files of the full raw unedited blots of TRAF3 and β-Actin in the epiWAT of Lepob/ob mice (n=3). [file elife-99162-fig5-figsupp1-data1.zip › TRAF3.png]

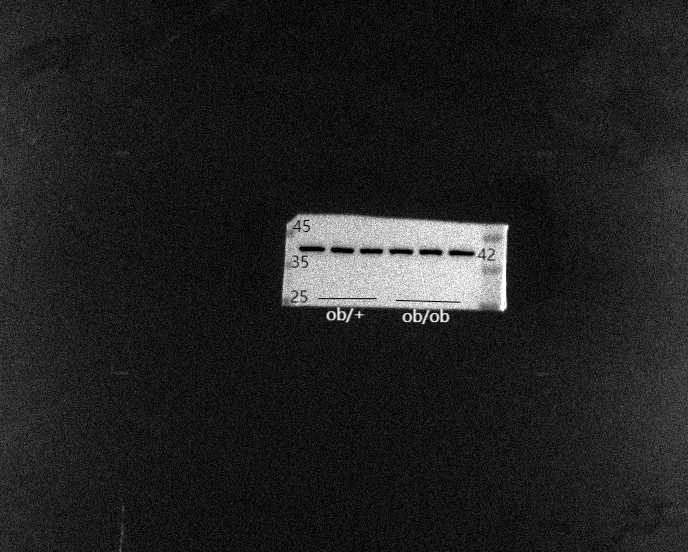

Supplement: Figure 5—figure supplement 1—source data 1. — The original files of the full raw unedited blots of TRAF3 and β-Actin in the epiWAT of Lepob/ob mice (n=3). [file elife-99162-fig5-figsupp1-data1.zip › ╬▓-Actin.png]

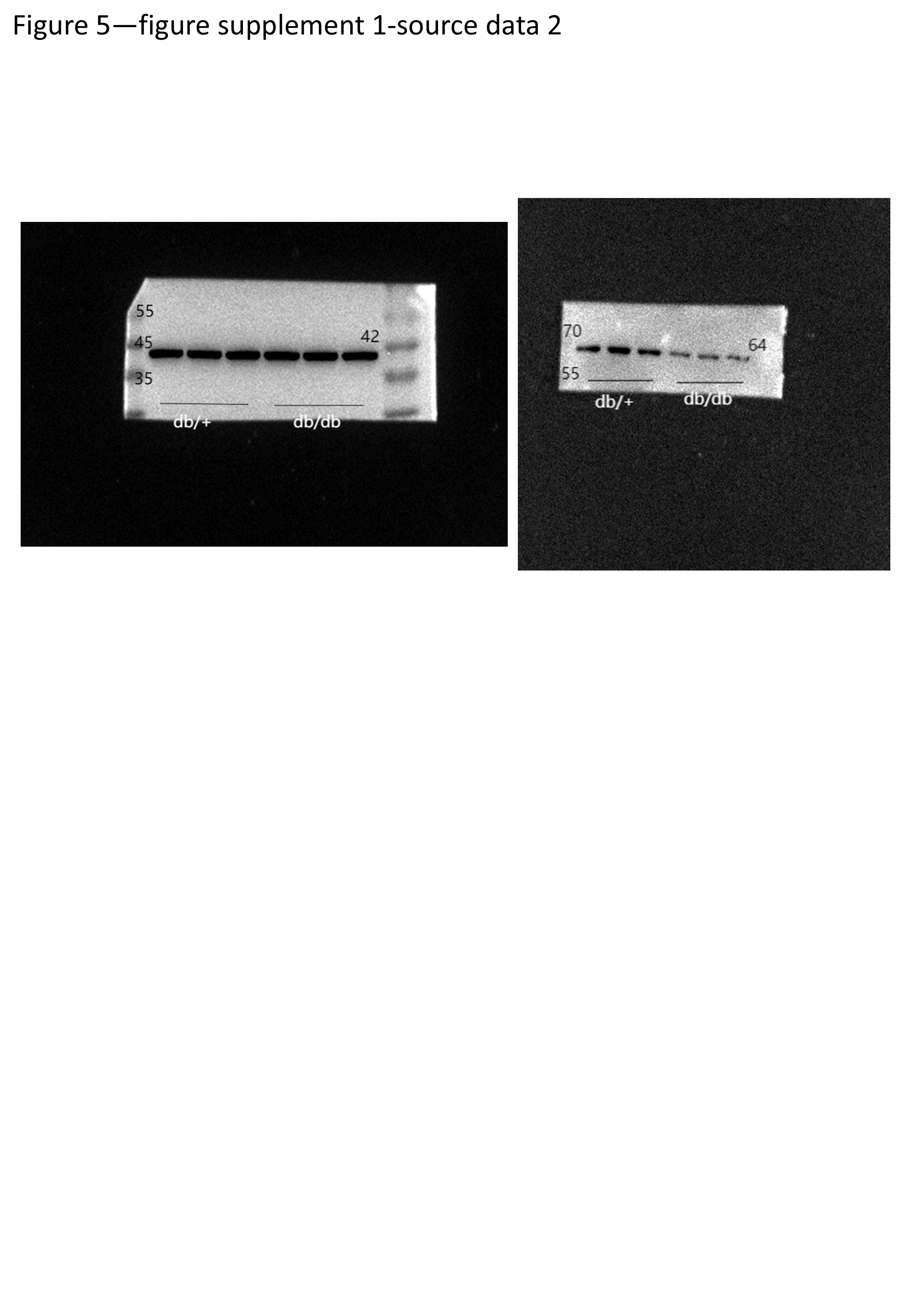

Supplement: Figure 5—figure supplement 1—source data 2. — The original files of the full raw unedited blots of TRAF3 and β-Actin in the epiWAT of Leprdb/db mice (n=3). [file elife-99162-fig5-figsupp1-data2.zip › Figure 5ΓÇöfigure supplement 1-source data 2.tif]

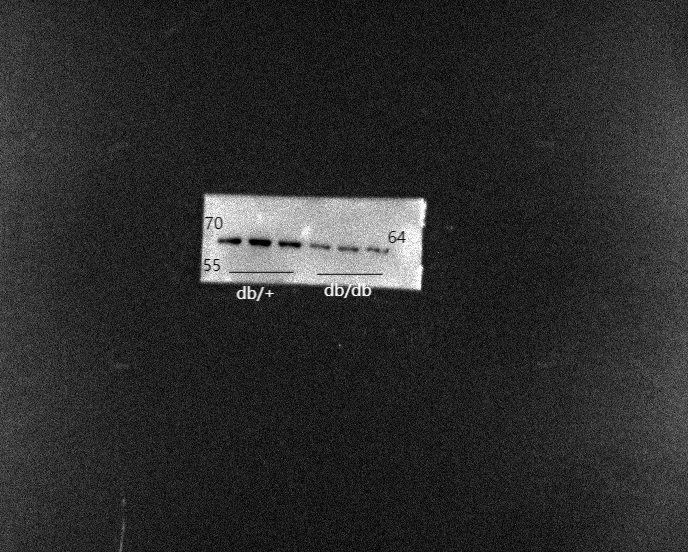

Supplement: Figure 5—figure supplement 1—source data 2. — The original files of the full raw unedited blots of TRAF3 and β-Actin in the epiWAT of Leprdb/db mice (n=3). [file elife-99162-fig5-figsupp1-data2.zip › TRAF3.png]

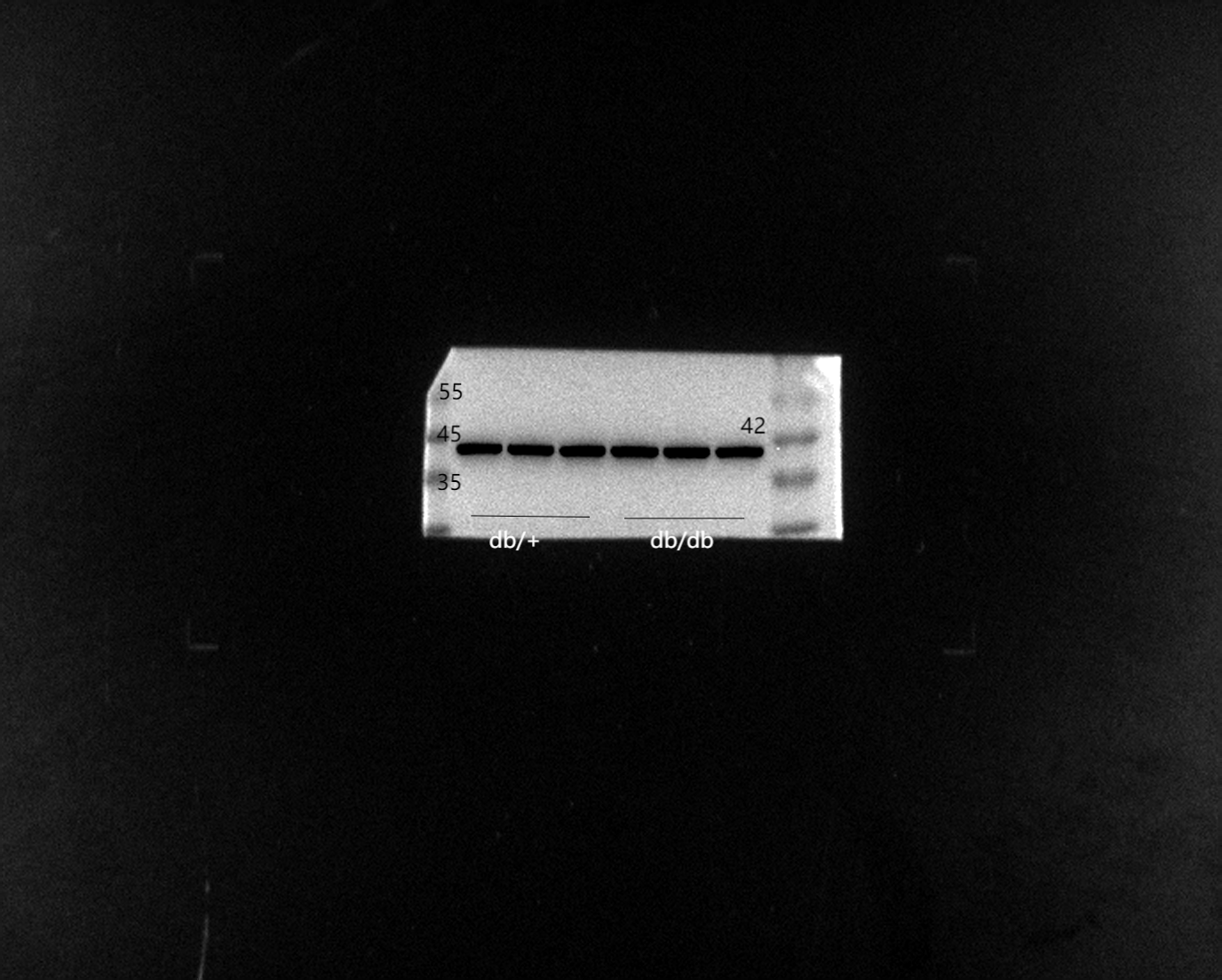

Supplement: Figure 5—figure supplement 1—source data 2. — The original files of the full raw unedited blots of TRAF3 and β-Actin in the epiWAT of Leprdb/db mice (n=3). [file elife-99162-fig5-figsupp1-data2.zip › ╬▓-Actin.png]

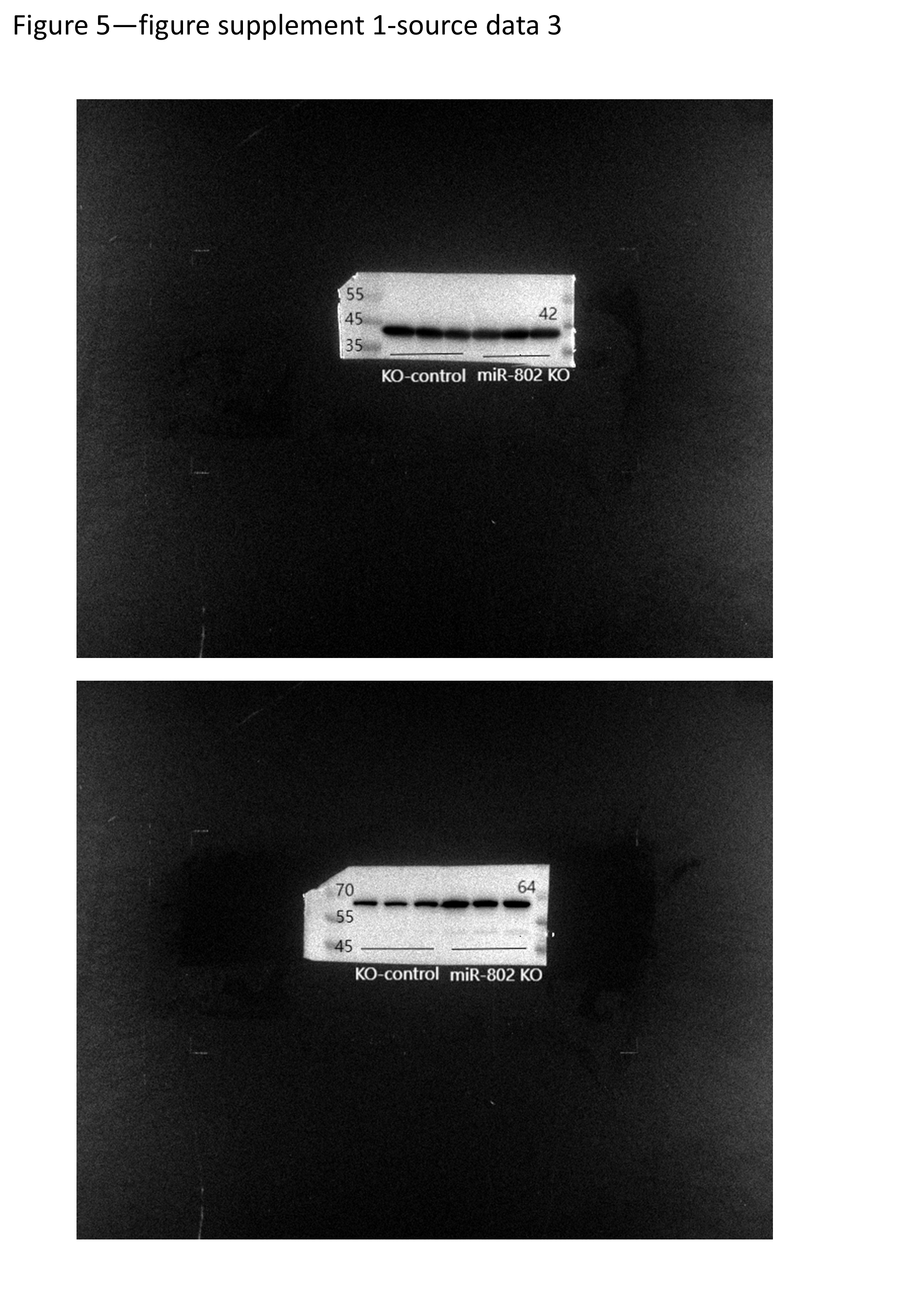

Supplement: Figure 5—figure supplement 1—source data 3. — The original files of the full raw unedited blots of TRAF3 and β-Actin in the epiWAT of Mir802 KO mice (n=3). [file elife-99162-fig5-figsupp1-data3.zip › Figure 5ΓÇöfigure supplement 1-source data 3.tif]

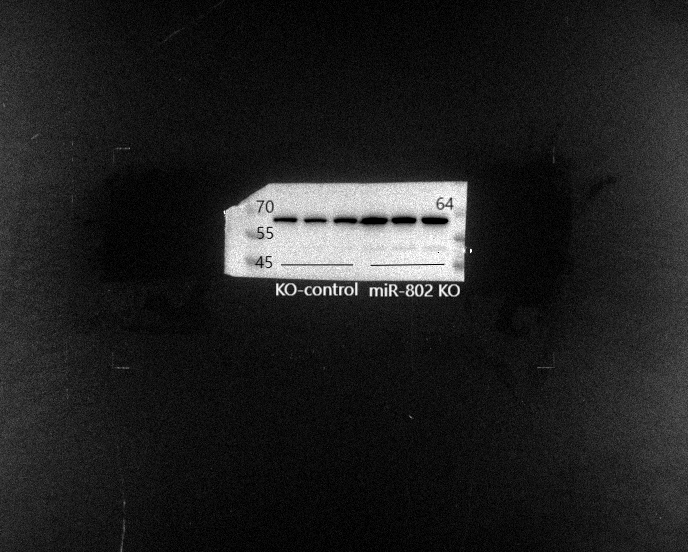

Supplement: Figure 5—figure supplement 1—source data 3. — The original files of the full raw unedited blots of TRAF3 and β-Actin in the epiWAT of Mir802 KO mice (n=3). [file elife-99162-fig5-figsupp1-data3.zip › TRAF3.png]

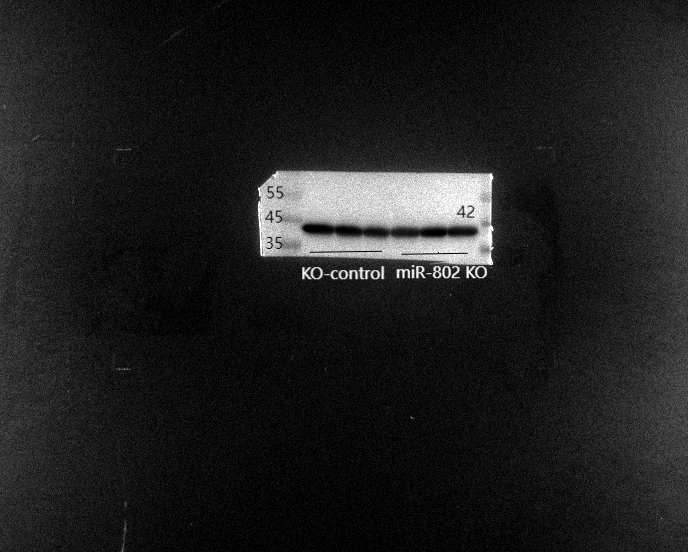

Supplement: Figure 5—figure supplement 1—source data 3. — The original files of the full raw unedited blots of TRAF3 and β-Actin in the epiWAT of Mir802 KO mice (n=3). [file elife-99162-fig5-figsupp1-data3.zip › ╬▓-Actin.png]

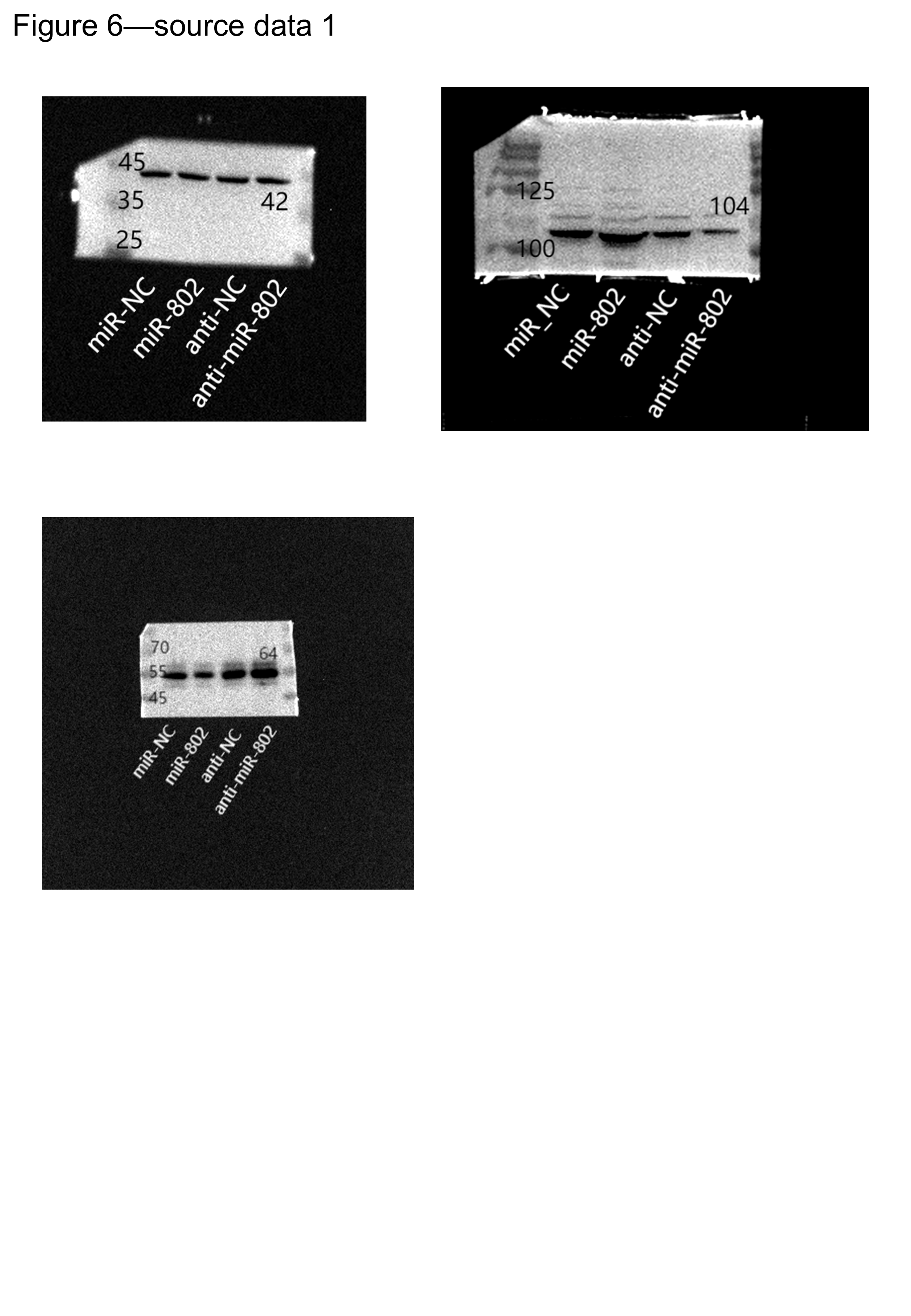

Supplement: Figure 6—source data 1. — The original files of the full raw unedited blots of TRAF3, NIK, and β-Actin in 3T3-L1 cells transfected with Mir802 mimics or Mir802 inhibitor. [file elife-99162-fig6-data1.zip › Figure 6ΓÇösource data 1.tif]

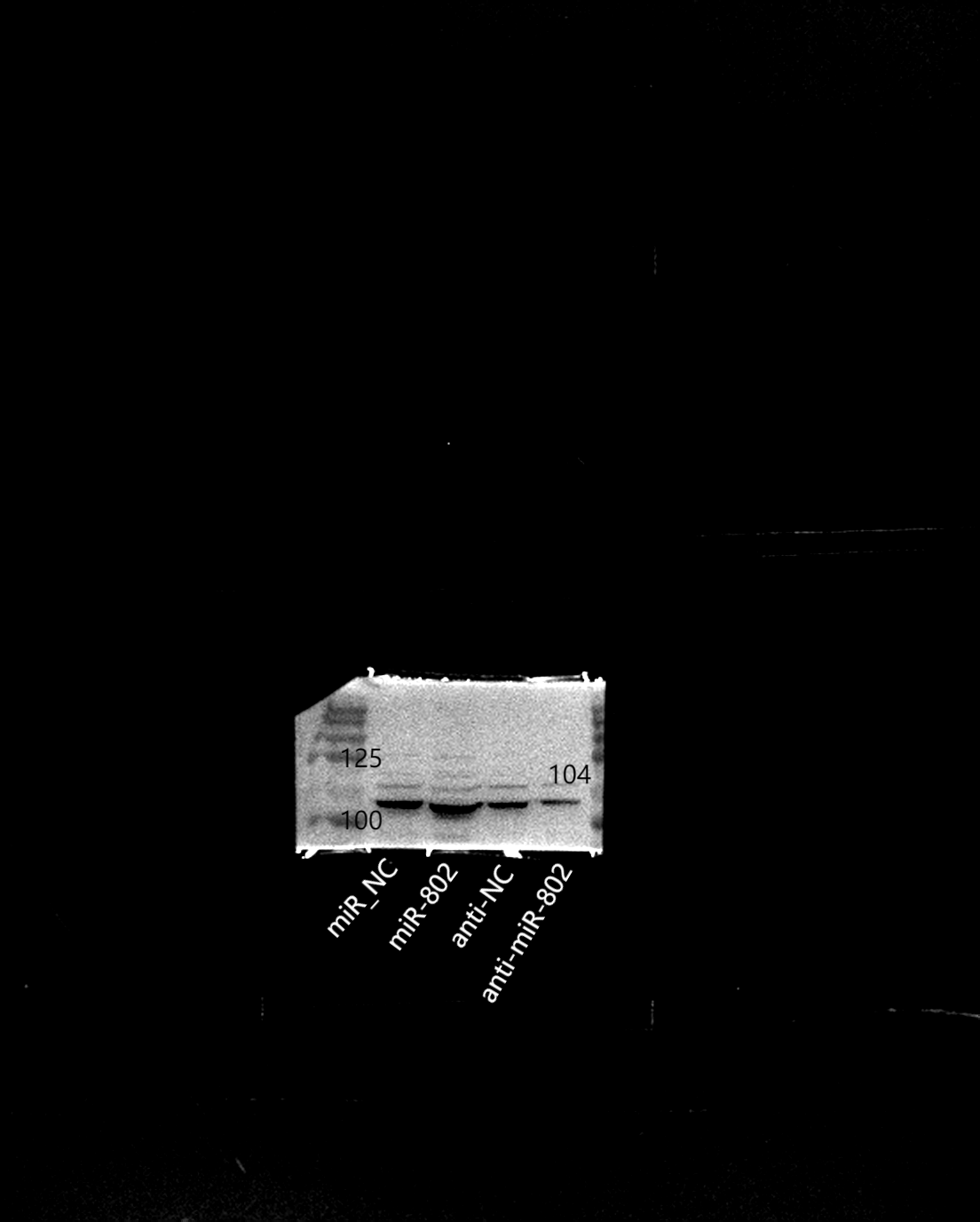

Supplement: Figure 6—source data 1. — The original files of the full raw unedited blots of TRAF3, NIK, and β-Actin in 3T3-L1 cells transfected with Mir802 mimics or Mir802 inhibitor. [file elife-99162-fig6-data1.zip › NIK.png]

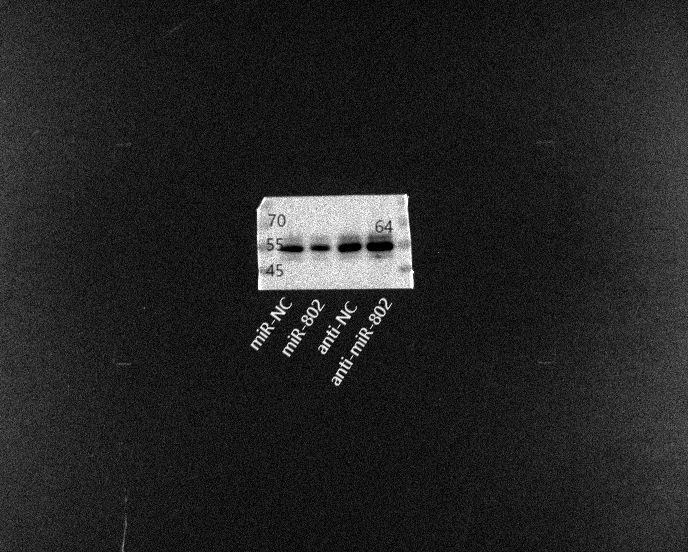

Supplement: Figure 6—source data 1. — The original files of the full raw unedited blots of TRAF3, NIK, and β-Actin in 3T3-L1 cells transfected with Mir802 mimics or Mir802 inhibitor. [file elife-99162-fig6-data1.zip › TRAF3.png]

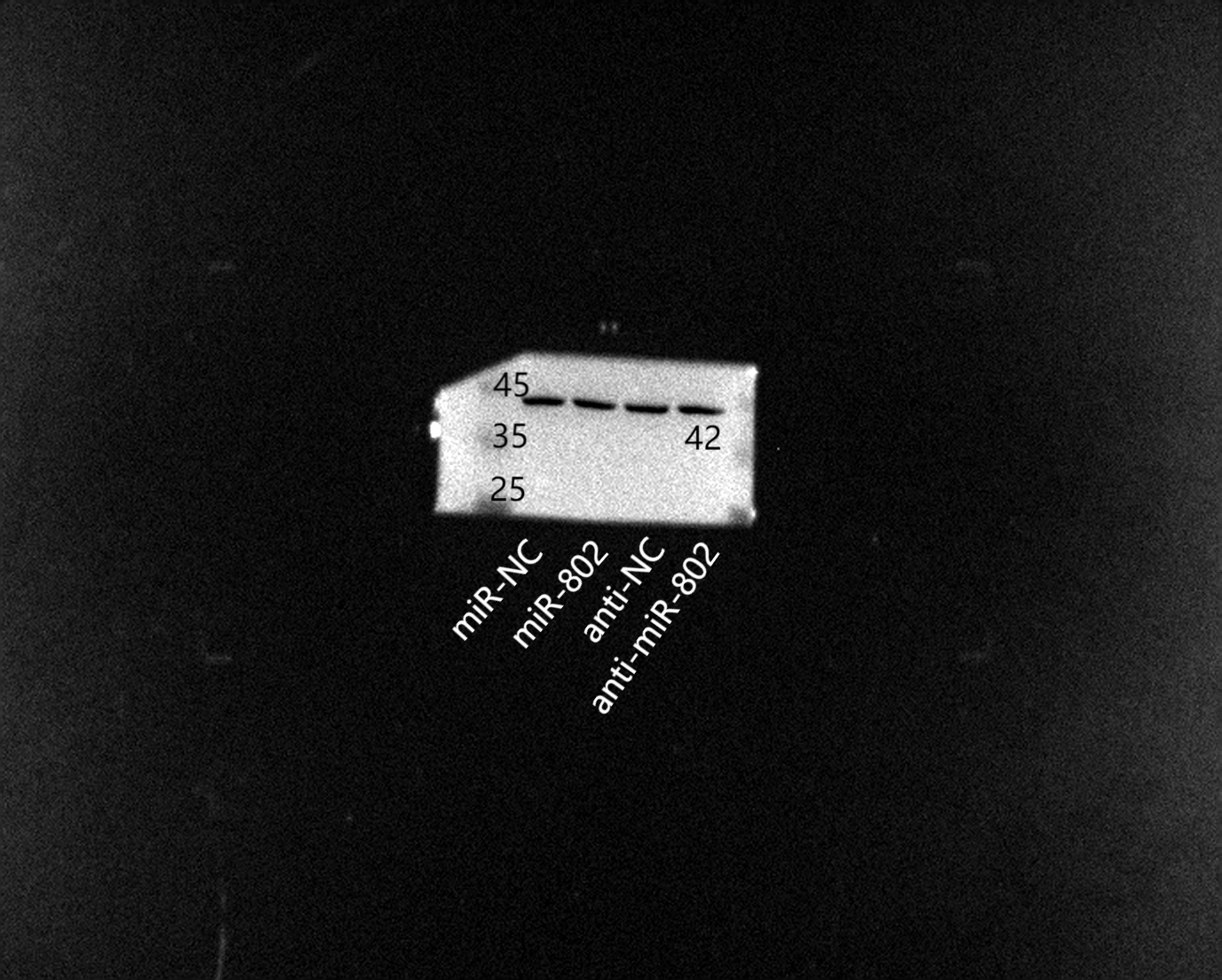

Supplement: Figure 6—source data 1. — The original files of the full raw unedited blots of TRAF3, NIK, and β-Actin in 3T3-L1 cells transfected with Mir802 mimics or Mir802 inhibitor. [file elife-99162-fig6-data1.zip › ╬▓-Actin.png]

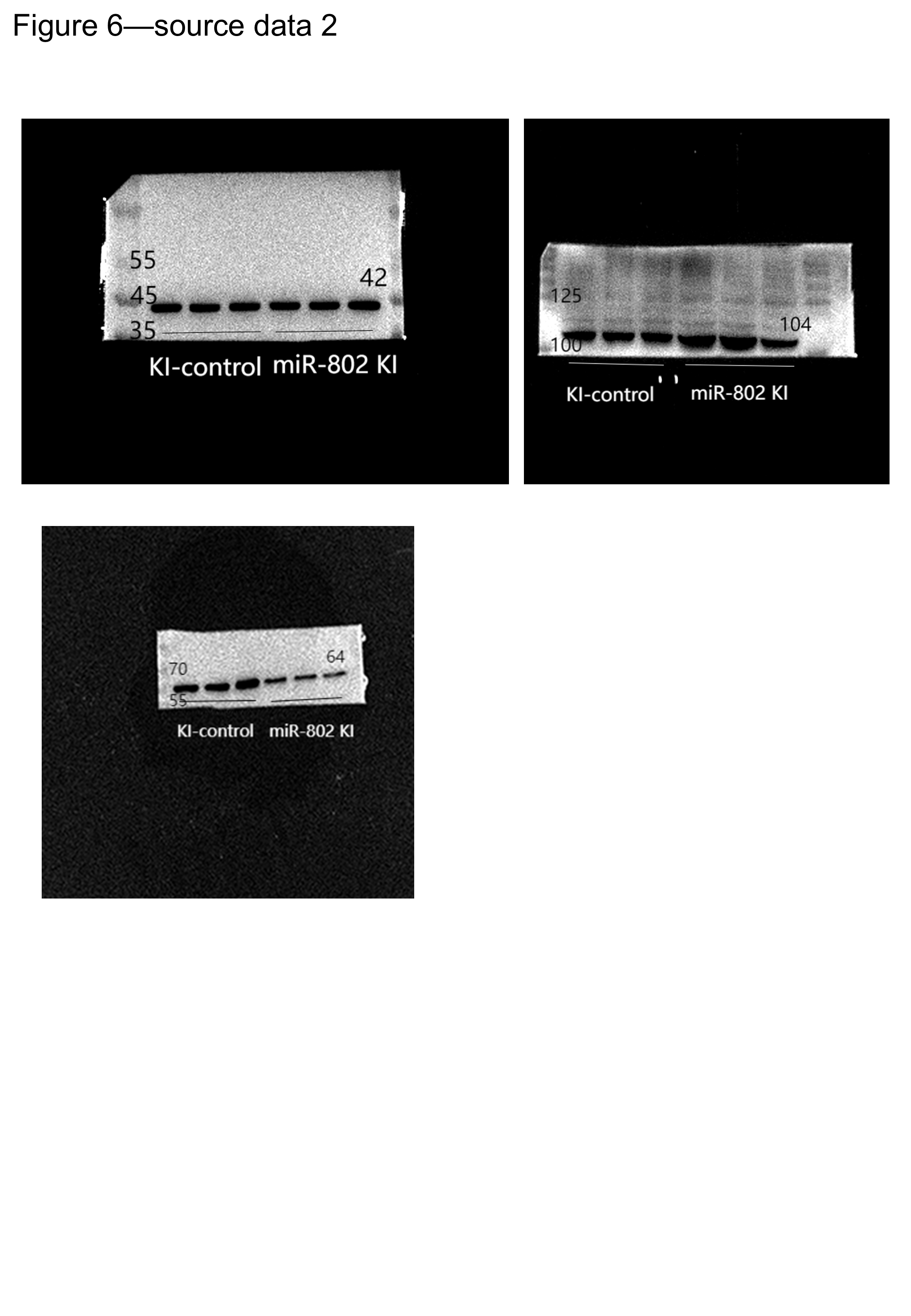

Supplement: Figure 6—source data 2. — The original files of the full raw unedited blots of TRAF3, NIK, and β-Actin in the epiWAT of Mir802 KI mice (n=3). [file elife-99162-fig6-data2.zip › Figure 6ΓÇösource data 2.tif]

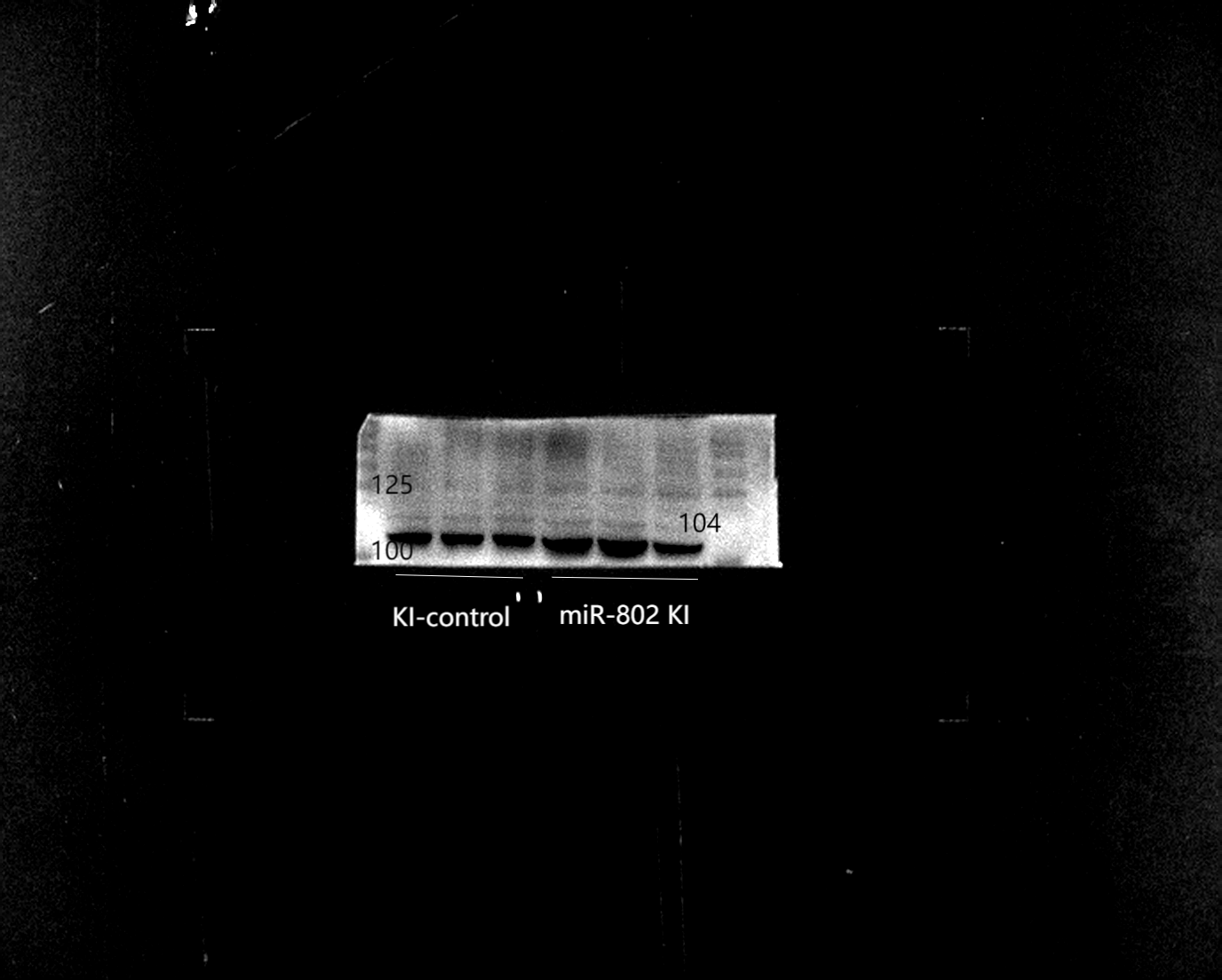

Supplement: Figure 6—source data 2. — The original files of the full raw unedited blots of TRAF3, NIK, and β-Actin in the epiWAT of Mir802 KI mice (n=3). [file elife-99162-fig6-data2.zip › NIK.png]

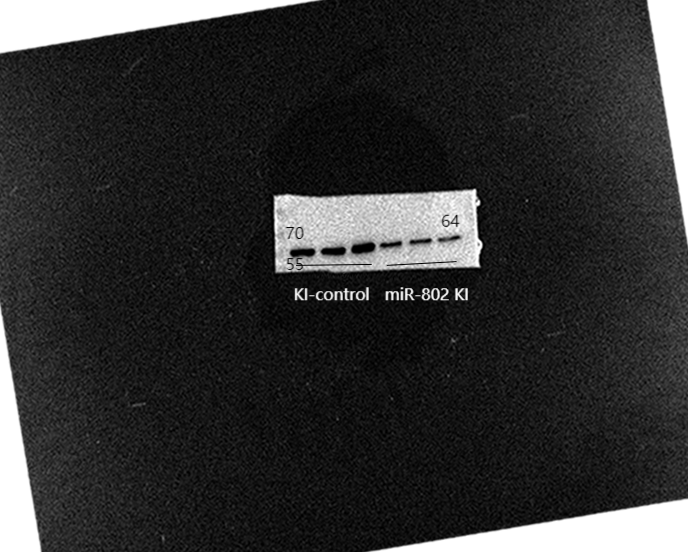

Supplement: Figure 6—source data 2. — The original files of the full raw unedited blots of TRAF3, NIK, and β-Actin in the epiWAT of Mir802 KI mice (n=3). [file elife-99162-fig6-data2.zip › TRAF3.png]

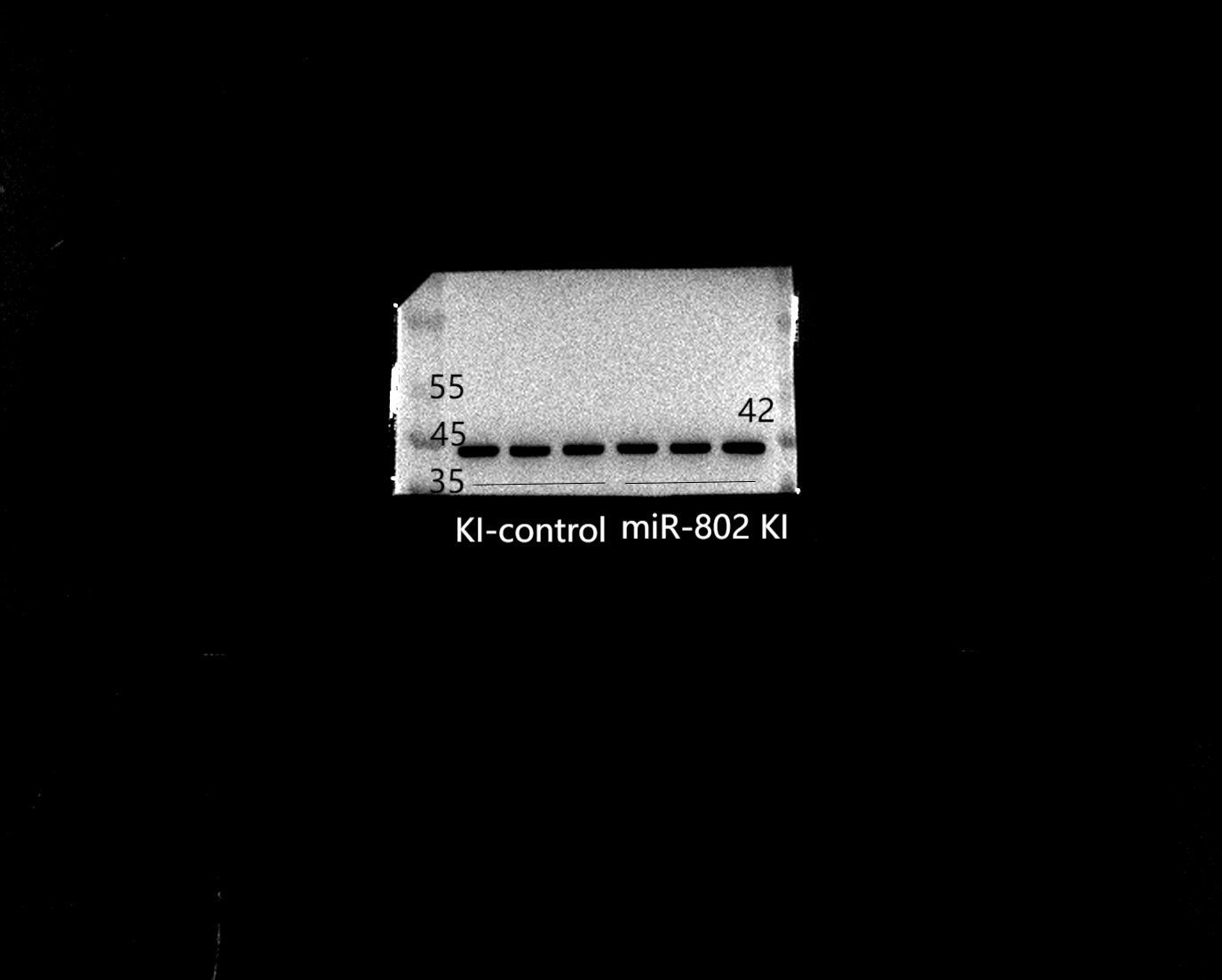

Supplement: Figure 6—source data 2. — The original files of the full raw unedited blots of TRAF3, NIK, and β-Actin in the epiWAT of Mir802 KI mice (n=3). [file elife-99162-fig6-data2.zip › ╬▓-Actin.png]

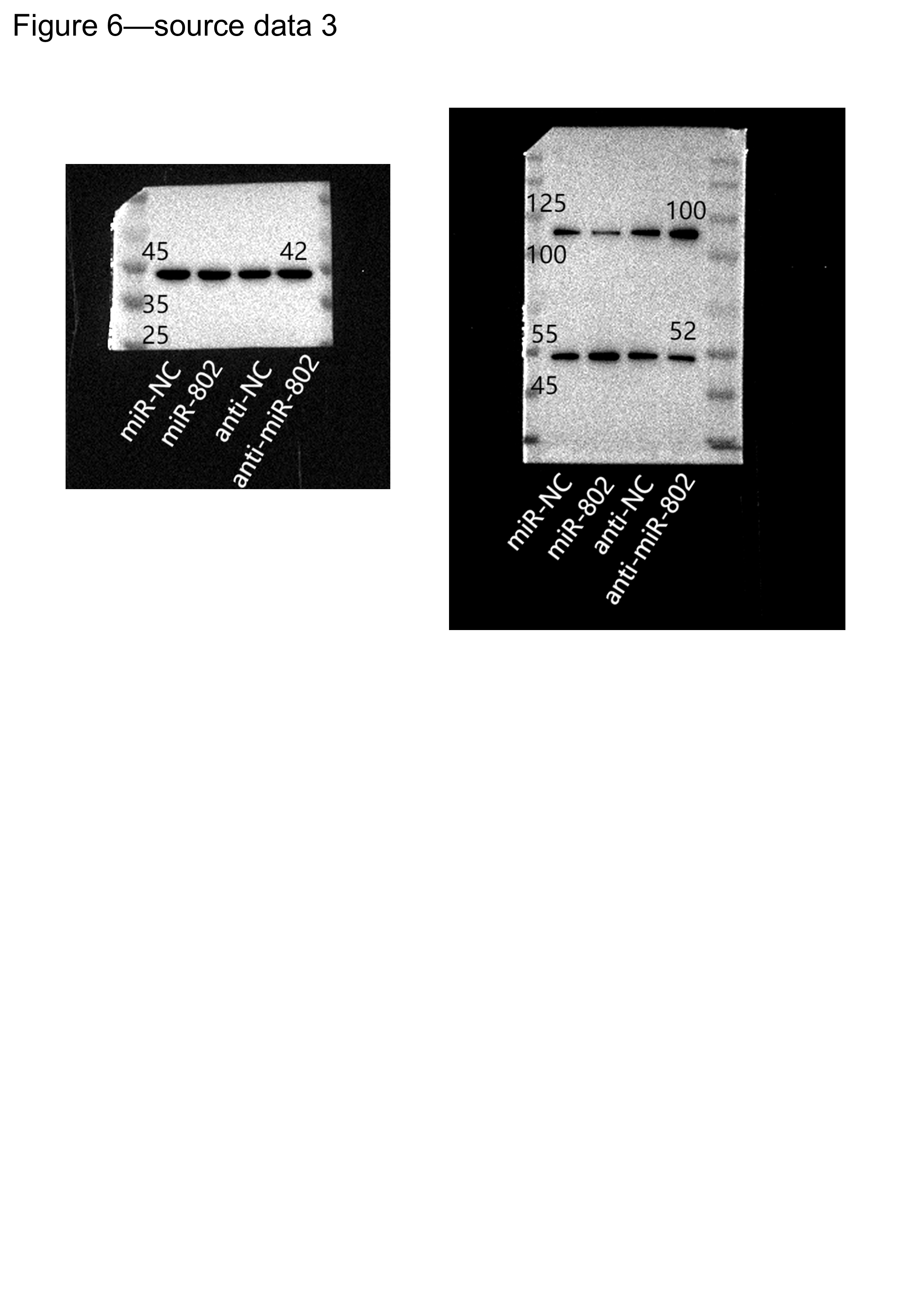

Supplement: Figure 6—source data 3. — The original files of the full raw unedited blots of p100/p52 and β-Actin in 3T3-L1 cells transfected with Mir802 mimics or Mir802 inhibitor. [file elife-99162-fig6-data3.zip › Figure 6ΓÇösource data 3.tif]

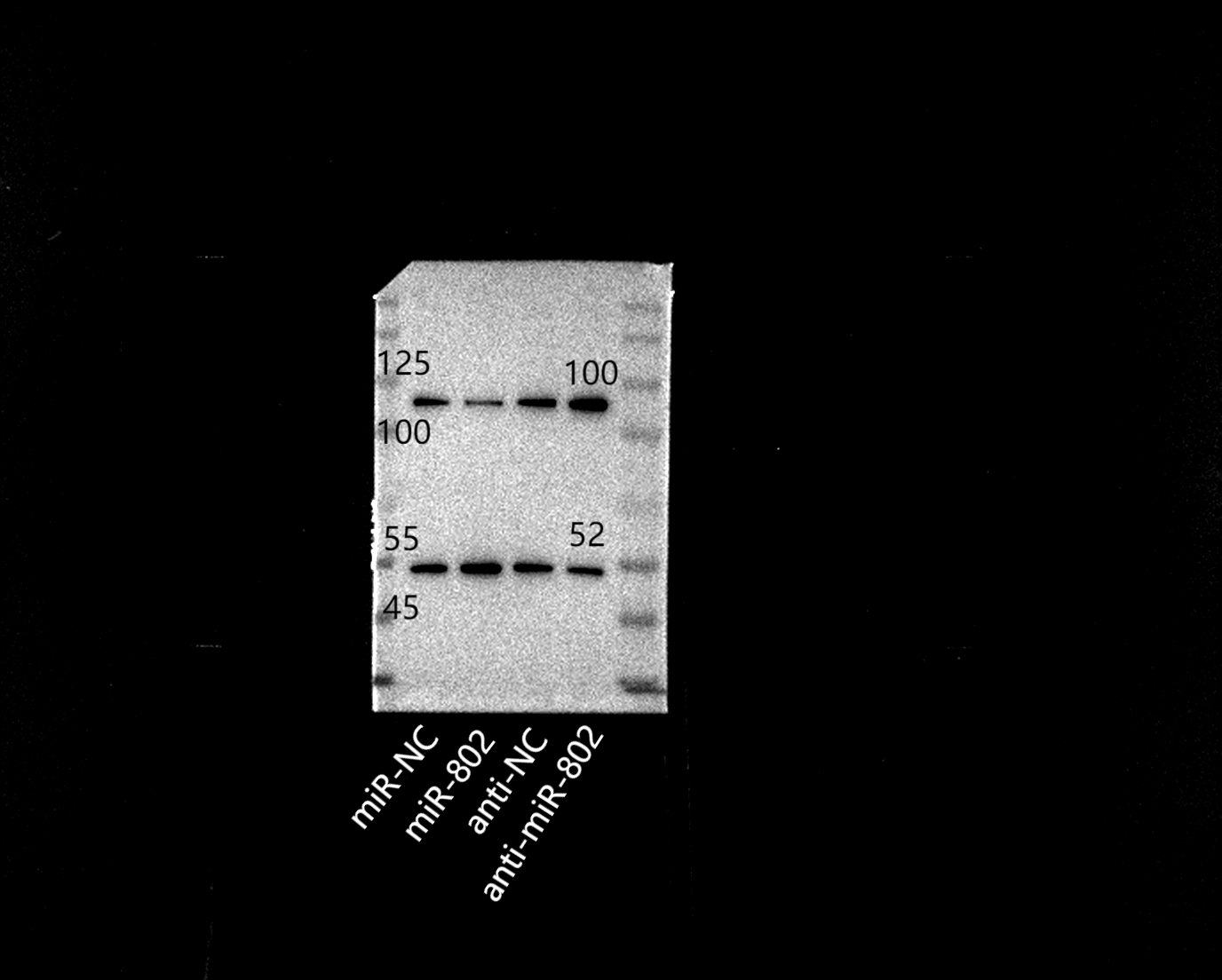

Supplement: Figure 6—source data 3. — The original files of the full raw unedited blots of p100/p52 and β-Actin in 3T3-L1 cells transfected with Mir802 mimics or Mir802 inhibitor. [file elife-99162-fig6-data3.zip › p100-p52.png]

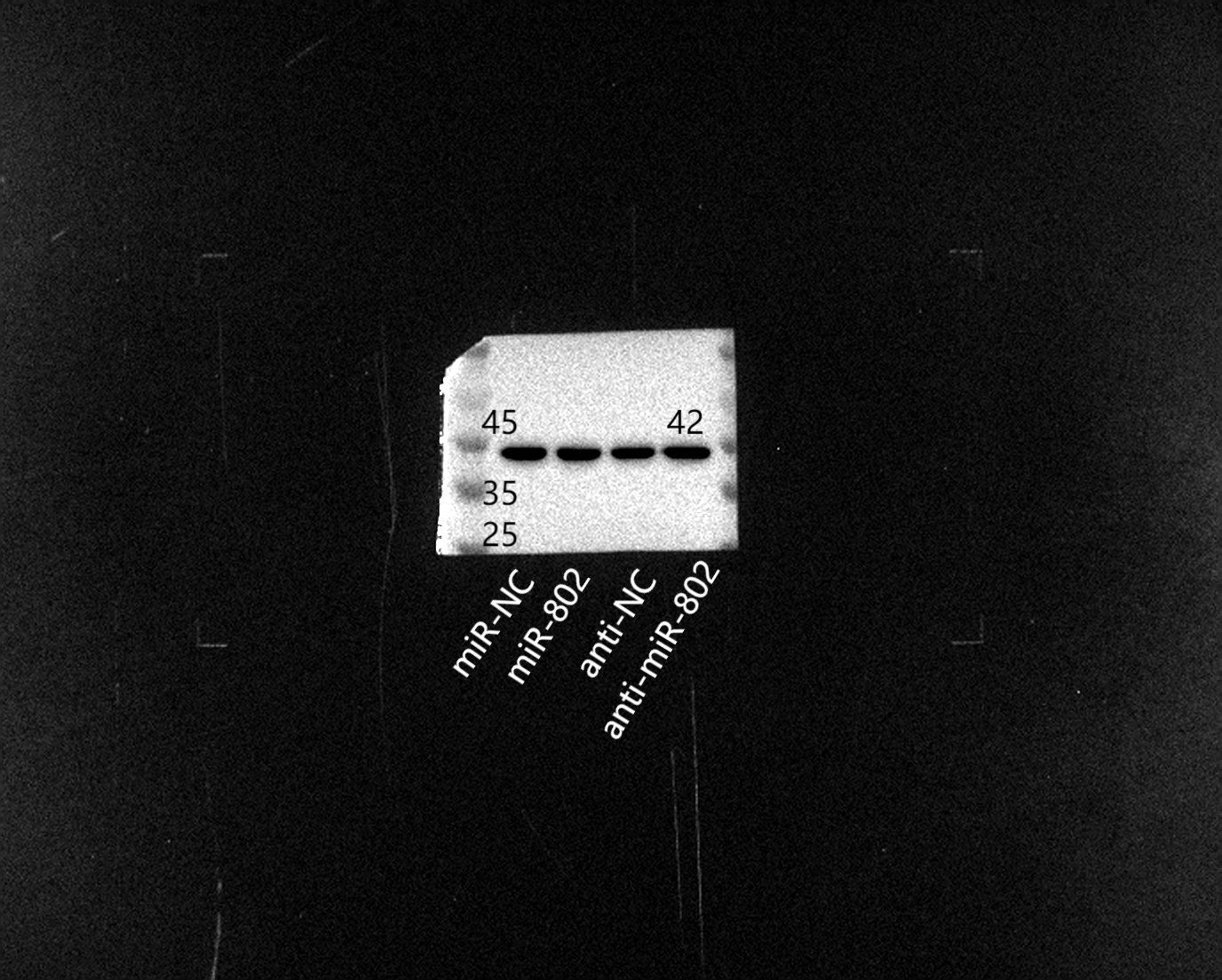

Supplement: Figure 6—source data 3. — The original files of the full raw unedited blots of p100/p52 and β-Actin in 3T3-L1 cells transfected with Mir802 mimics or Mir802 inhibitor. [file elife-99162-fig6-data3.zip › tubulin.png]

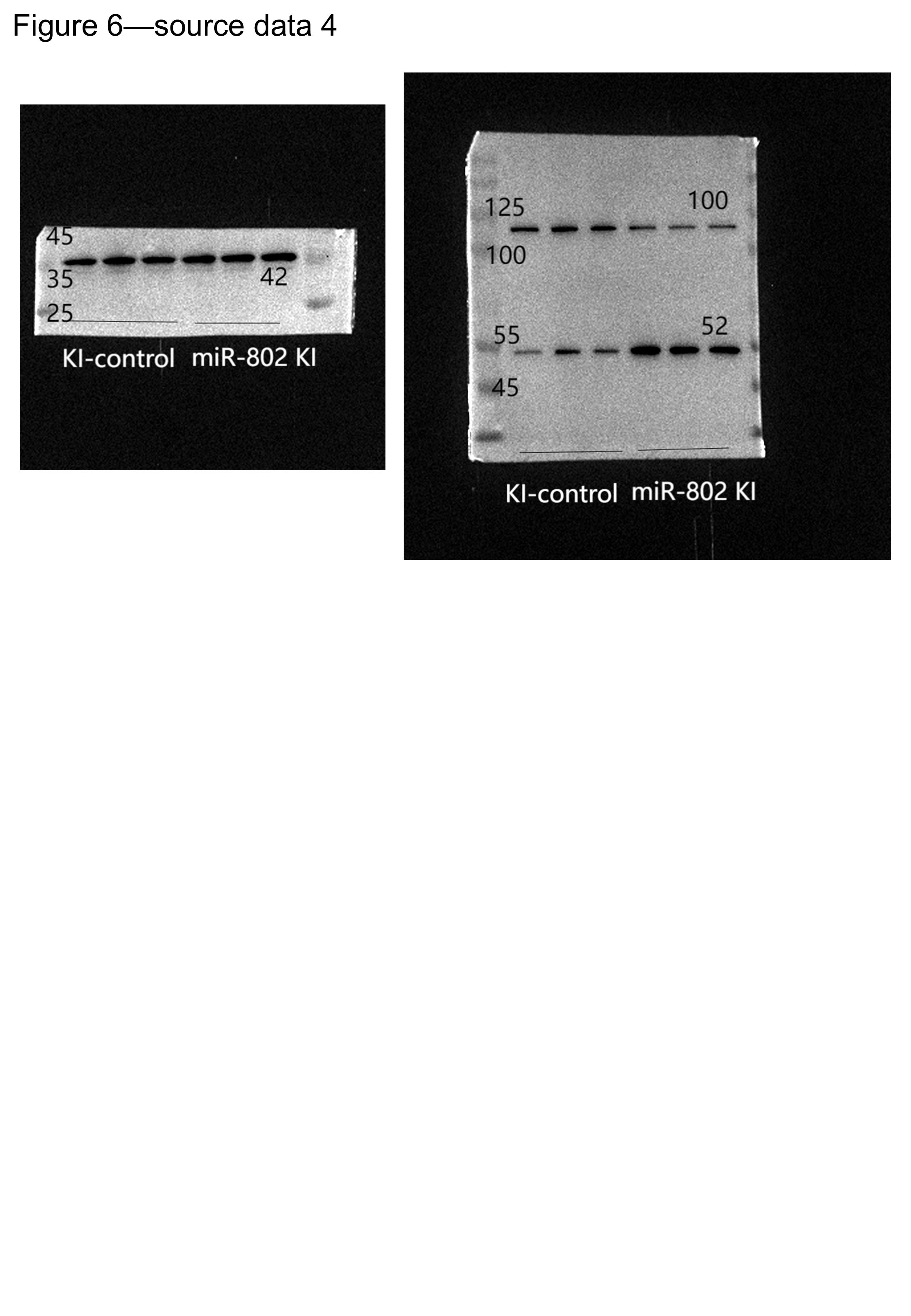

Supplement: Figure 6—source data 4. — The original files of the full raw unedited blots of p100/p52 and β-Actin in the epiWAT of Mir802 KI mice (n=3). [file elife-99162-fig6-data4.zip › Figure 6ΓÇösource data 4.tif]

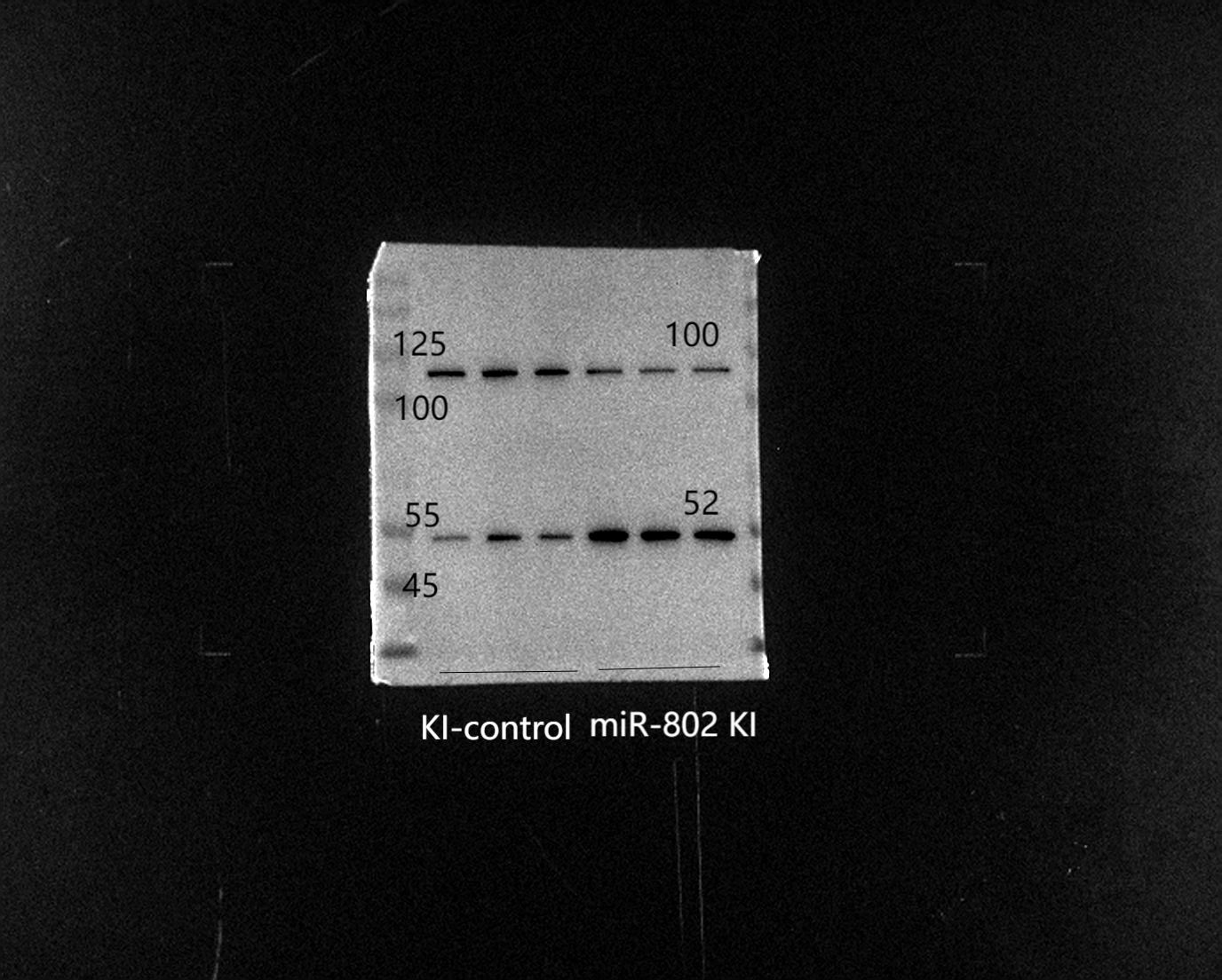

Supplement: Figure 6—source data 4. — The original files of the full raw unedited blots of p100/p52 and β-Actin in the epiWAT of Mir802 KI mice (n=3). [file elife-99162-fig6-data4.zip › p100-p52.png]

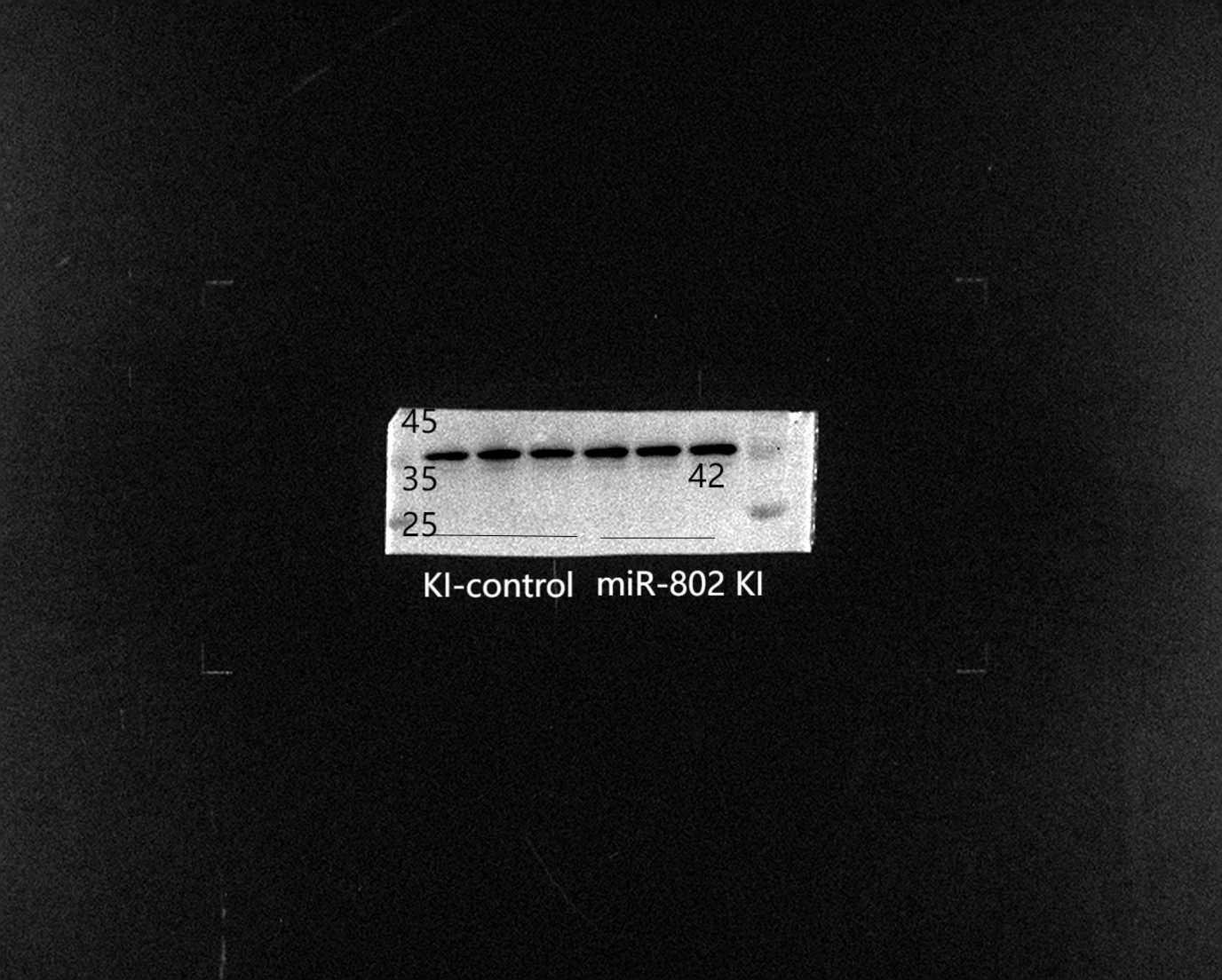

Supplement: Figure 6—source data 4. — The original files of the full raw unedited blots of p100/p52 and β-Actin in the epiWAT of Mir802 KI mice (n=3). [file elife-99162-fig6-data4.zip › ╬▓-Actin.png]

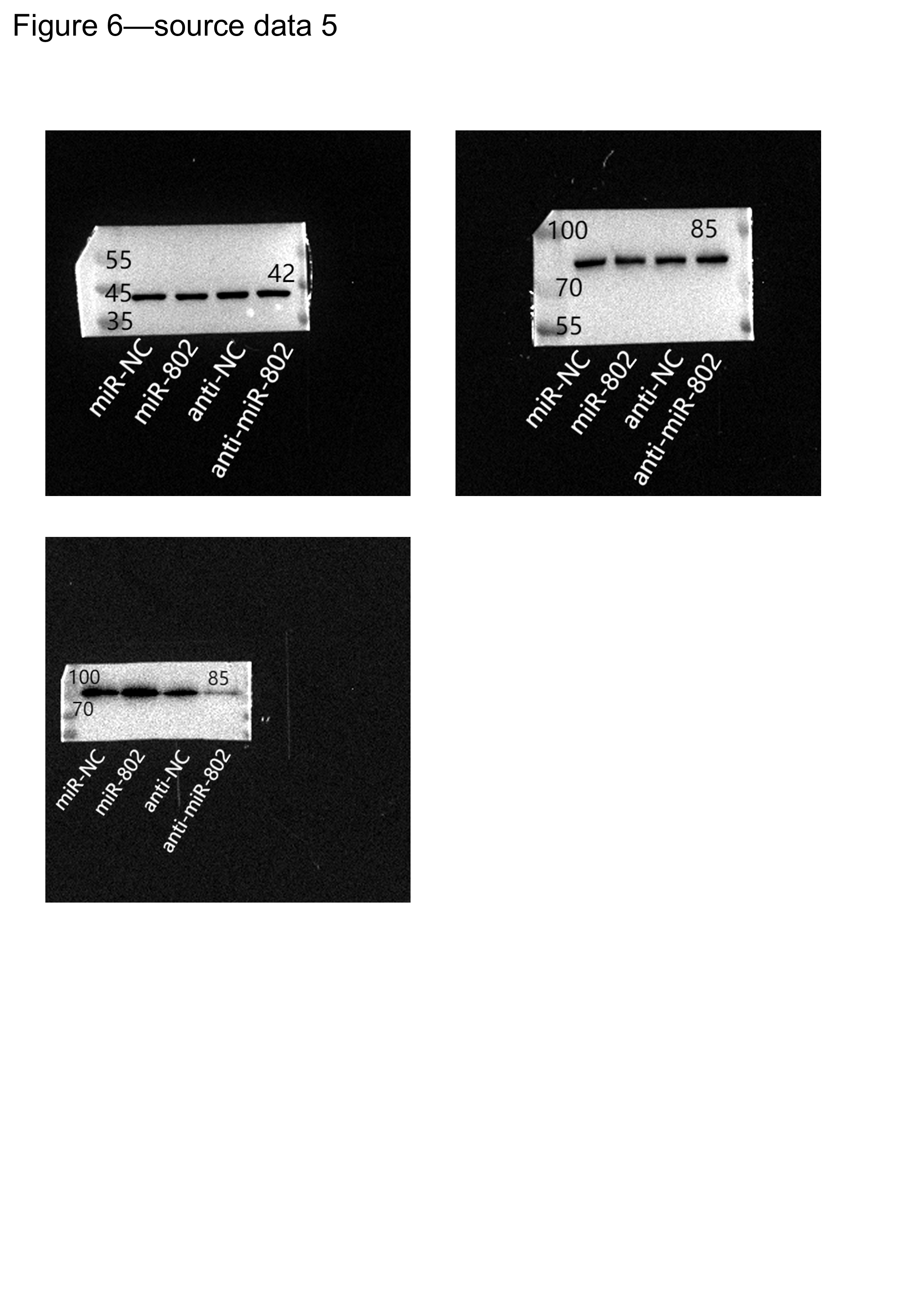

Supplement: Figure 6—source data 5. — The original files of the full raw unedited blots of P-IKK-α, IKK-α, and β-Actin in 3T3-L1 cells transfected with Mir802 mimics or Mir802 inhibitor. [file elife-99162-fig6-data5.zip › Figure 6ΓÇösource data 5.tif]

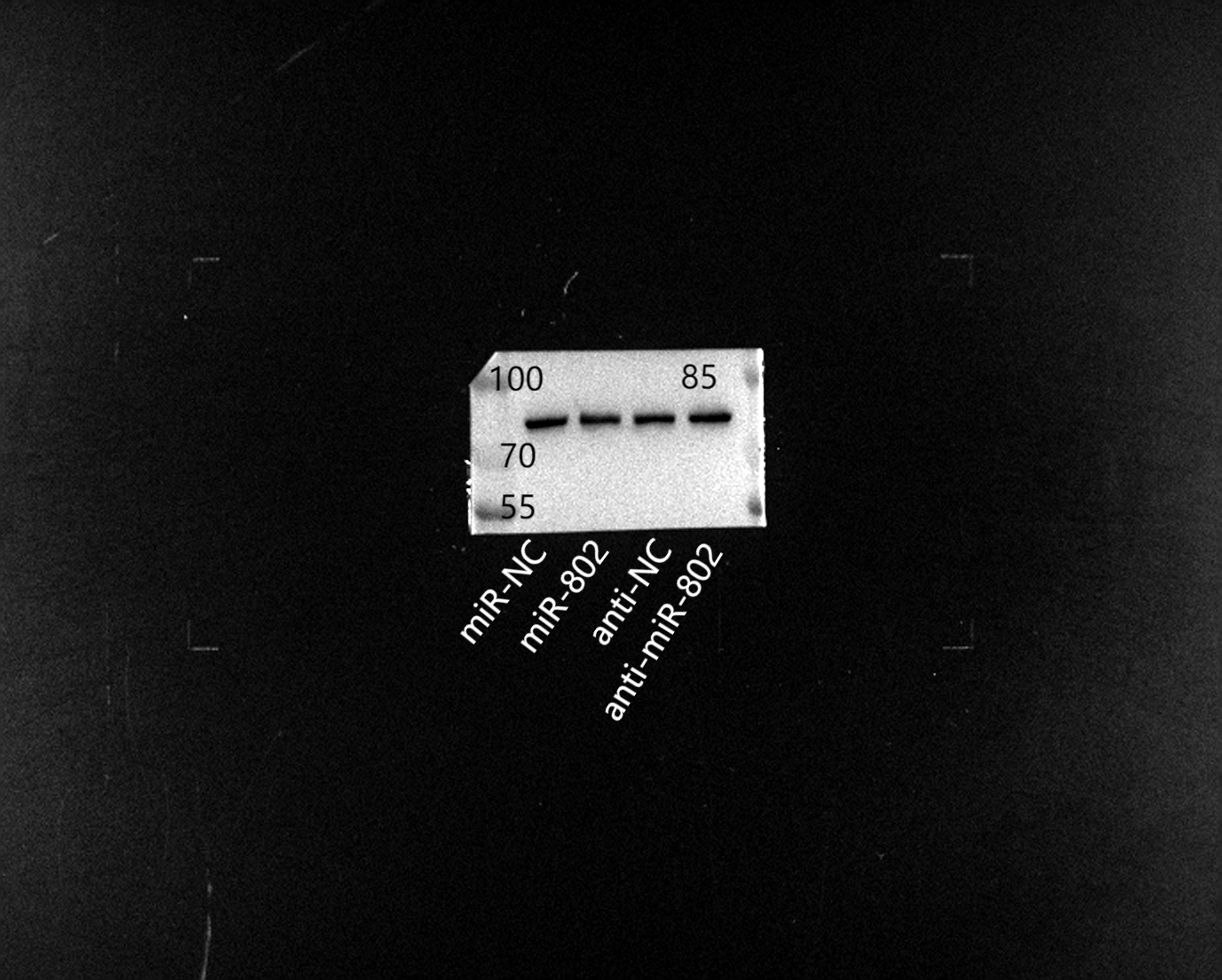

Supplement: Figure 6—source data 5. — The original files of the full raw unedited blots of P-IKK-α, IKK-α, and β-Actin in 3T3-L1 cells transfected with Mir802 mimics or Mir802 inhibitor. [file elife-99162-fig6-data5.zip › IKK.png]

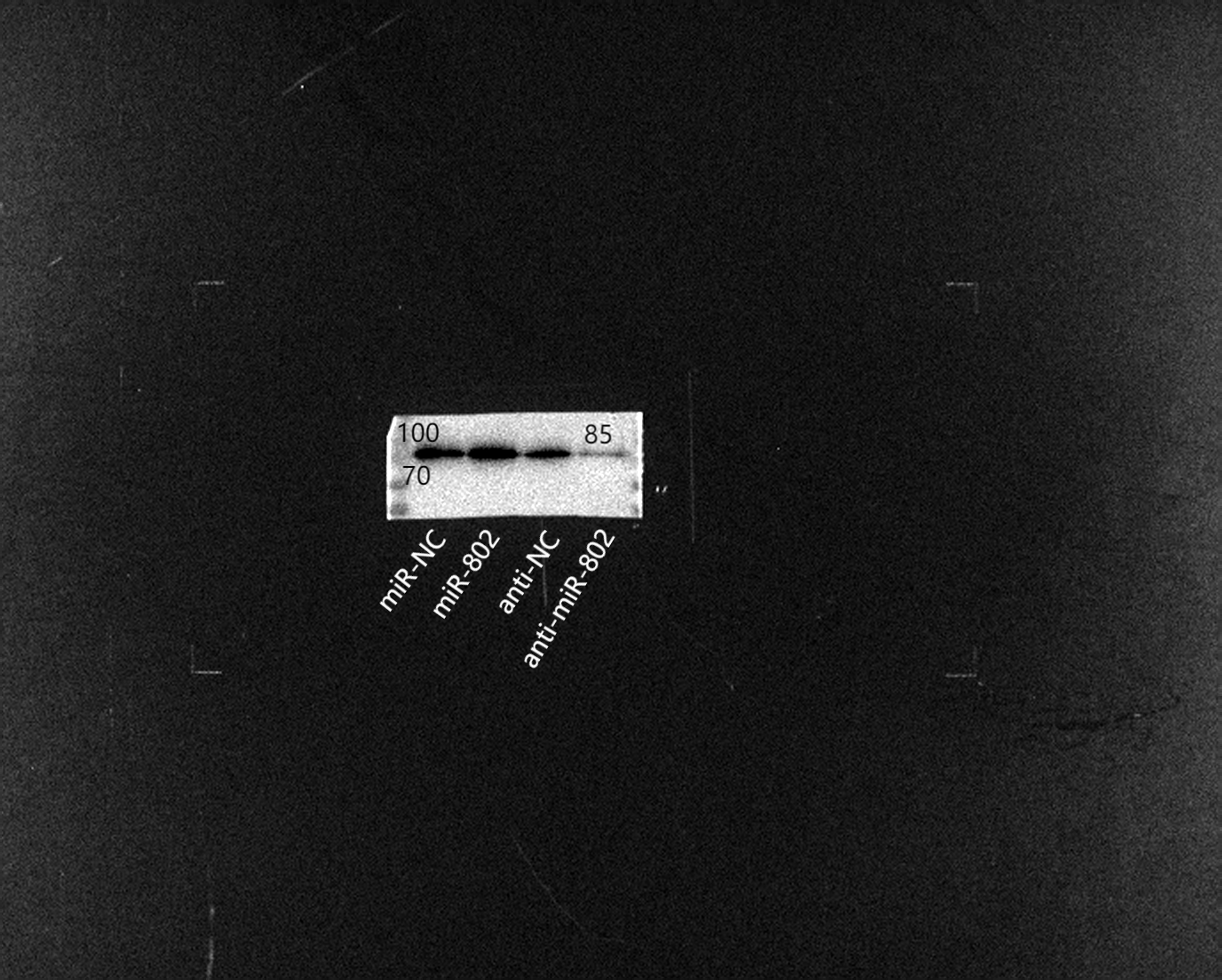

Supplement: Figure 6—source data 5. — The original files of the full raw unedited blots of P-IKK-α, IKK-α, and β-Actin in 3T3-L1 cells transfected with Mir802 mimics or Mir802 inhibitor. [file elife-99162-fig6-data5.zip › P-IKK-╬▒.png]

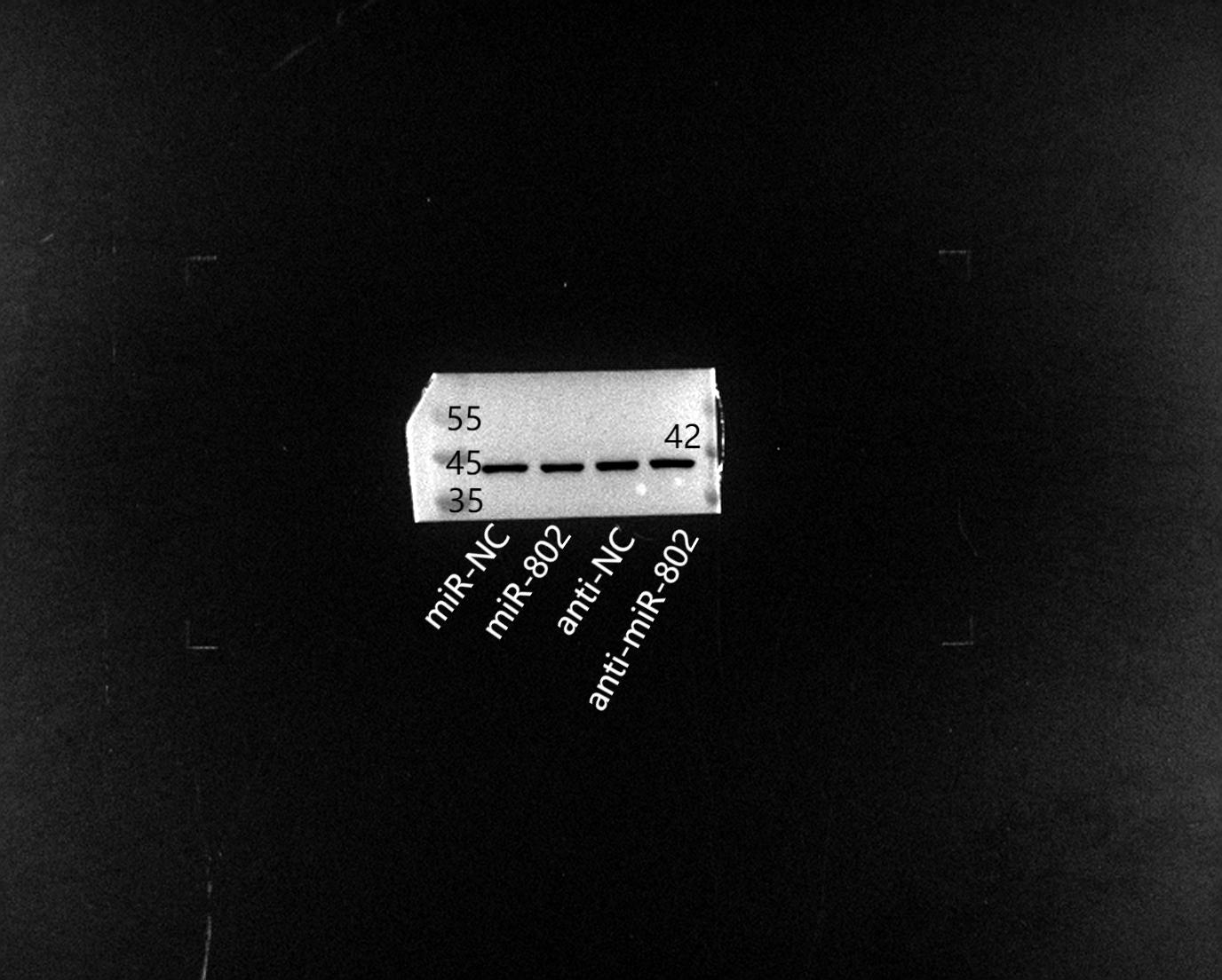

Supplement: Figure 6—source data 5. — The original files of the full raw unedited blots of P-IKK-α, IKK-α, and β-Actin in 3T3-L1 cells transfected with Mir802 mimics or Mir802 inhibitor. [file elife-99162-fig6-data5.zip › ╬▓-Actin.png]

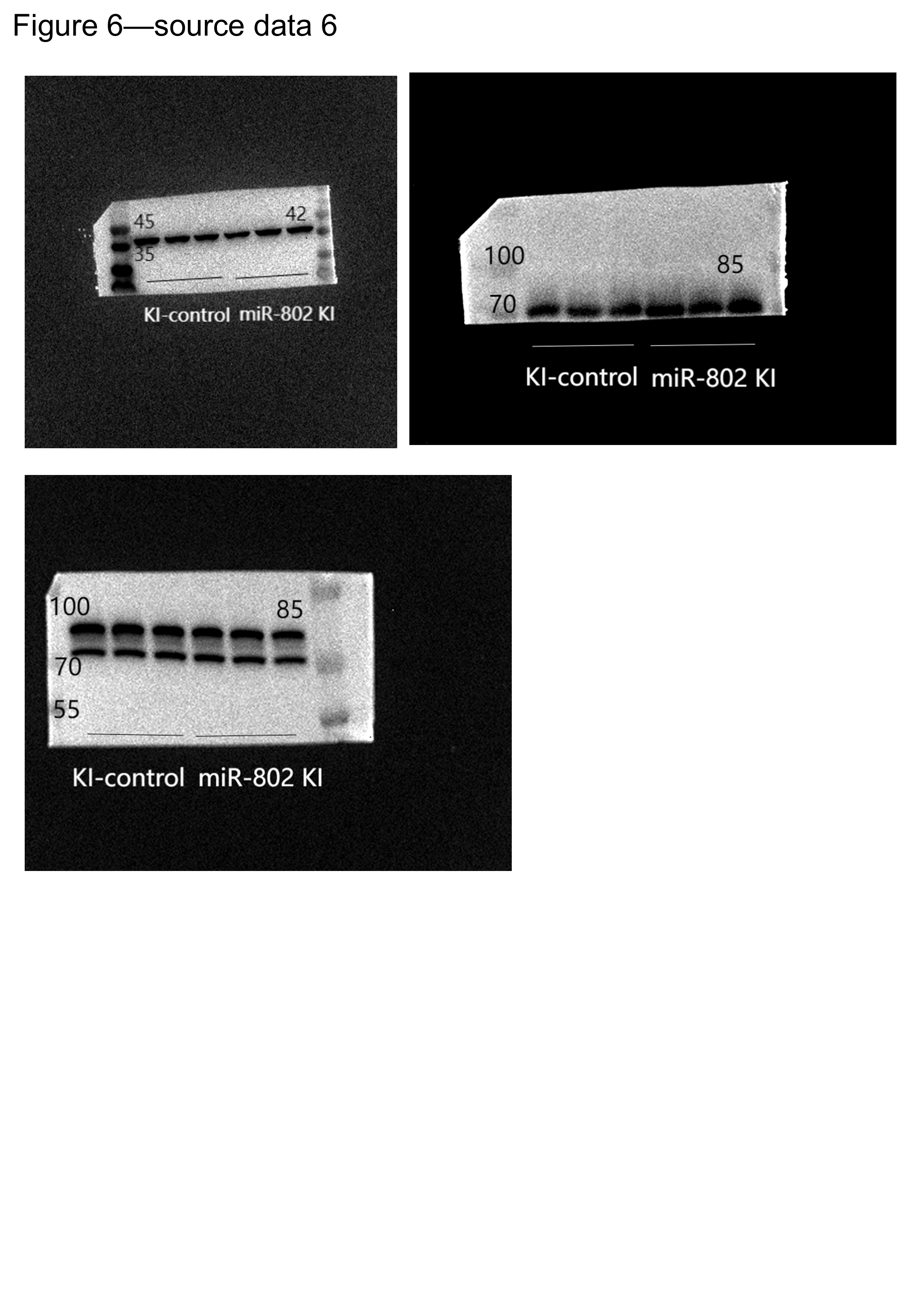

Supplement: Figure 6—source data 6. — The original files of the full raw unedited blots of P-IKK-α, IKK-α, and β-Actin in the epiWAT of Mir802 KI mice (n=3). [file elife-99162-fig6-data6.zip › Figure 6ΓÇösource data 6.tif]

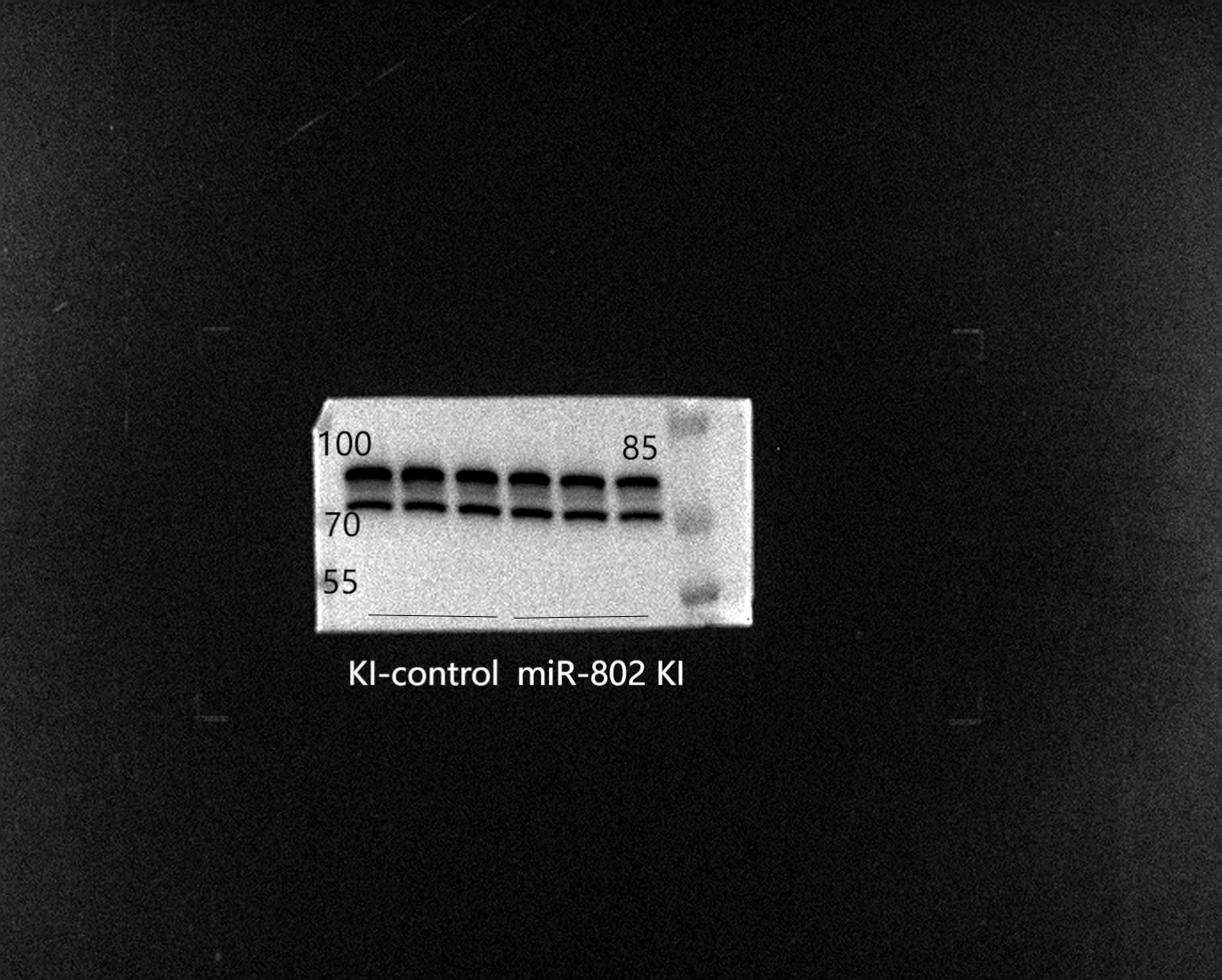

Supplement: Figure 6—source data 6. — The original files of the full raw unedited blots of P-IKK-α, IKK-α, and β-Actin in the epiWAT of Mir802 KI mice (n=3). [file elife-99162-fig6-data6.zip › IKK-╬▒.png]

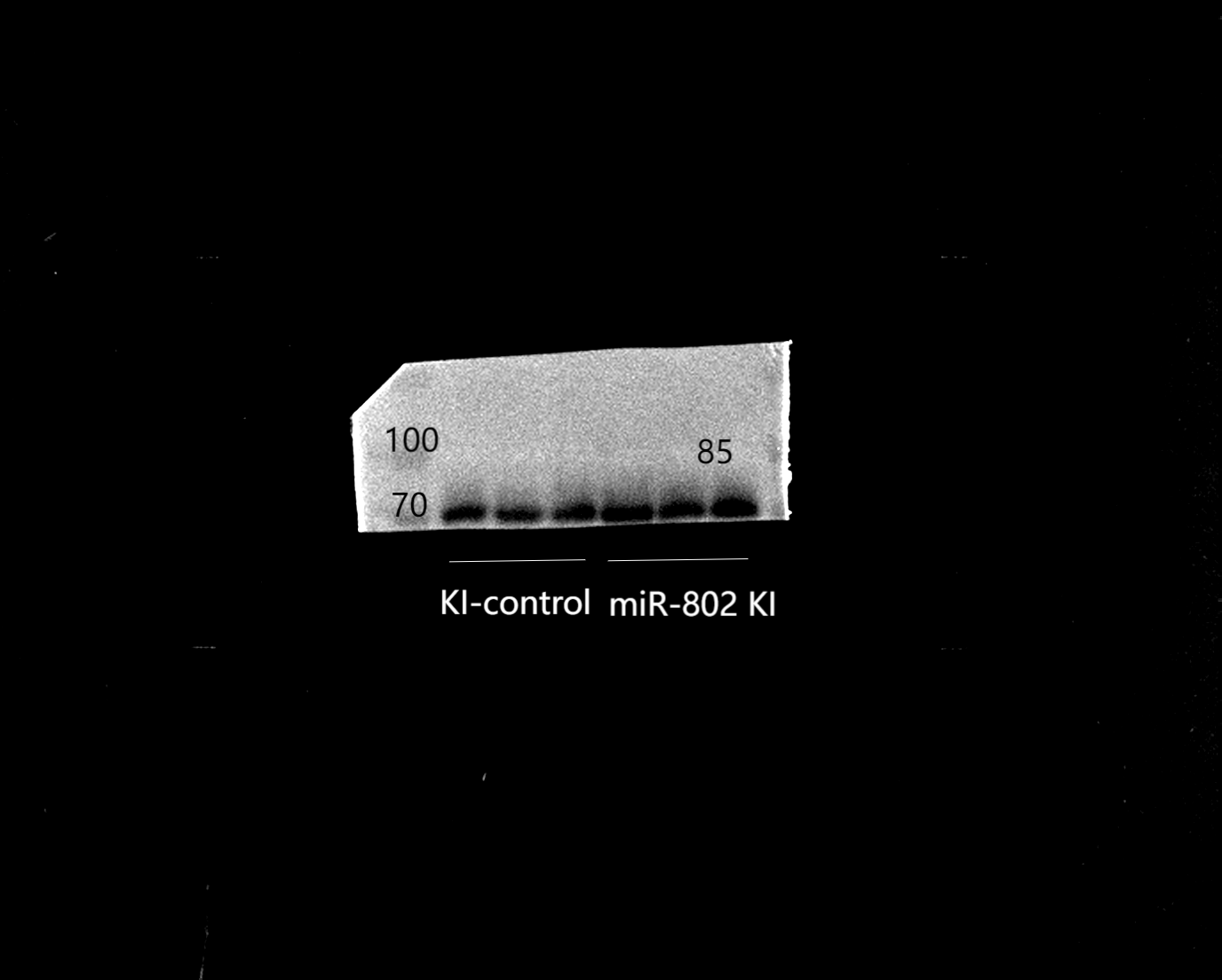

Supplement: Figure 6—source data 6. — The original files of the full raw unedited blots of P-IKK-α, IKK-α, and β-Actin in the epiWAT of Mir802 KI mice (n=3). [file elife-99162-fig6-data6.zip › P-IKK-╬▒.png]

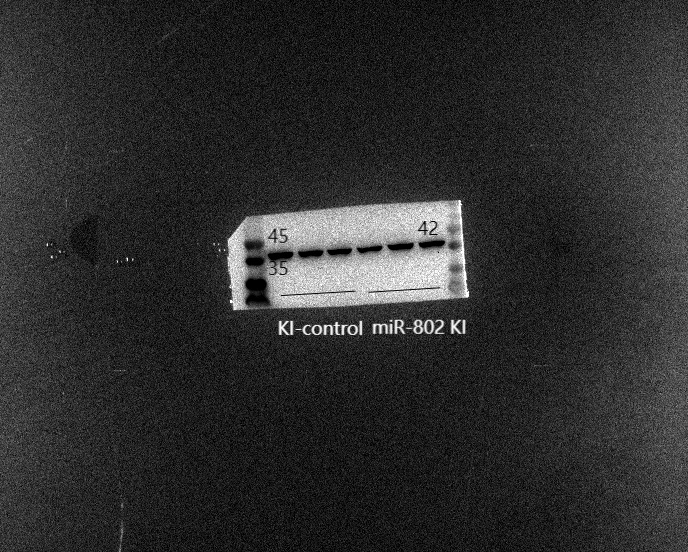

Supplement: Figure 6—source data 6. — The original files of the full raw unedited blots of P-IKK-α, IKK-α, and β-Actin in the epiWAT of Mir802 KI mice (n=3). [file elife-99162-fig6-data6.zip › ╬▓-Actin.png]

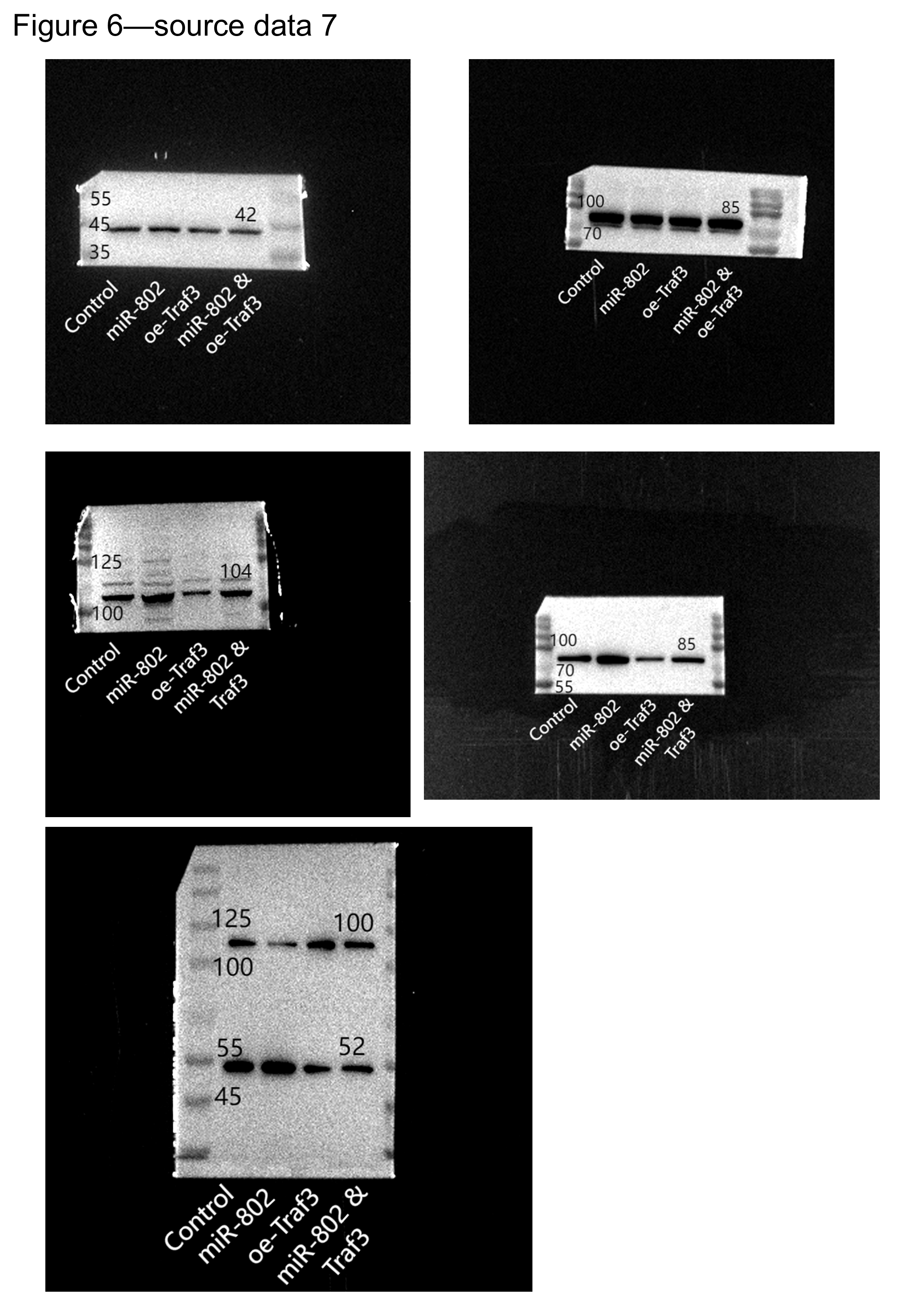

Supplement: Figure 6—source data 7. — The original files of the full raw unedited blots of p100/p52, P-IKK-α, IKK-α, NIK, and β-Actin in the 3T3-L1 cells. [file elife-99162-fig6-data7.zip › Figure 6ΓÇösource data 7.tif]

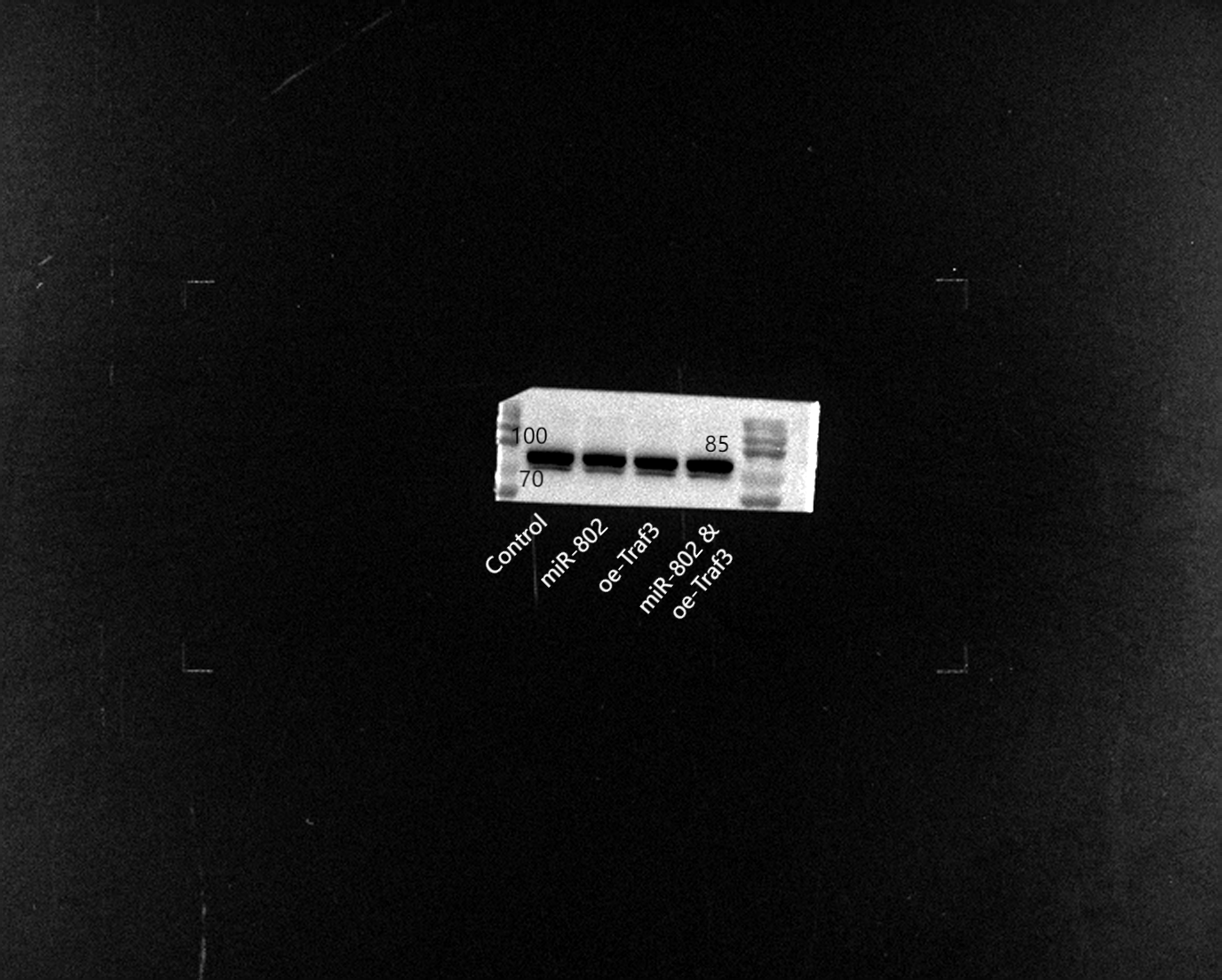

Supplement: Figure 6—source data 7. — The original files of the full raw unedited blots of p100/p52, P-IKK-α, IKK-α, NIK, and β-Actin in the 3T3-L1 cells. [file elife-99162-fig6-data7.zip › IKK-╬▒.png]

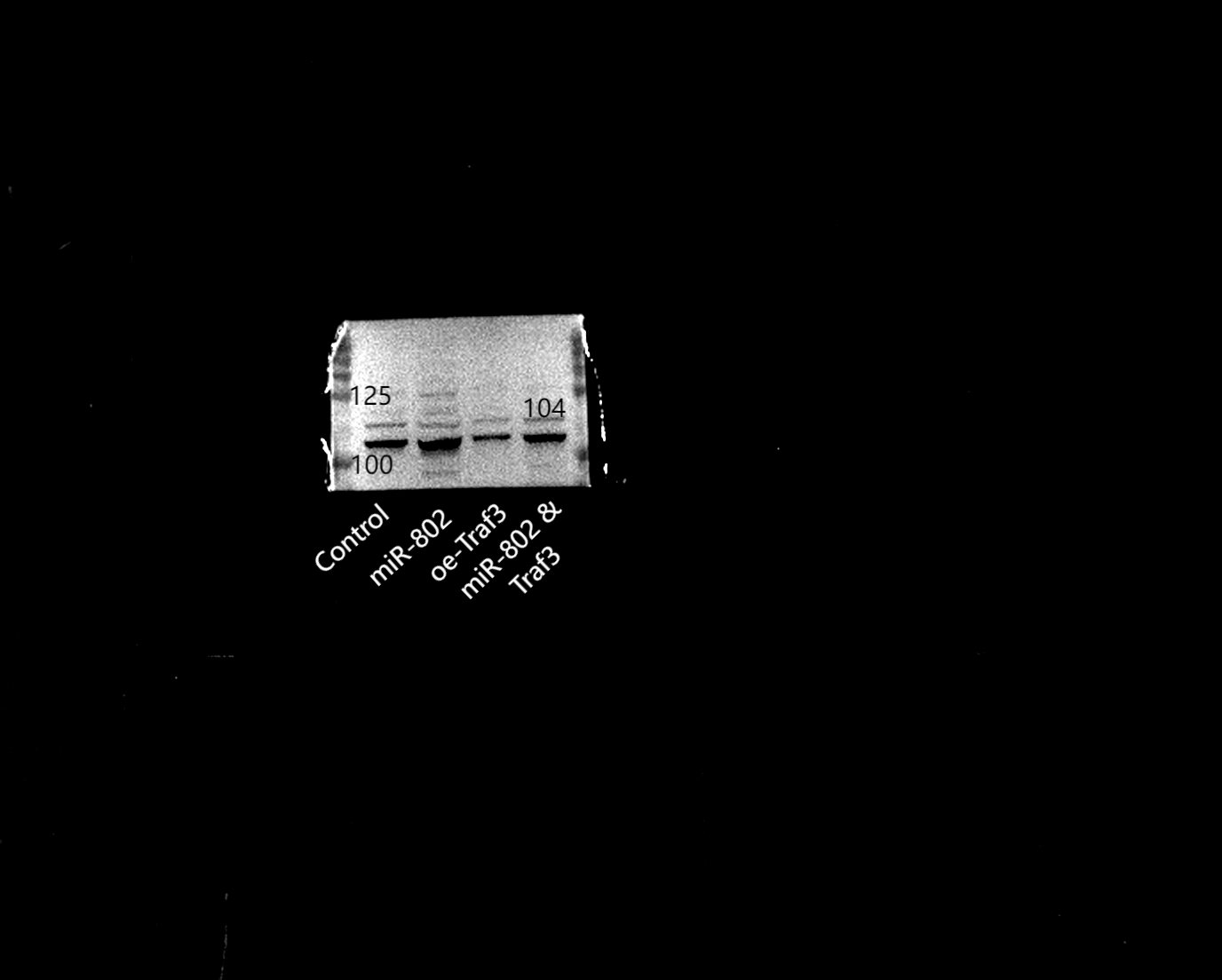

Supplement: Figure 6—source data 7. — The original files of the full raw unedited blots of p100/p52, P-IKK-α, IKK-α, NIK, and β-Actin in the 3T3-L1 cells. [file elife-99162-fig6-data7.zip › NIK.png]

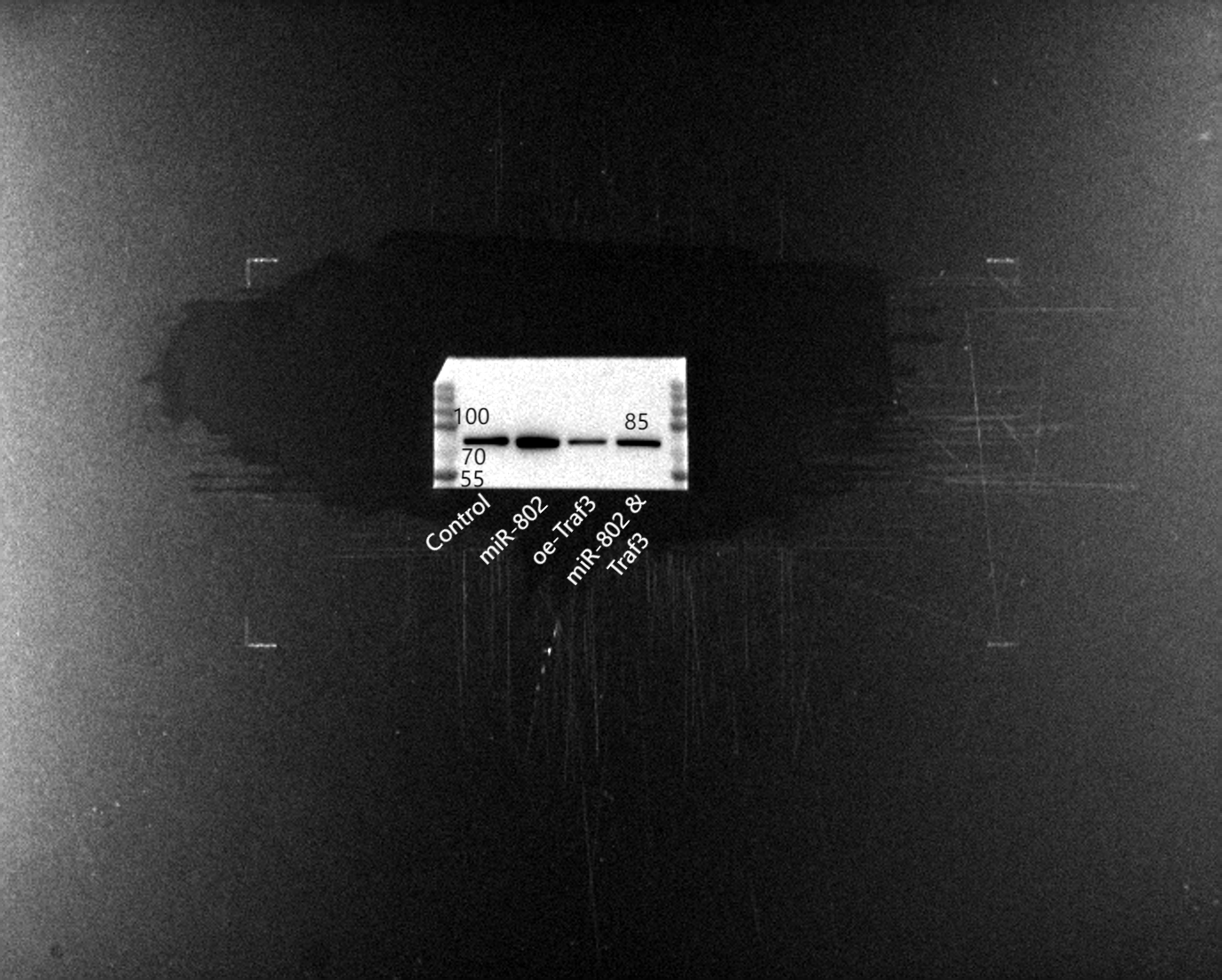

Supplement: Figure 6—source data 7. — The original files of the full raw unedited blots of p100/p52, P-IKK-α, IKK-α, NIK, and β-Actin in the 3T3-L1 cells. [file elife-99162-fig6-data7.zip › P-IKK-╬▒.png]

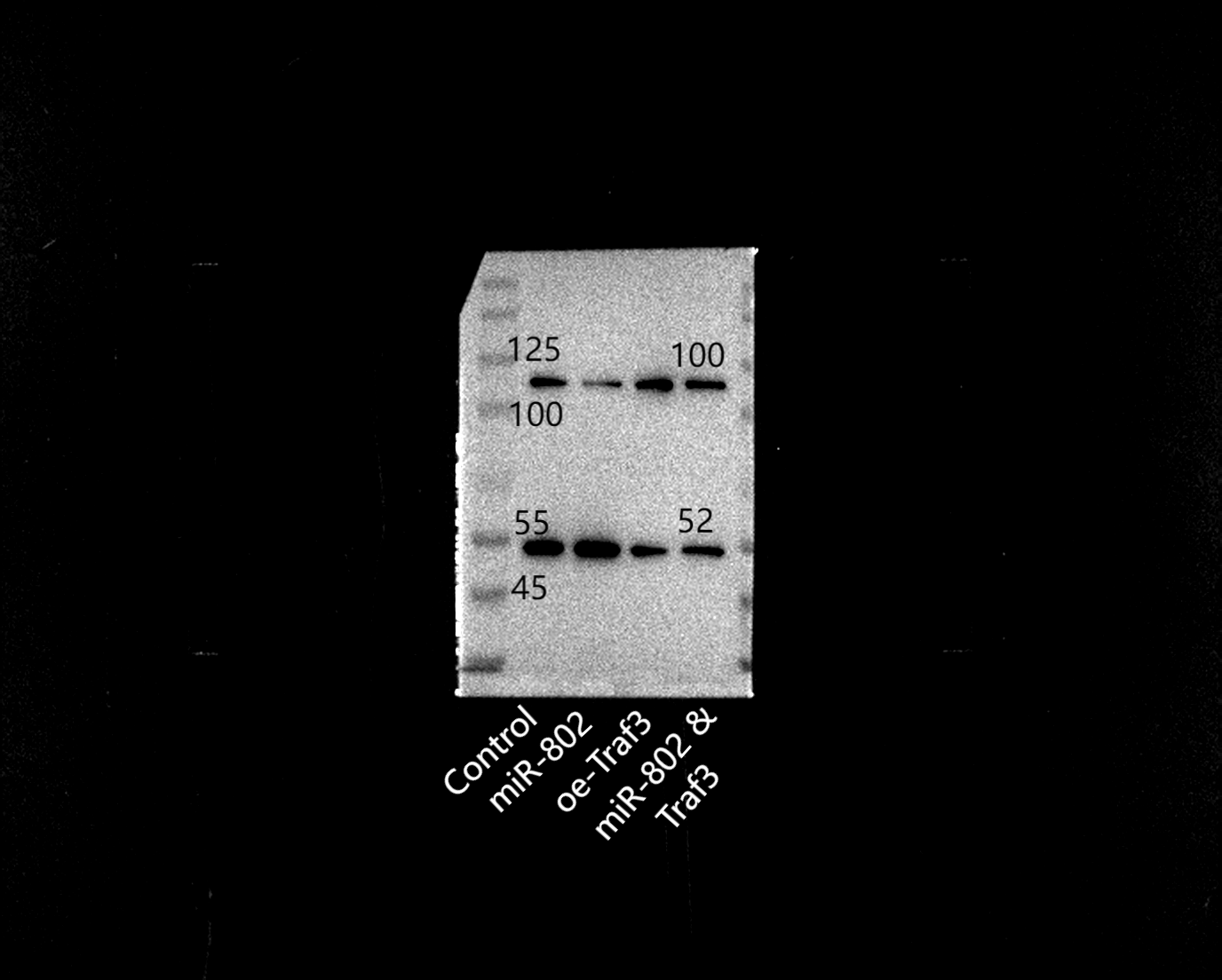

Supplement: Figure 6—source data 7. — The original files of the full raw unedited blots of p100/p52, P-IKK-α, IKK-α, NIK, and β-Actin in the 3T3-L1 cells. [file elife-99162-fig6-data7.zip › p100-p52.png]

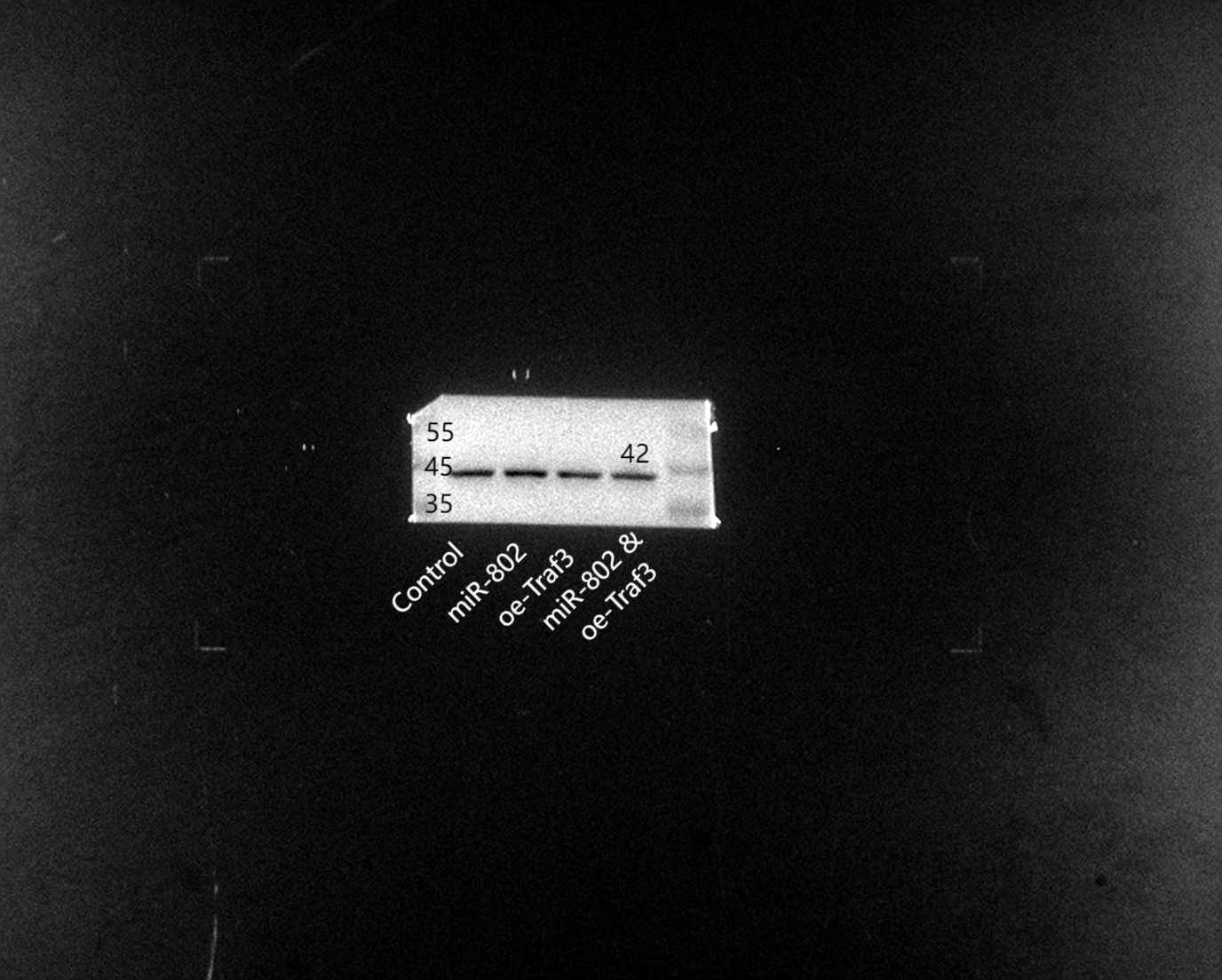

Supplement: Figure 6—source data 7. — The original files of the full raw unedited blots of p100/p52, P-IKK-α, IKK-α, NIK, and β-Actin in the 3T3-L1 cells. [file elife-99162-fig6-data7.zip › ╬▓-Actin.png]

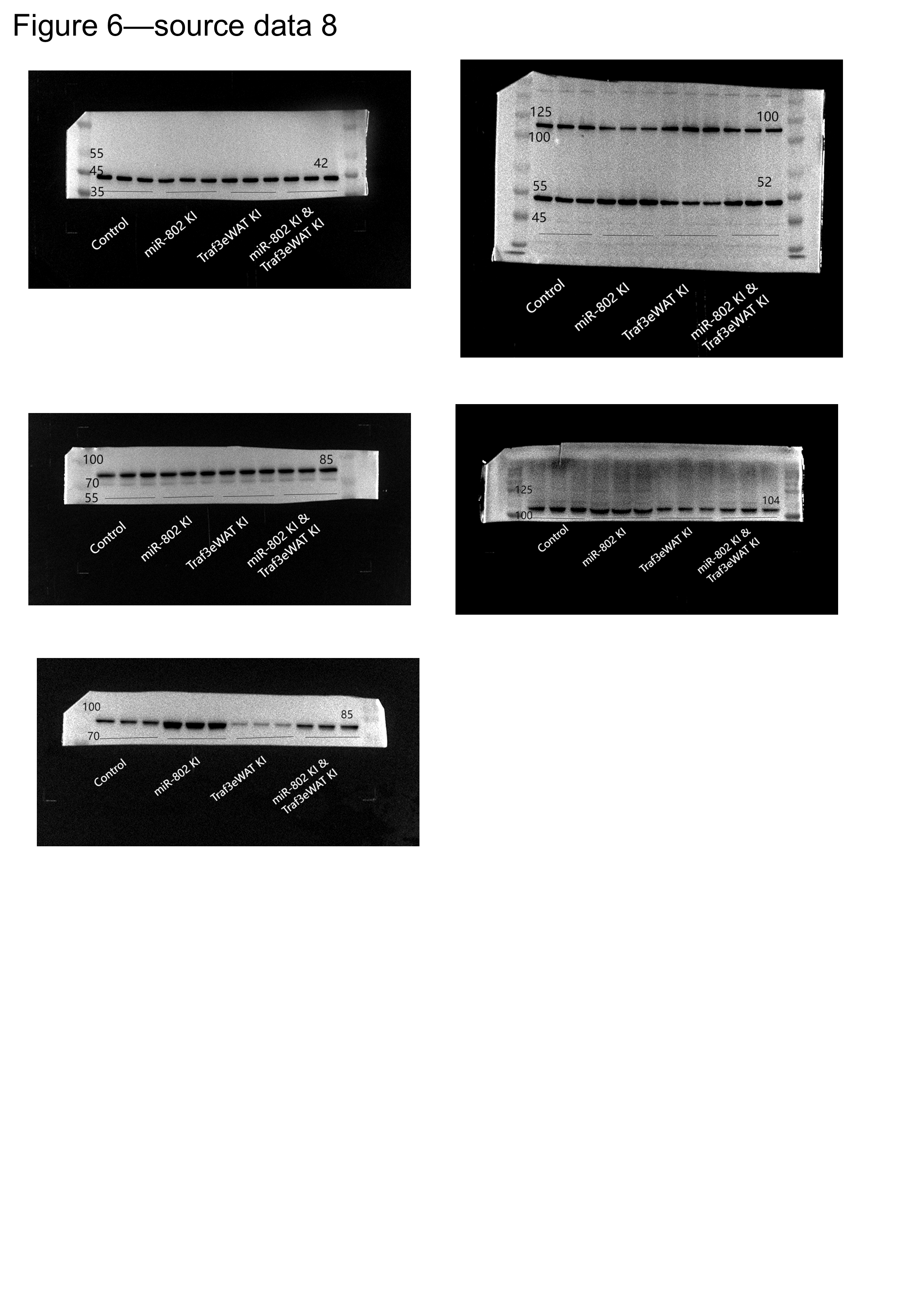

Supplement: Figure 6—source data 8. — The original files of the full raw unedited blots of p100/p52, P-IKK-α, IKK-α, NIK, and β-Actin in the epiWAT of Mir802 KI and Traf3 eWAT OE mice (n=3). [file elife-99162-fig6-data8.zip › Figure 6ΓÇösource data 8.tif]

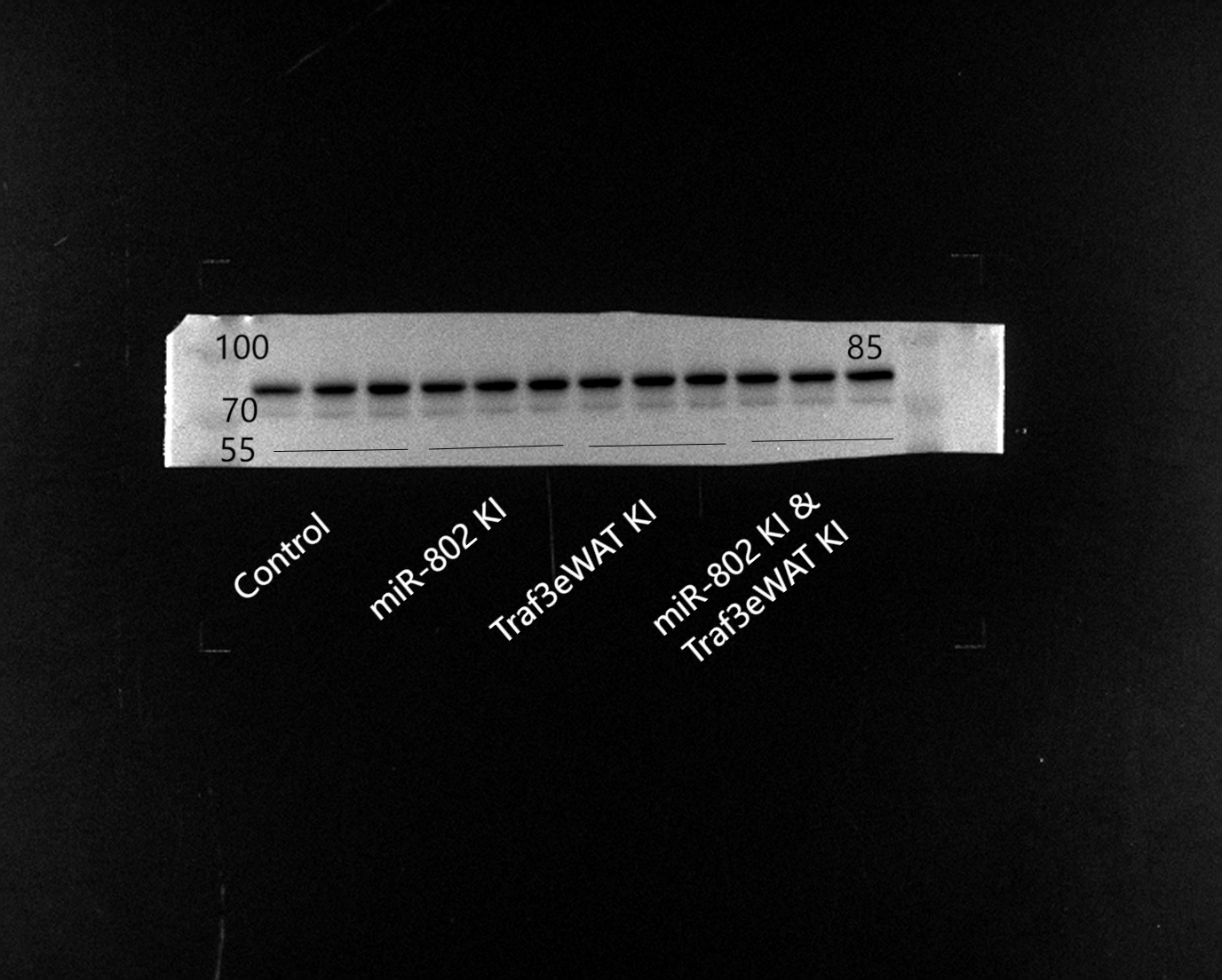

Supplement: Figure 6—source data 8. — The original files of the full raw unedited blots of p100/p52, P-IKK-α, IKK-α, NIK, and β-Actin in the epiWAT of Mir802 KI and Traf3 eWAT OE mice (n=3). [file elife-99162-fig6-data8.zip › IKK-╬▒.png]

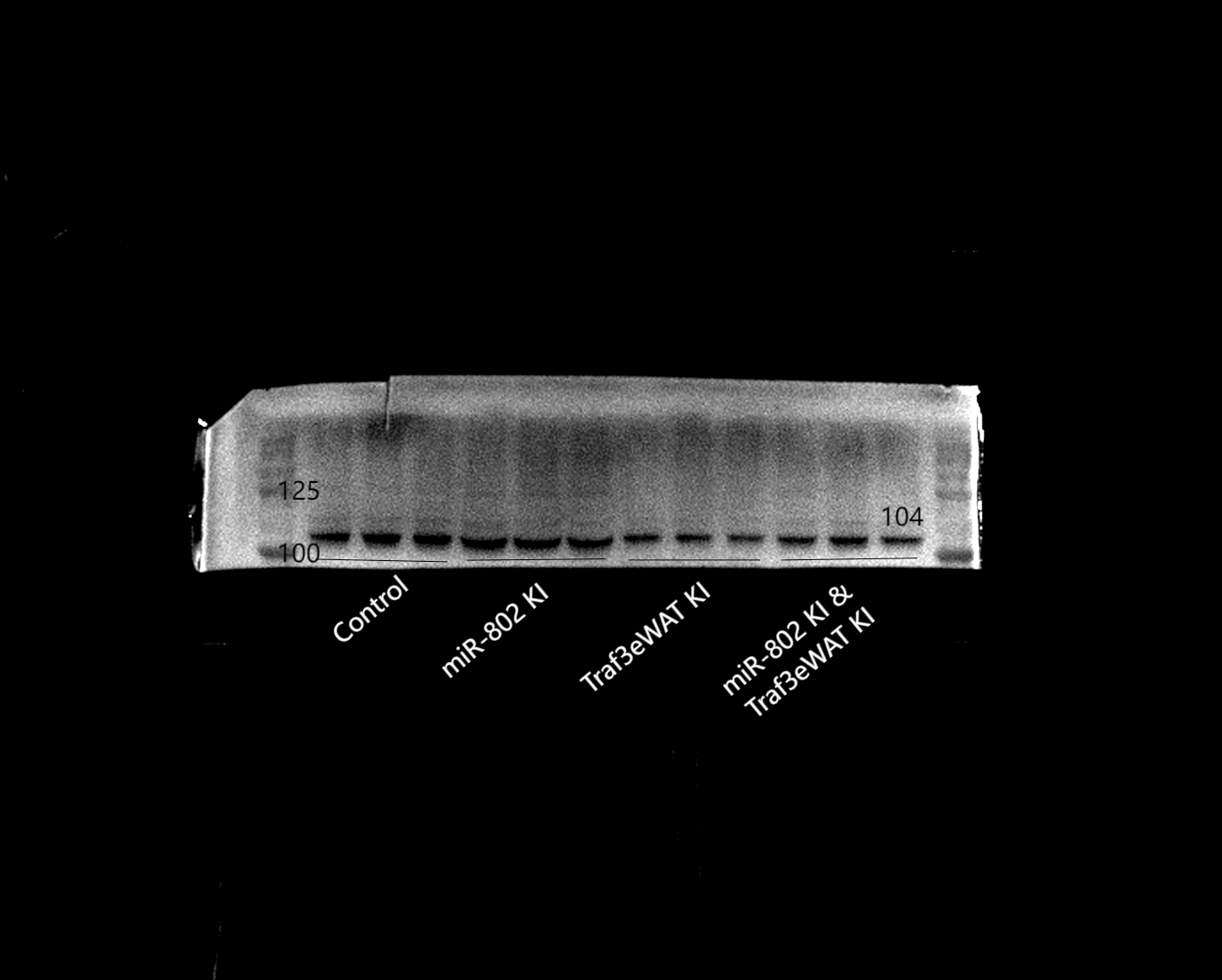

Supplement: Figure 6—source data 8. — The original files of the full raw unedited blots of p100/p52, P-IKK-α, IKK-α, NIK, and β-Actin in the epiWAT of Mir802 KI and Traf3 eWAT OE mice (n=3). [file elife-99162-fig6-data8.zip › NIK.png]

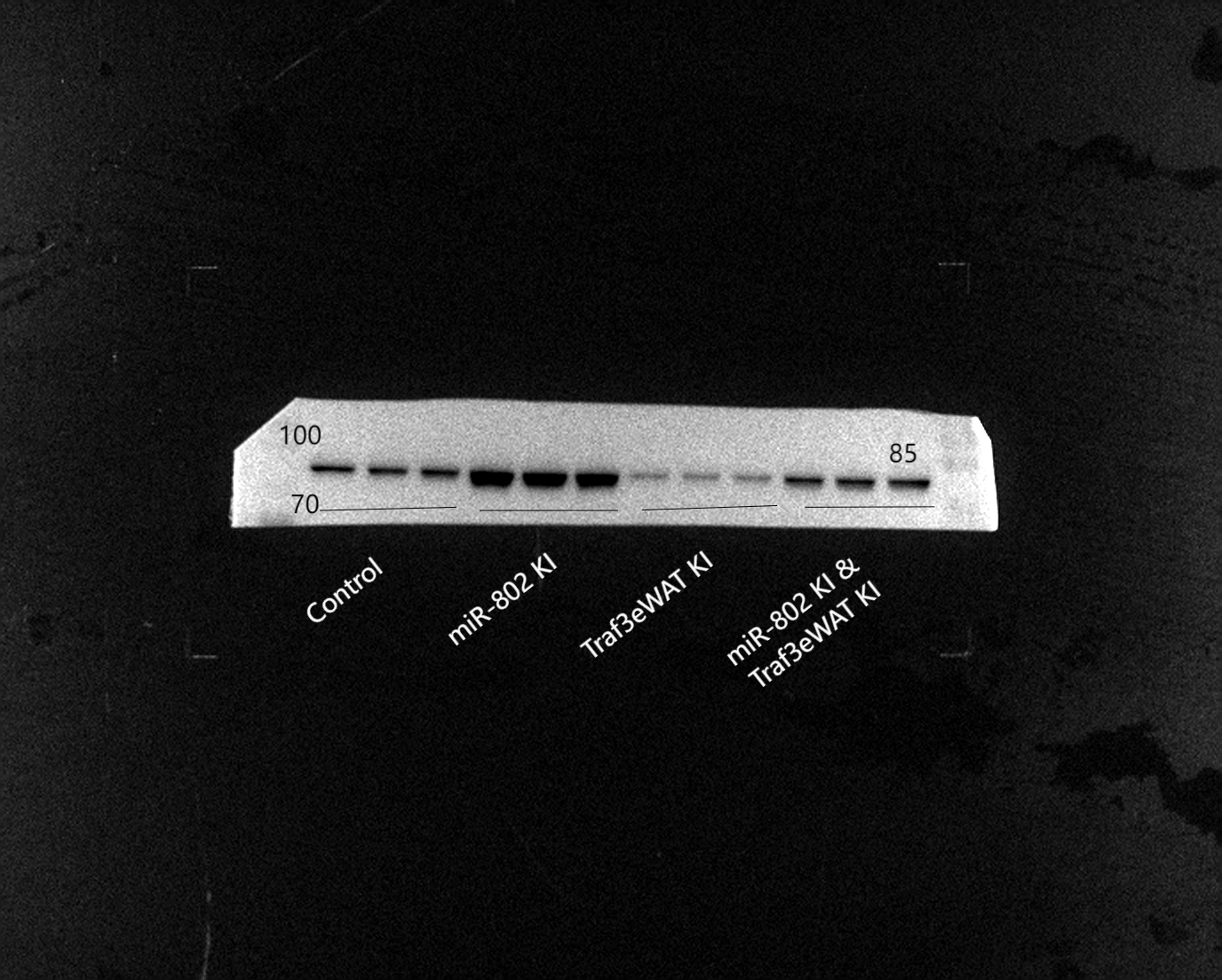

Supplement: Figure 6—source data 8. — The original files of the full raw unedited blots of p100/p52, P-IKK-α, IKK-α, NIK, and β-Actin in the epiWAT of Mir802 KI and Traf3 eWAT OE mice (n=3). [file elife-99162-fig6-data8.zip › P-IKK-╬▒.png]

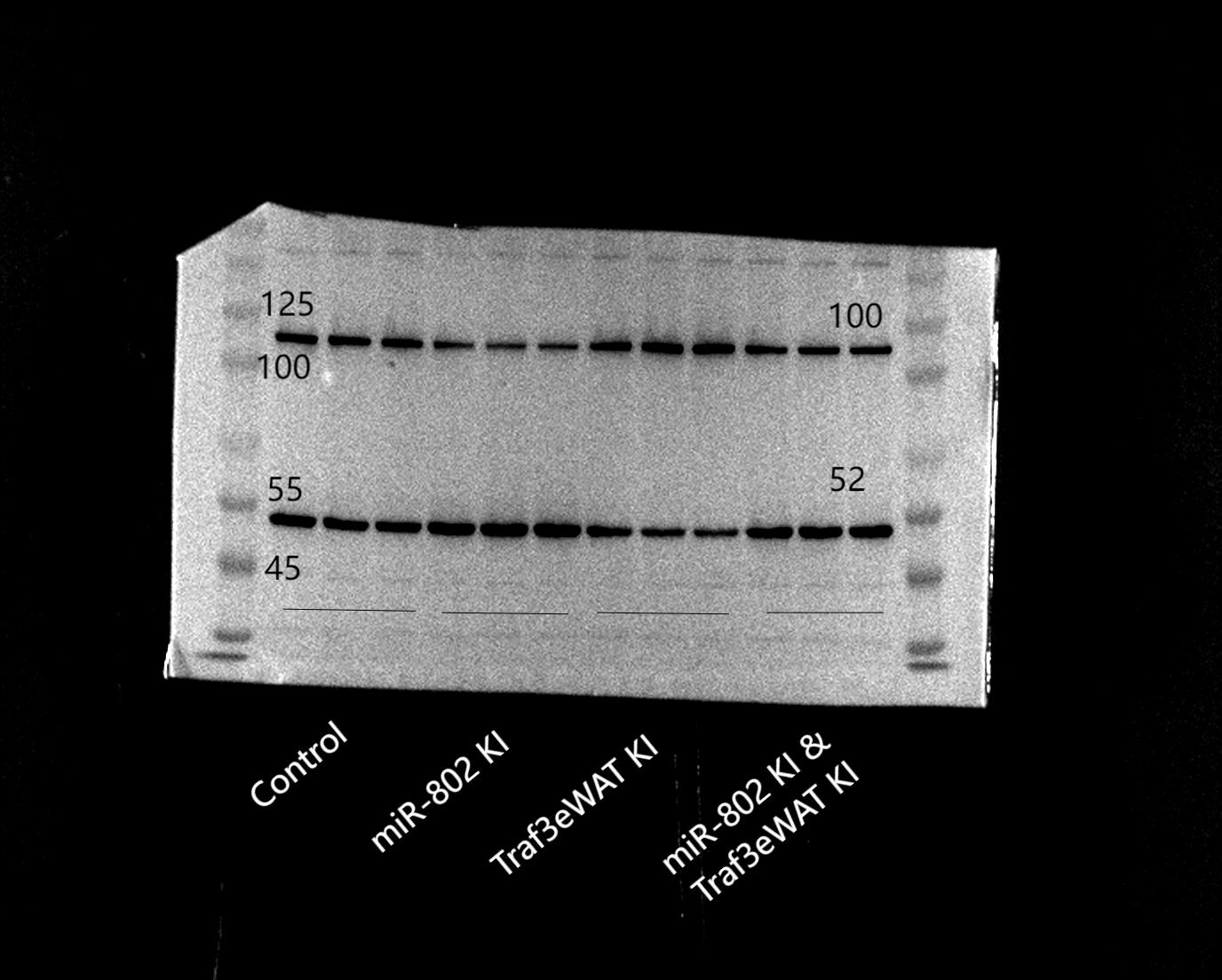

Supplement: Figure 6—source data 8. — The original files of the full raw unedited blots of p100/p52, P-IKK-α, IKK-α, NIK, and β-Actin in the epiWAT of Mir802 KI and Traf3 eWAT OE mice (n=3). [file elife-99162-fig6-data8.zip › p100-p52.png]

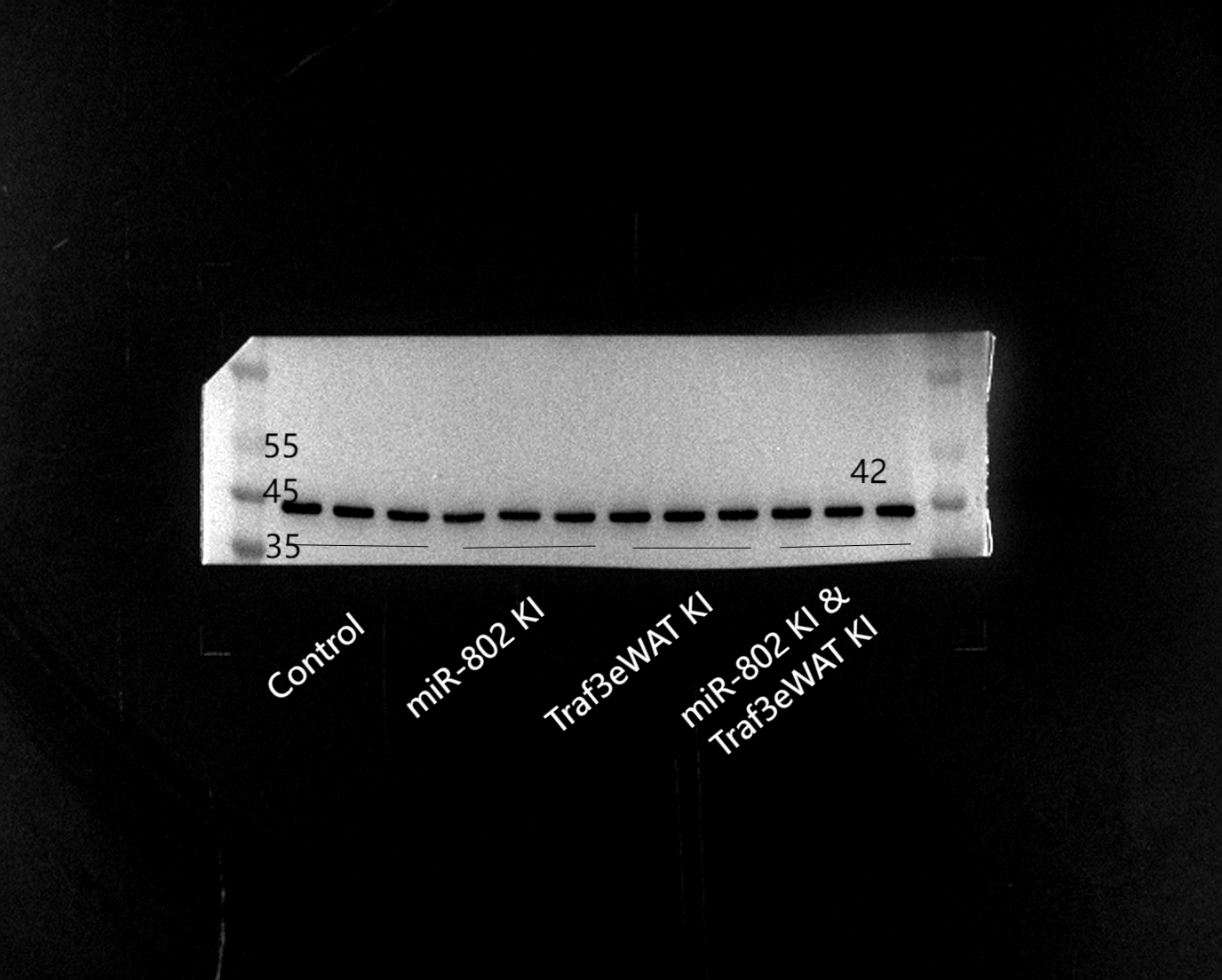

Supplement: Figure 6—source data 8. — The original files of the full raw unedited blots of p100/p52, P-IKK-α, IKK-α, NIK, and β-Actin in the epiWAT of Mir802 KI and Traf3 eWAT OE mice (n=3). [file elife-99162-fig6-data8.zip › ╬▓-Actin.png]

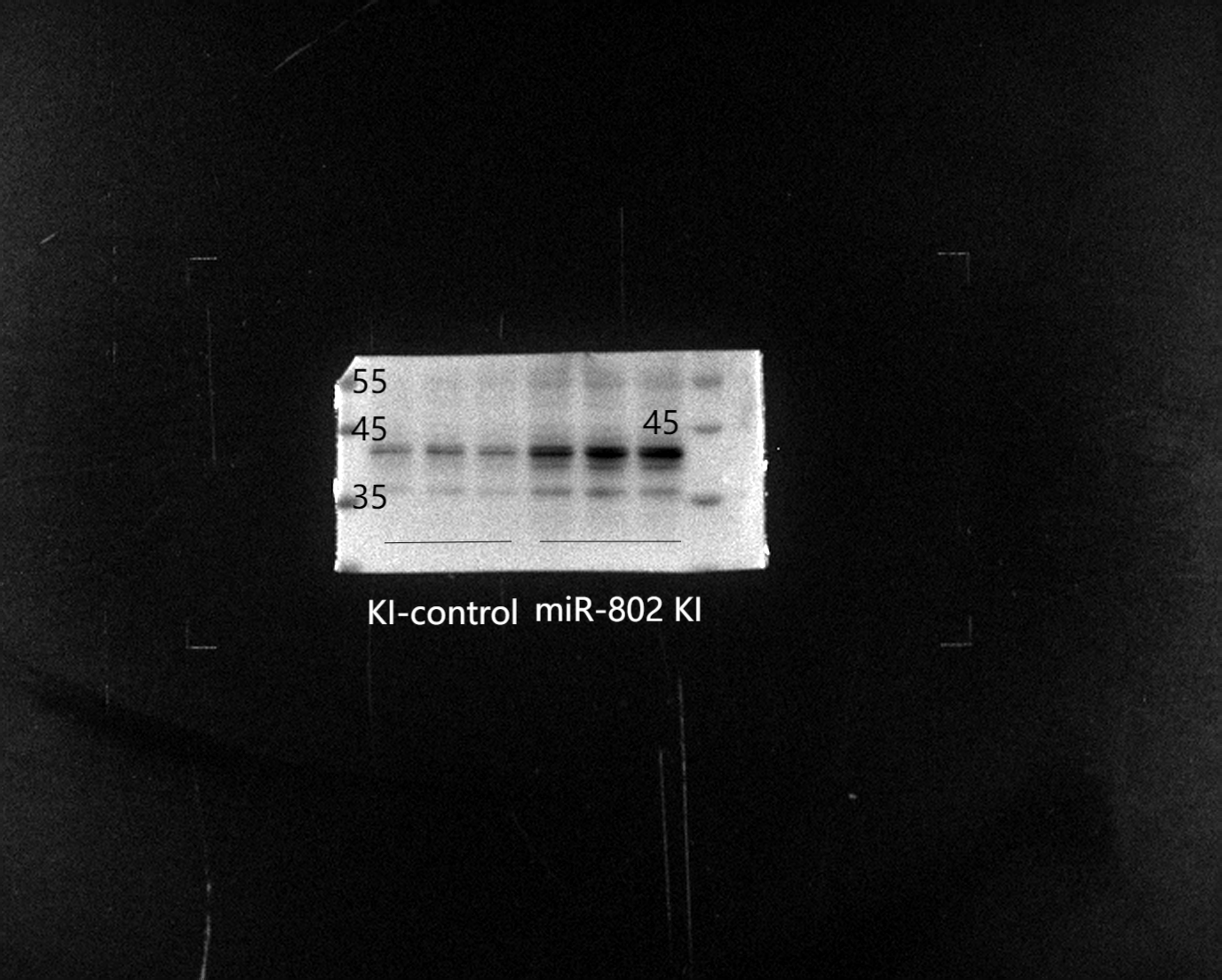

Supplement: Figure 6—source data 9. — The original files of the full raw unedited blots of some major canonical NF-κB signaling targets in the epiWAT of Mir802 KI mice(n=3). [file elife-99162-fig6-data9.zip › FasL.png]

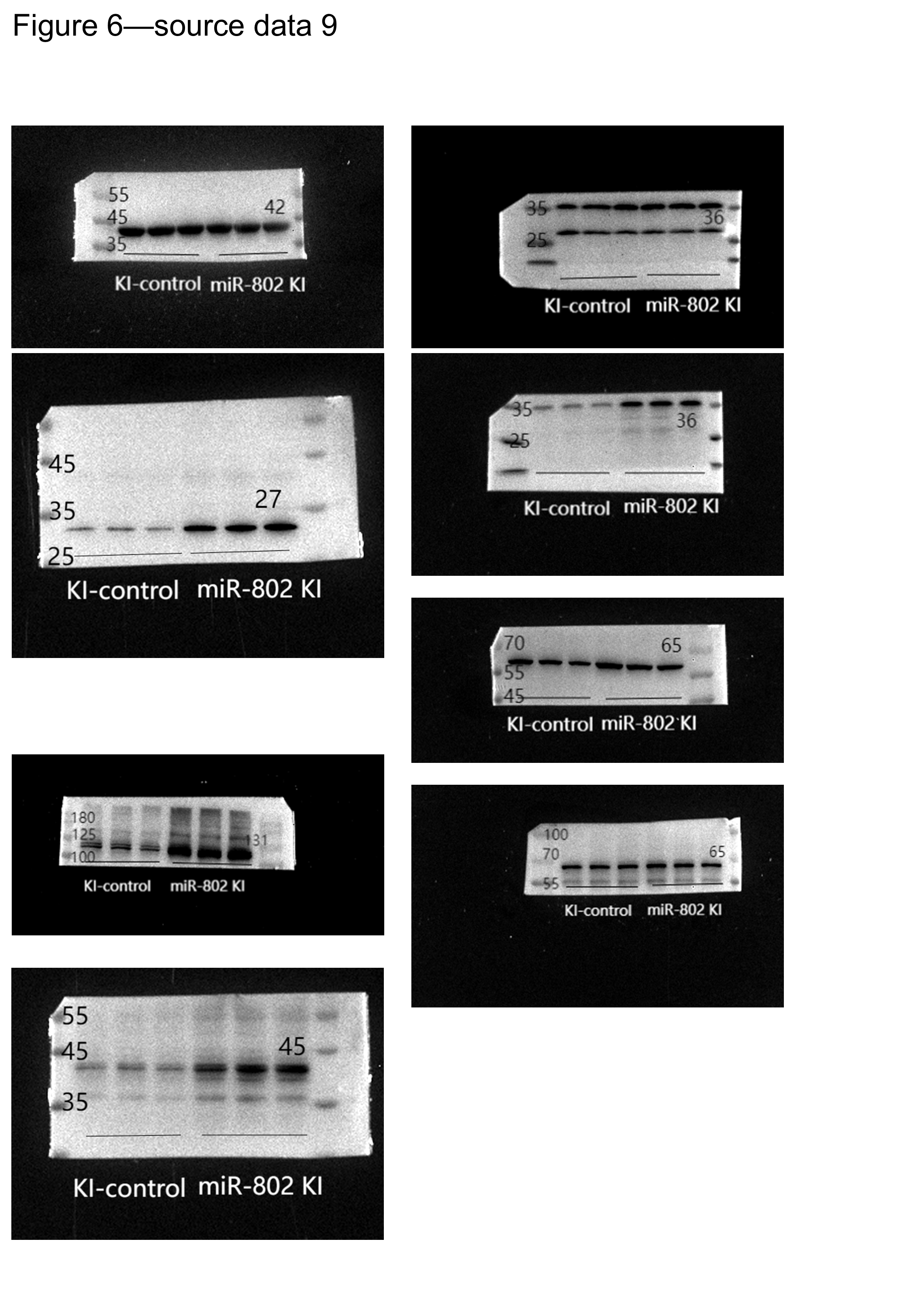

Supplement: Figure 6—source data 9. — The original files of the full raw unedited blots of some major canonical NF-κB signaling targets in the epiWAT of Mir802 KI mice(n=3). [file elife-99162-fig6-data9.zip › Figure 6ΓÇösource data 9.tif]

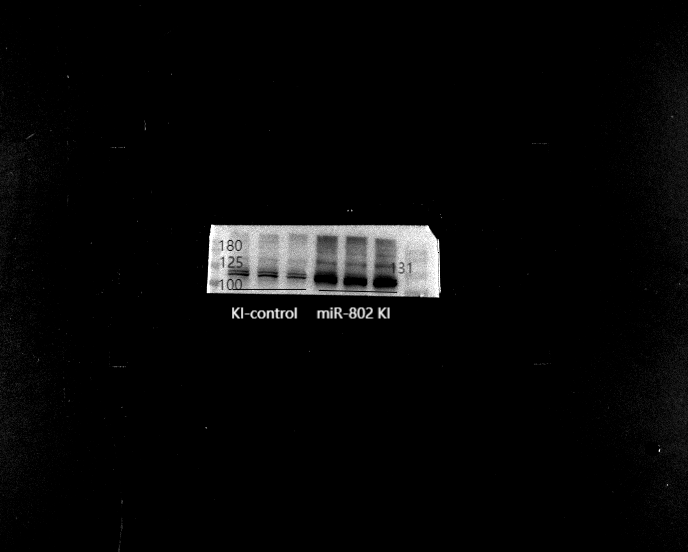

Supplement: Figure 6—source data 9. — The original files of the full raw unedited blots of some major canonical NF-κB signaling targets in the epiWAT of Mir802 KI mice(n=3). [file elife-99162-fig6-data9.zip › iNOS.png]

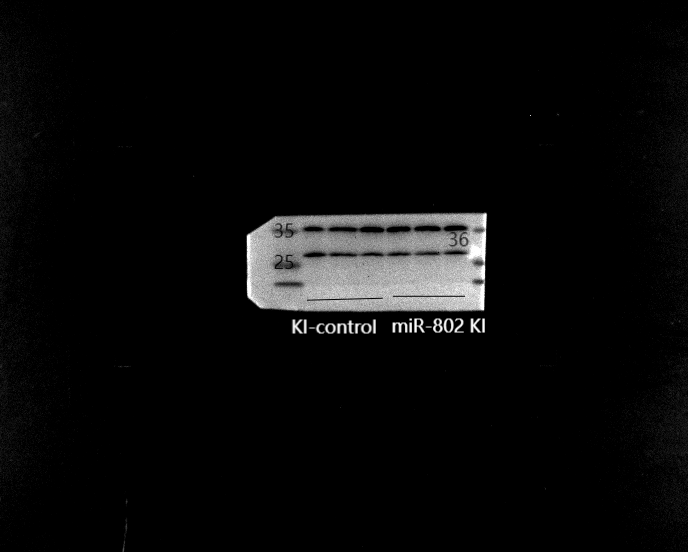

Supplement: Figure 6—source data 9. — The original files of the full raw unedited blots of some major canonical NF-κB signaling targets in the epiWAT of Mir802 KI mice(n=3). [file elife-99162-fig6-data9.zip › KI-IkB╬▒-5.png]

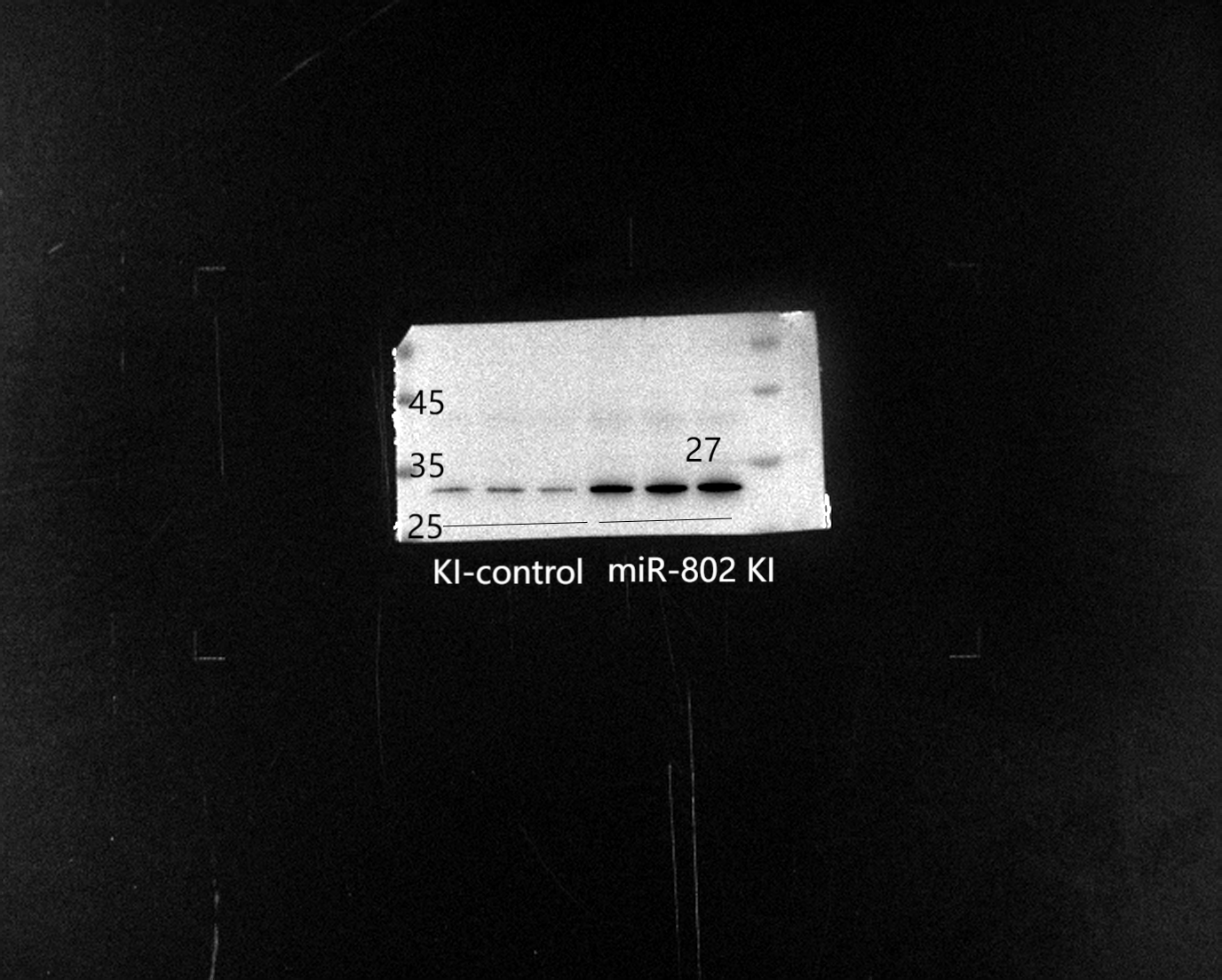

Supplement: Figure 6—source data 9. — The original files of the full raw unedited blots of some major canonical NF-κB signaling targets in the epiWAT of Mir802 KI mice(n=3). [file elife-99162-fig6-data9.zip › MnSOD.png]

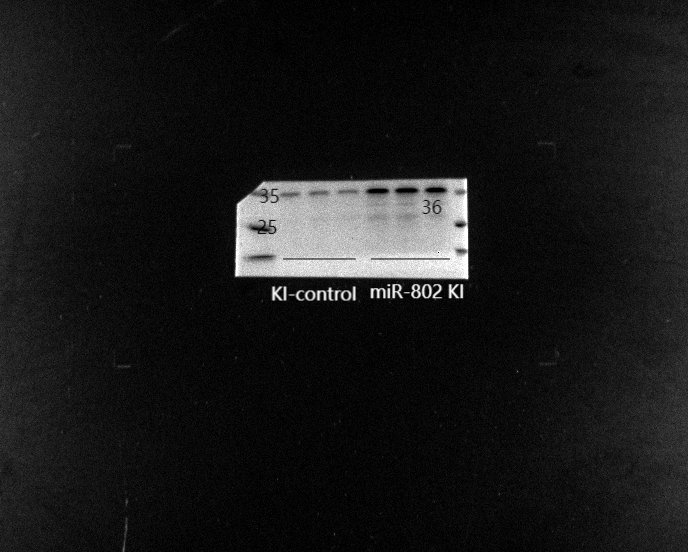

Supplement: Figure 6—source data 9. — The original files of the full raw unedited blots of some major canonical NF-κB signaling targets in the epiWAT of Mir802 KI mice(n=3). [file elife-99162-fig6-data9.zip › P-IkB╬▒.png]

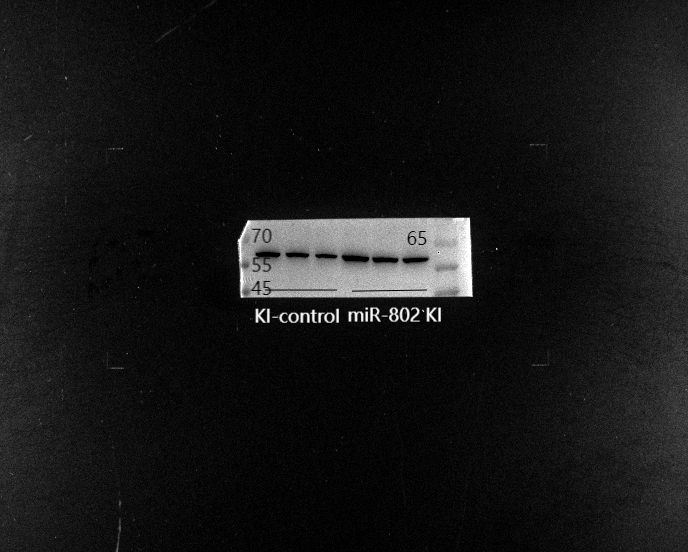

Supplement: Figure 6—source data 9. — The original files of the full raw unedited blots of some major canonical NF-κB signaling targets in the epiWAT of Mir802 KI mice(n=3). [file elife-99162-fig6-data9.zip › P-P65.png]

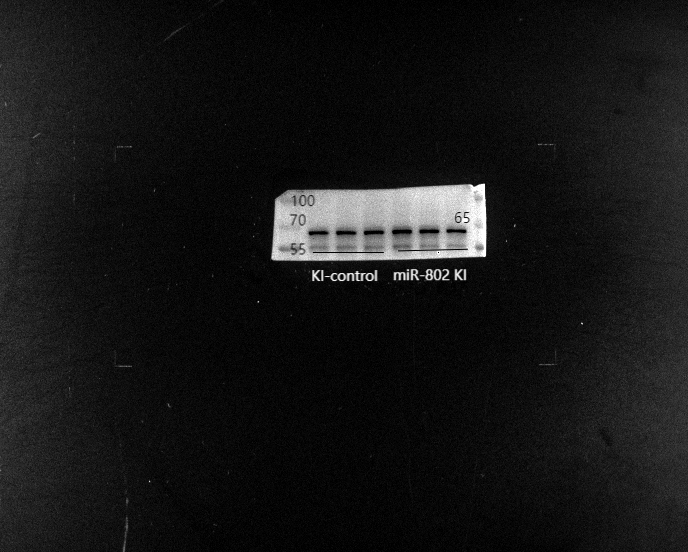

Supplement: Figure 6—source data 9. — The original files of the full raw unedited blots of some major canonical NF-κB signaling targets in the epiWAT of Mir802 KI mice(n=3). [file elife-99162-fig6-data9.zip › P65.png]

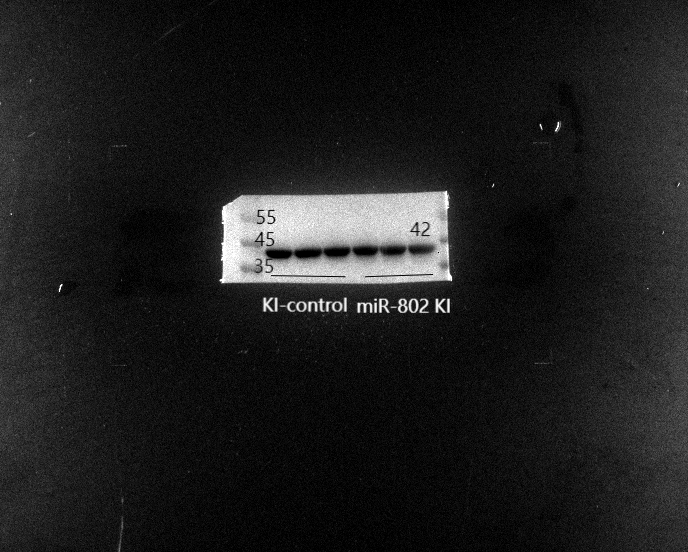

Supplement: Figure 6—source data 9. — The original files of the full raw unedited blots of some major canonical NF-κB signaling targets in the epiWAT of Mir802 KI mice(n=3). [file elife-99162-fig6-data9.zip › ╬▓-Actin.png]

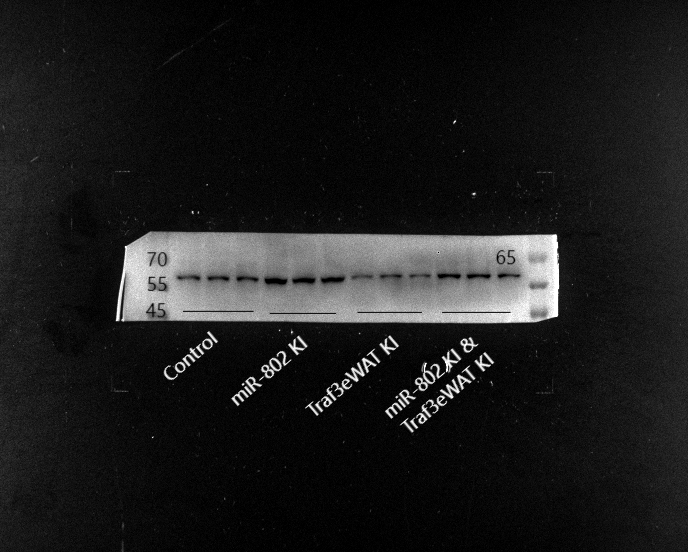

Supplement: Figure 6—source data 10. — The original files of the full raw unedited blots of some major canonical NF-κB signaling targets in the epiWAT of Mir802 KI mice and Traf3 eWAT OE rescued mice (n=3). [file elife-99162-fig6-data10.zip › P-P65.png]

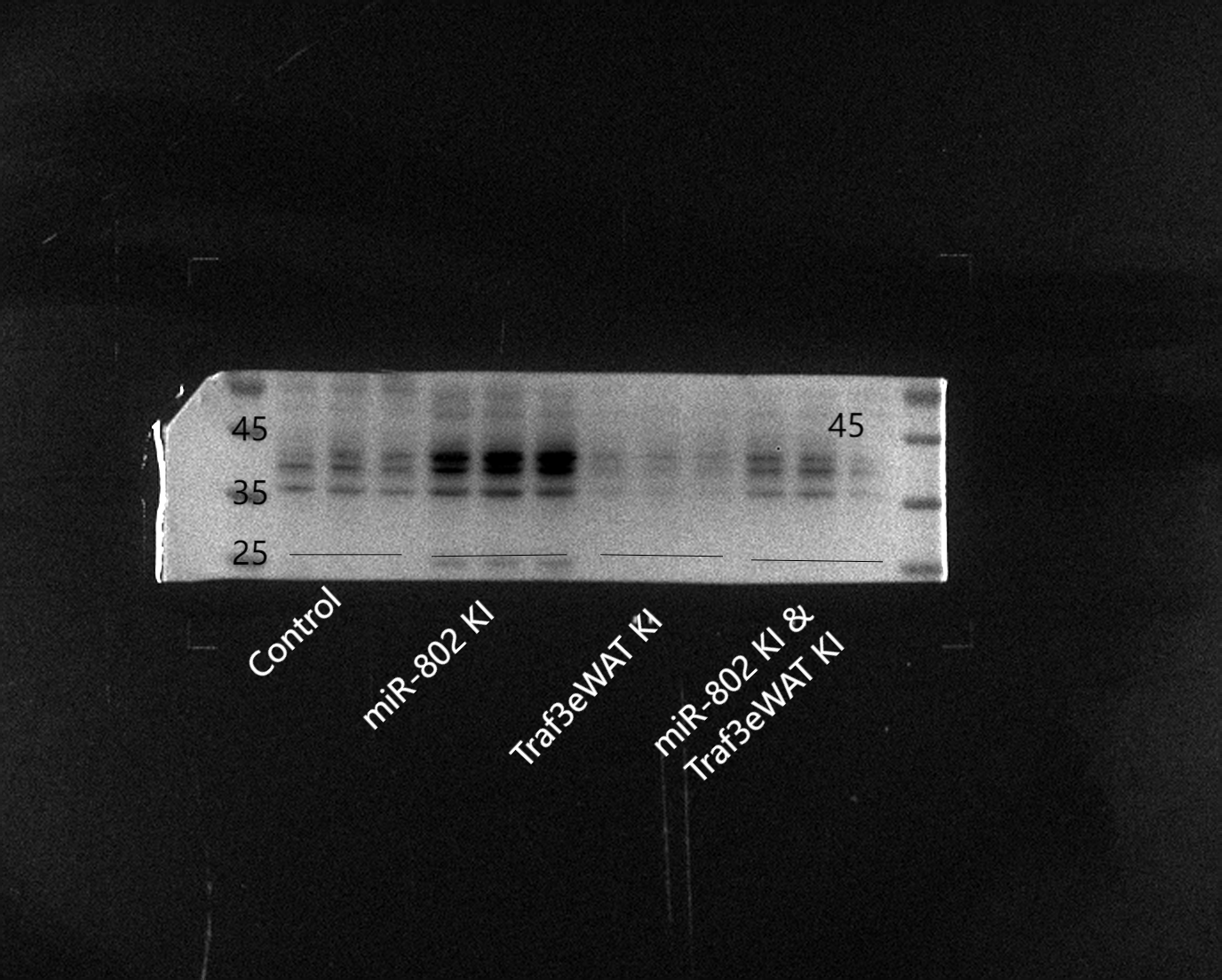

Supplement: Figure 6—source data 10. — The original files of the full raw unedited blots of some major canonical NF-κB signaling targets in the epiWAT of Mir802 KI mice and Traf3 eWAT OE rescued mice (n=3). [file elife-99162-fig6-data10.zip › FasL.png]

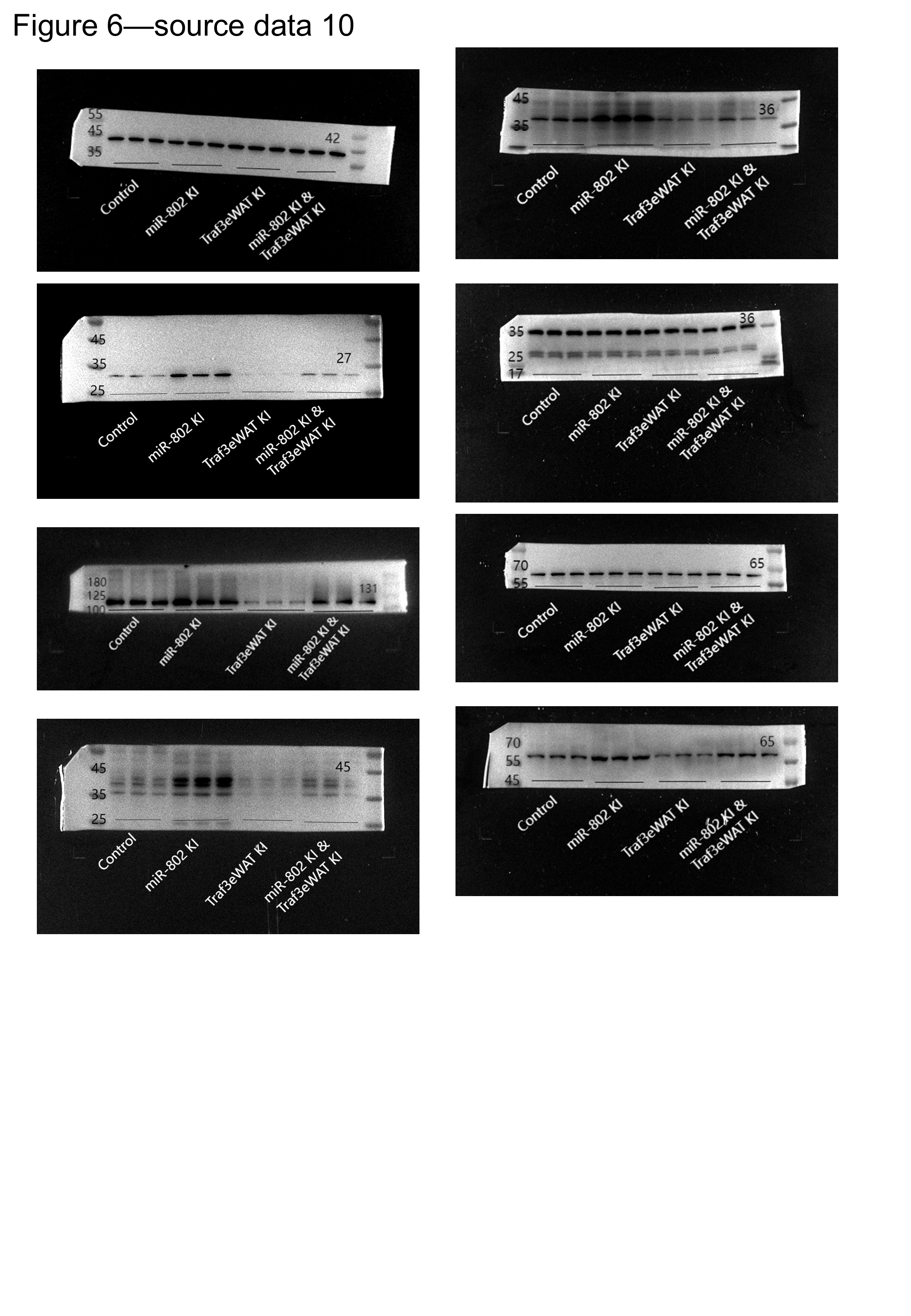

Supplement: Figure 6—source data 10. — The original files of the full raw unedited blots of some major canonical NF-κB signaling targets in the epiWAT of Mir802 KI mice and Traf3 eWAT OE rescued mice (n=3). [file elife-99162-fig6-data10.zip › Figure 6ΓÇösource data 10.tif]

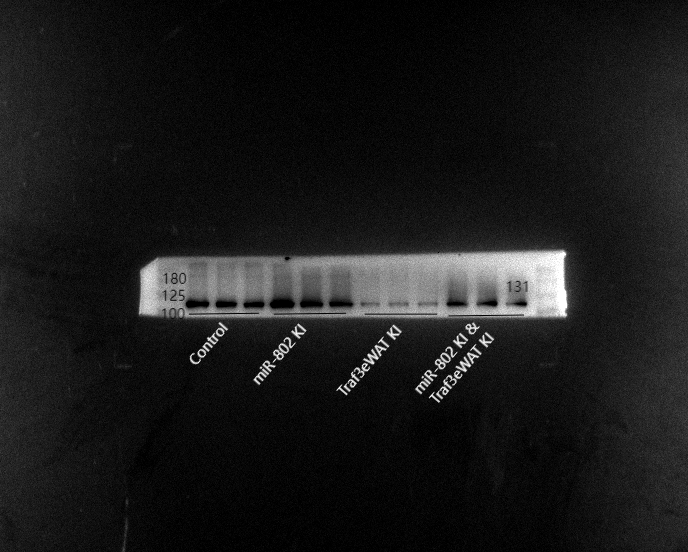

Supplement: Figure 6—source data 10. — The original files of the full raw unedited blots of some major canonical NF-κB signaling targets in the epiWAT of Mir802 KI mice and Traf3 eWAT OE rescued mice (n=3). [file elife-99162-fig6-data10.zip › iNOS.png]

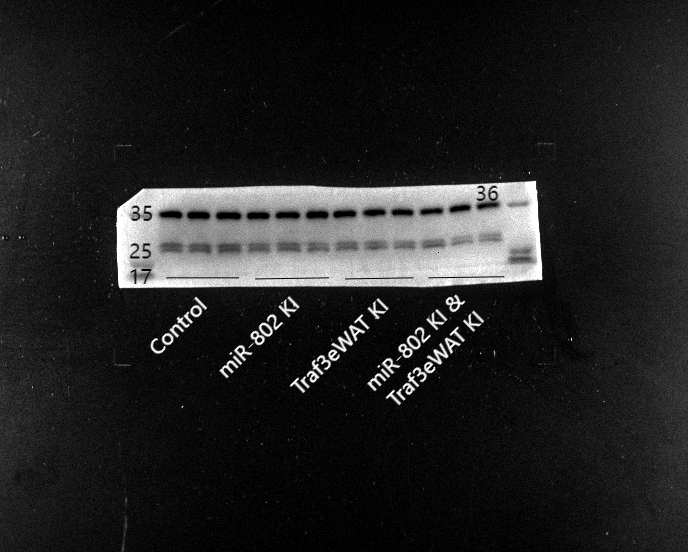

Supplement: Figure 6—source data 10. — The original files of the full raw unedited blots of some major canonical NF-κB signaling targets in the epiWAT of Mir802 KI mice and Traf3 eWAT OE rescued mice (n=3). [file elife-99162-fig6-data10.zip › I╬║B-╬▒.png]

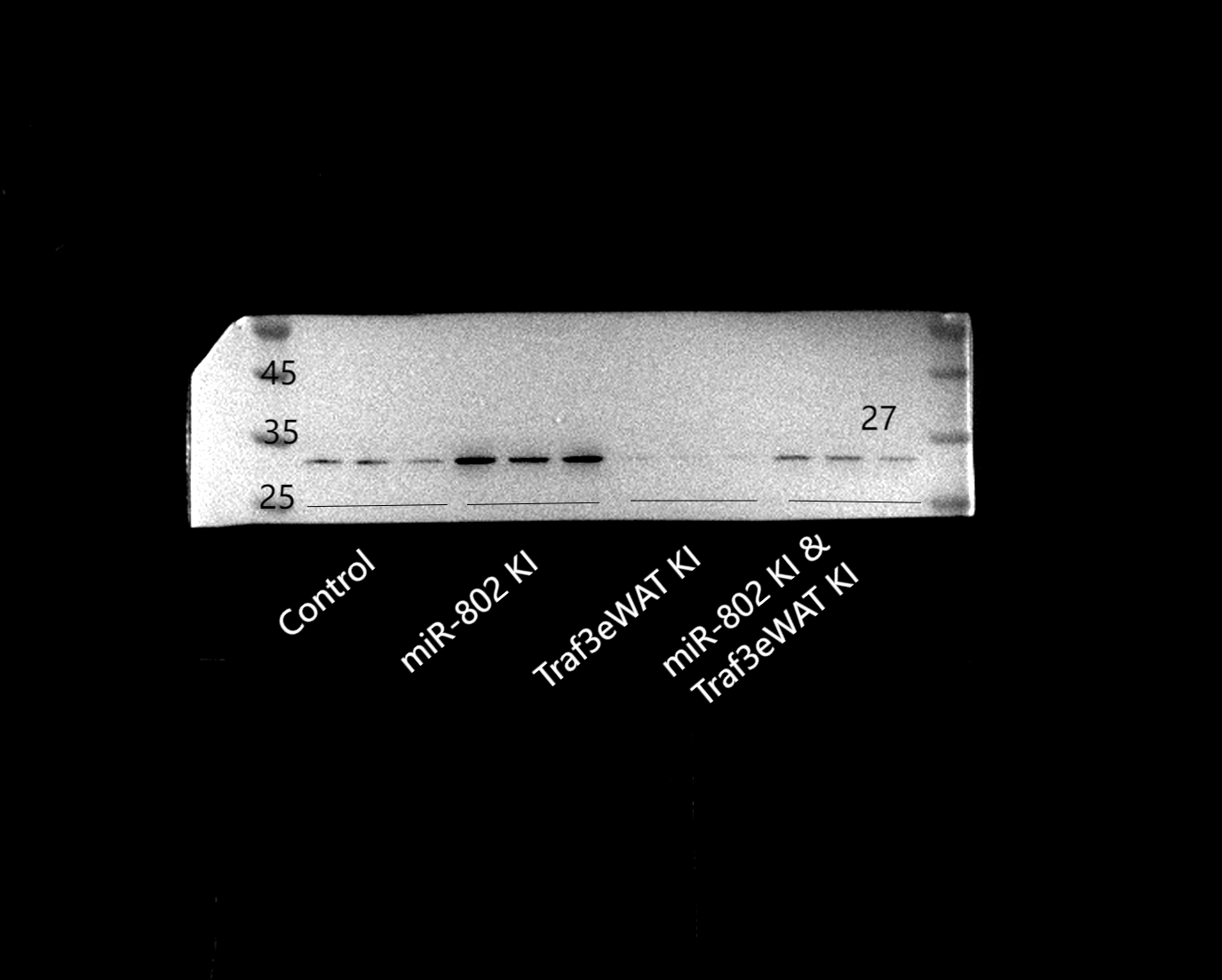

Supplement: Figure 6—source data 10. — The original files of the full raw unedited blots of some major canonical NF-κB signaling targets in the epiWAT of Mir802 KI mice and Traf3 eWAT OE rescued mice (n=3). [file elife-99162-fig6-data10.zip › MnSOD.png]

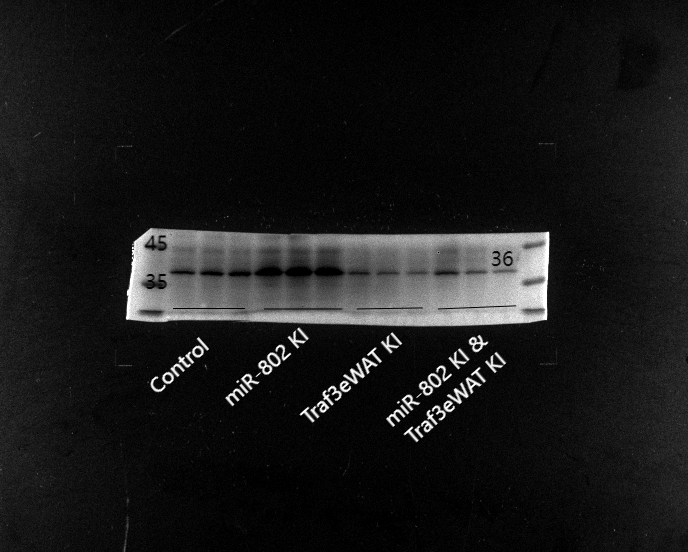

Supplement: Figure 6—source data 10. — The original files of the full raw unedited blots of some major canonical NF-κB signaling targets in the epiWAT of Mir802 KI mice and Traf3 eWAT OE rescued mice (n=3). [file elife-99162-fig6-data10.zip › P-I╬║B-╬▒.png]

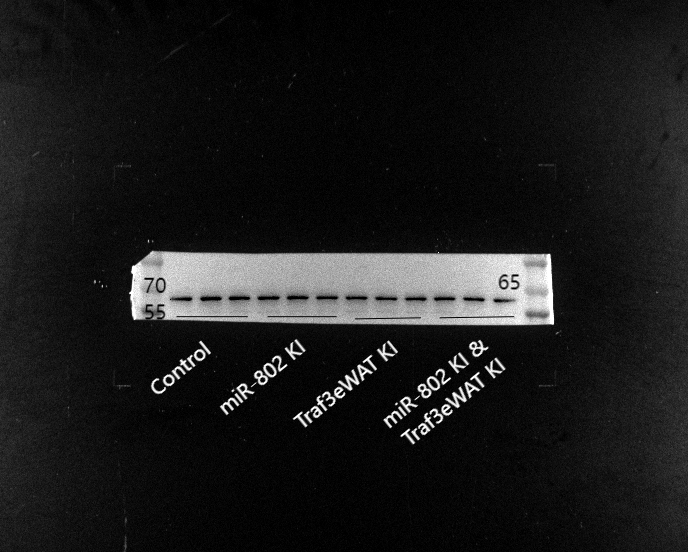

Supplement: Figure 6—source data 10. — The original files of the full raw unedited blots of some major canonical NF-κB signaling targets in the epiWAT of Mir802 KI mice and Traf3 eWAT OE rescued mice (n=3). [file elife-99162-fig6-data10.zip › P65.png]

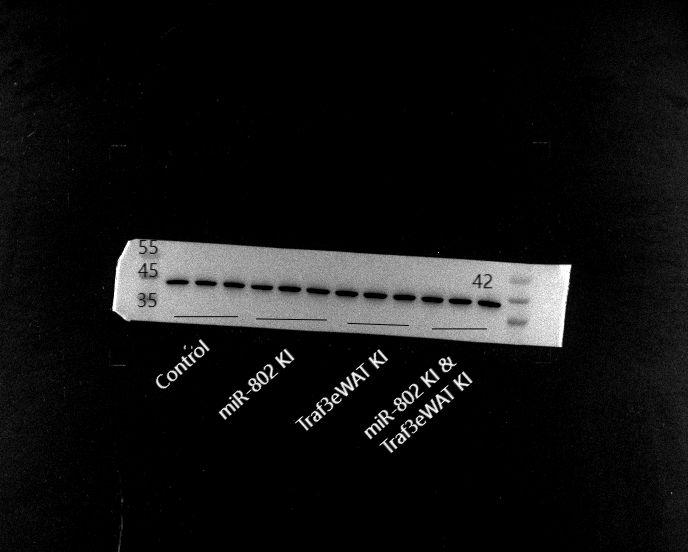

Supplement: Figure 6—source data 10. — The original files of the full raw unedited blots of some major canonical NF-κB signaling targets in the epiWAT of Mir802 KI mice and Traf3 eWAT OE rescued mice (n=3). [file elife-99162-fig6-data10.zip › ╬▓-Actin.png]

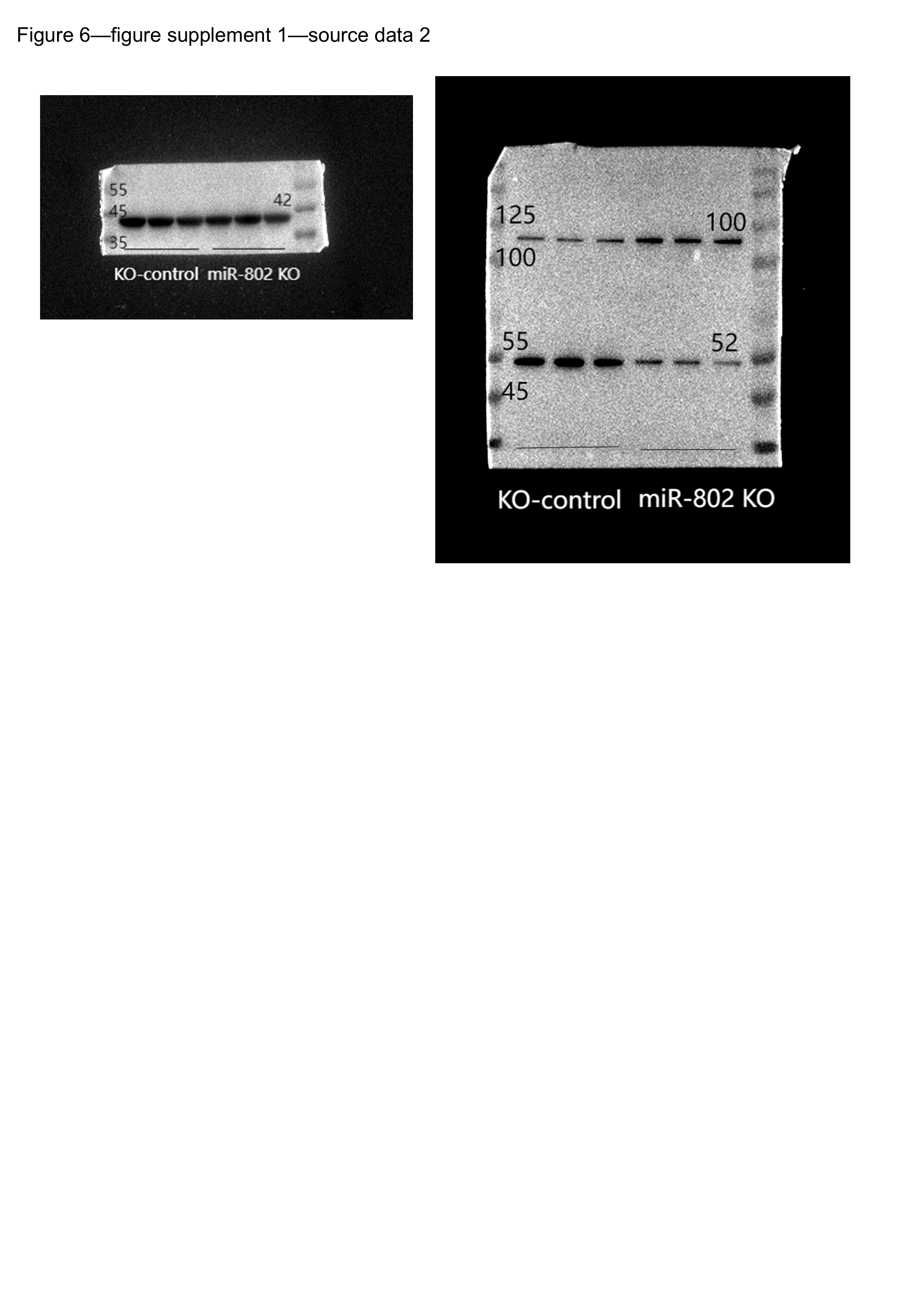

Supplement: Figure 6—figure supplement 1—source data 1. — The original files of the full raw unedited blots of TRAF3, NIK, and β-Actin in in the epiWAT of Mir802 KO mice (n=3). [file elife-99162-fig6-figsupp1-data1.zip › Figure 6ΓÇöfigure supplement 1ΓÇösource data 1.tif]

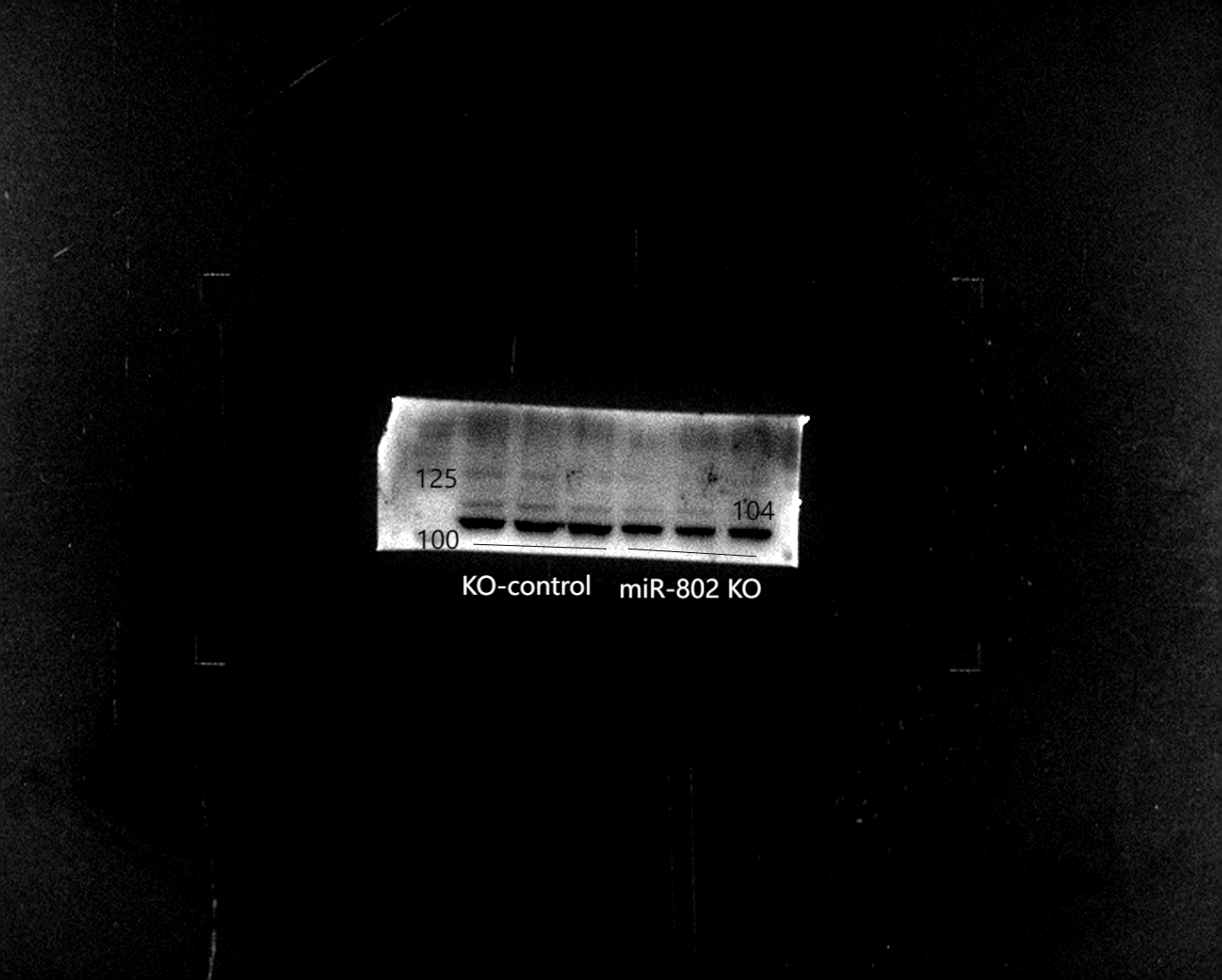

Supplement: Figure 6—figure supplement 1—source data 1. — The original files of the full raw unedited blots of TRAF3, NIK, and β-Actin in in the epiWAT of Mir802 KO mice (n=3). [file elife-99162-fig6-figsupp1-data1.zip › NIK.png]

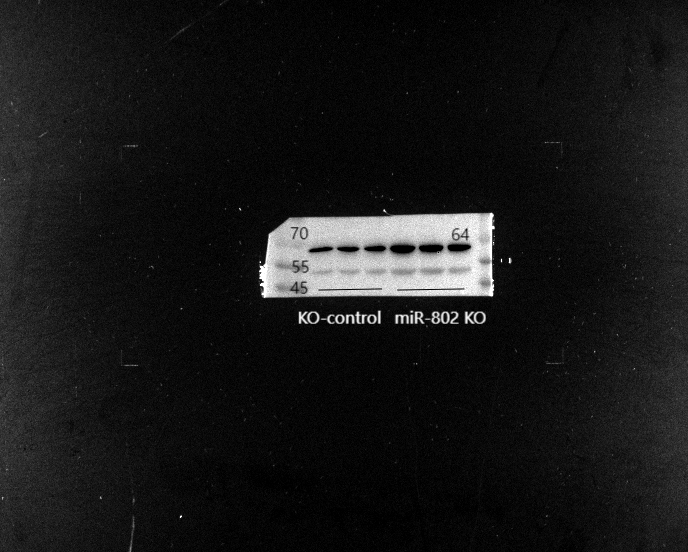

Supplement: Figure 6—figure supplement 1—source data 1. — The original files of the full raw unedited blots of TRAF3, NIK, and β-Actin in in the epiWAT of Mir802 KO mice (n=3). [file elife-99162-fig6-figsupp1-data1.zip › TRAF3.png]

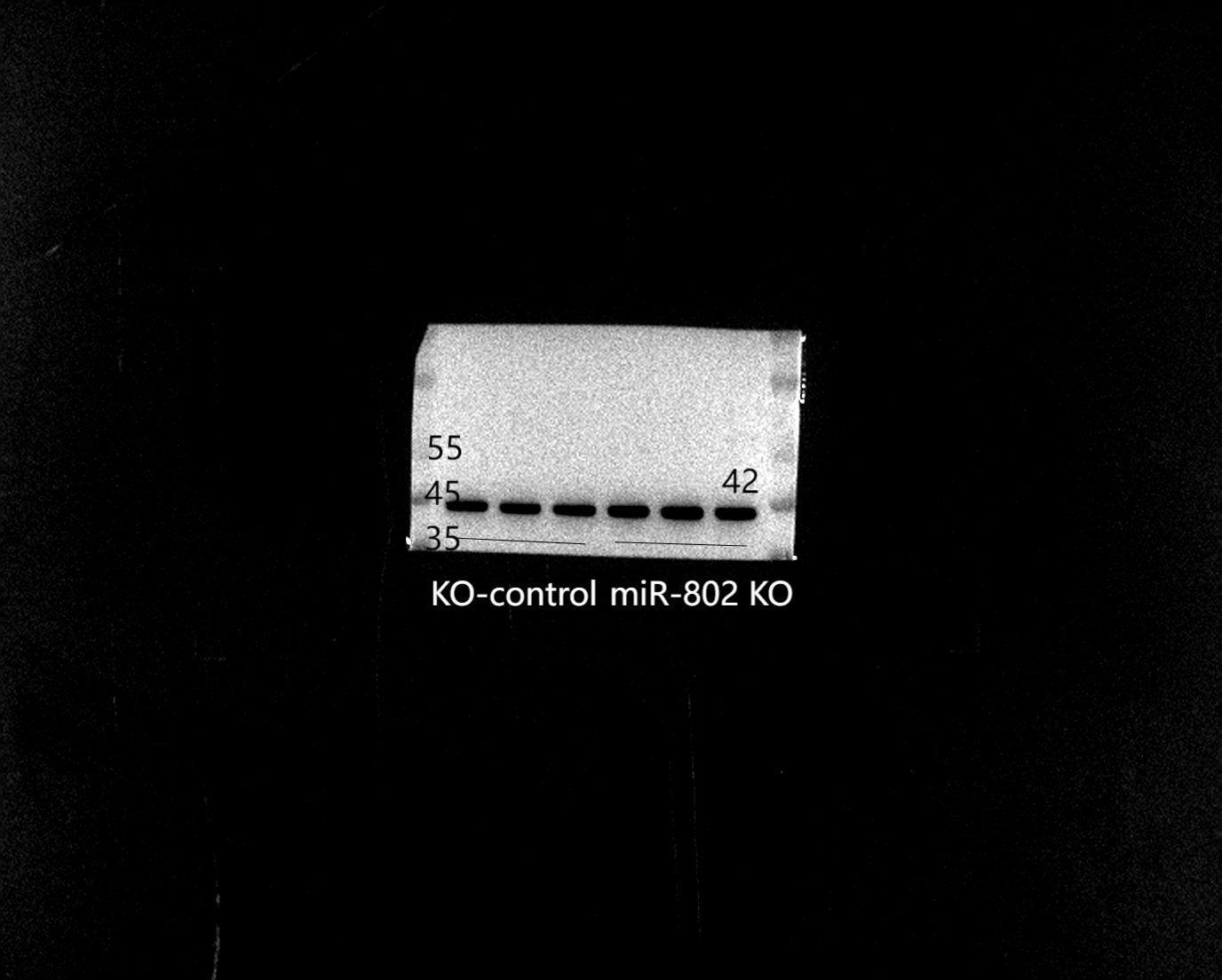

Supplement: Figure 6—figure supplement 1—source data 1. — The original files of the full raw unedited blots of TRAF3, NIK, and β-Actin in in the epiWAT of Mir802 KO mice (n=3). [file elife-99162-fig6-figsupp1-data1.zip › ╬▓-Actin.png]

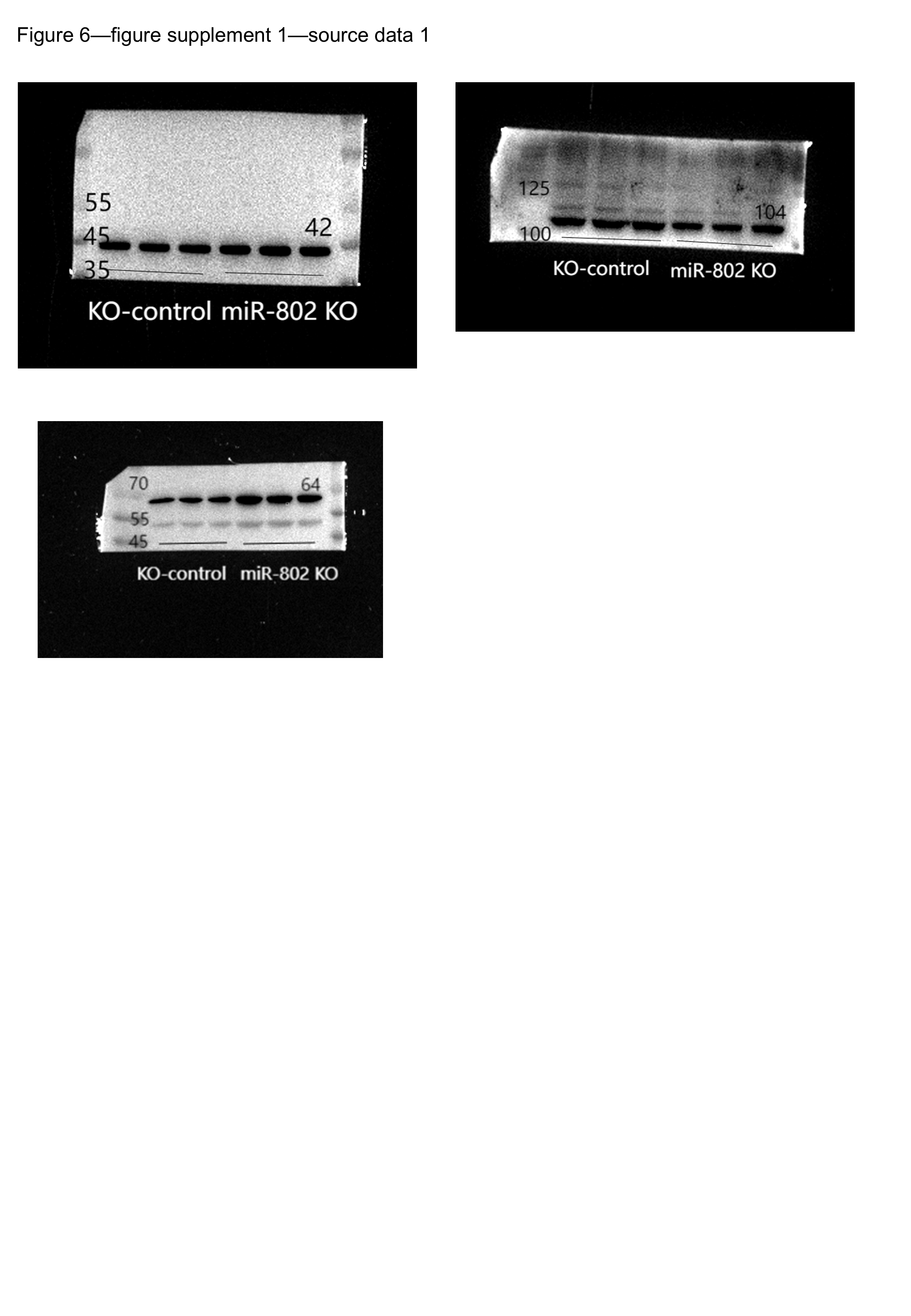

Supplement: Figure 6—figure supplement 1—source data 2. — The original files of the full raw unedited blots of p100/p52 and β-Actin in the epiWAT of Mir802 KO mice (n=3). [file elife-99162-fig6-figsupp1-data2.zip › Figure 6ΓÇöfigure supplement 1ΓÇösource data 2.tif]

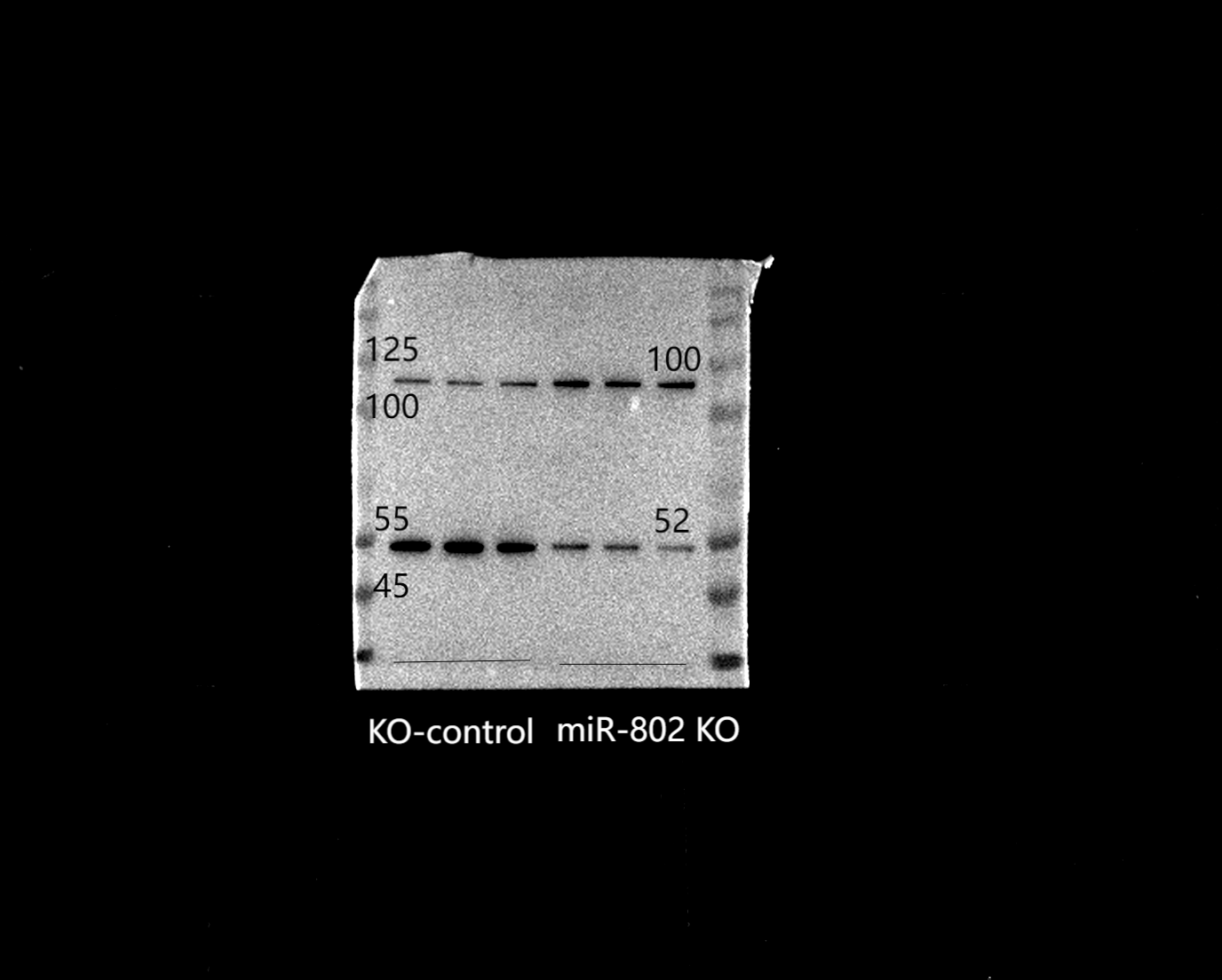

Supplement: Figure 6—figure supplement 1—source data 2. — The original files of the full raw unedited blots of p100/p52 and β-Actin in the epiWAT of Mir802 KO mice (n=3). [file elife-99162-fig6-figsupp1-data2.zip › p100-p52-4.png]

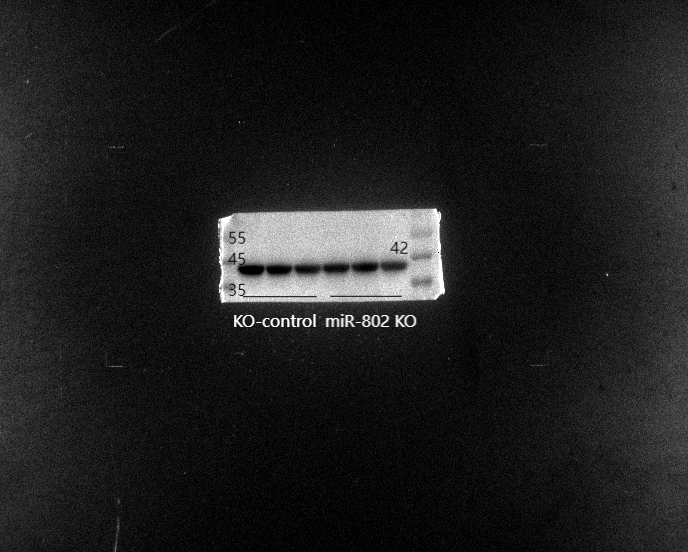

Supplement: Figure 6—figure supplement 1—source data 2. — The original files of the full raw unedited blots of p100/p52 and β-Actin in the epiWAT of Mir802 KO mice (n=3). [file elife-99162-fig6-figsupp1-data2.zip › ╬▓-Actin.png]

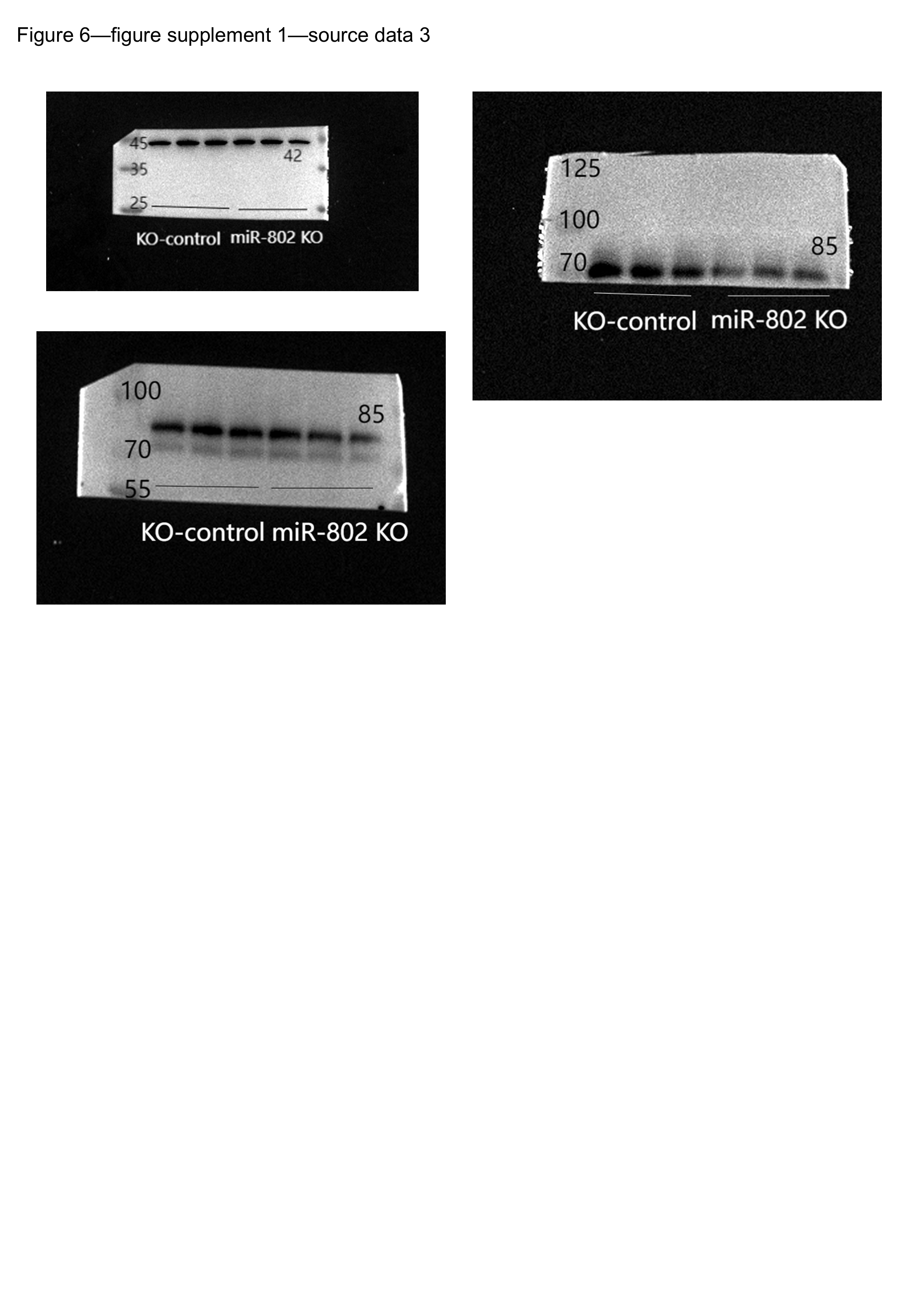

Supplement: Figure 6—figure supplement 1—source data 3. — The original files of the full raw unedited blots of P-IKK-α, IKK-α, and β-Actin in the epiWAT of Mir802 KO mice (n=3). [file elife-99162-fig6-figsupp1-data3.zip › Figure 6ΓÇöfigure supplement 1ΓÇösource data 3.tif]

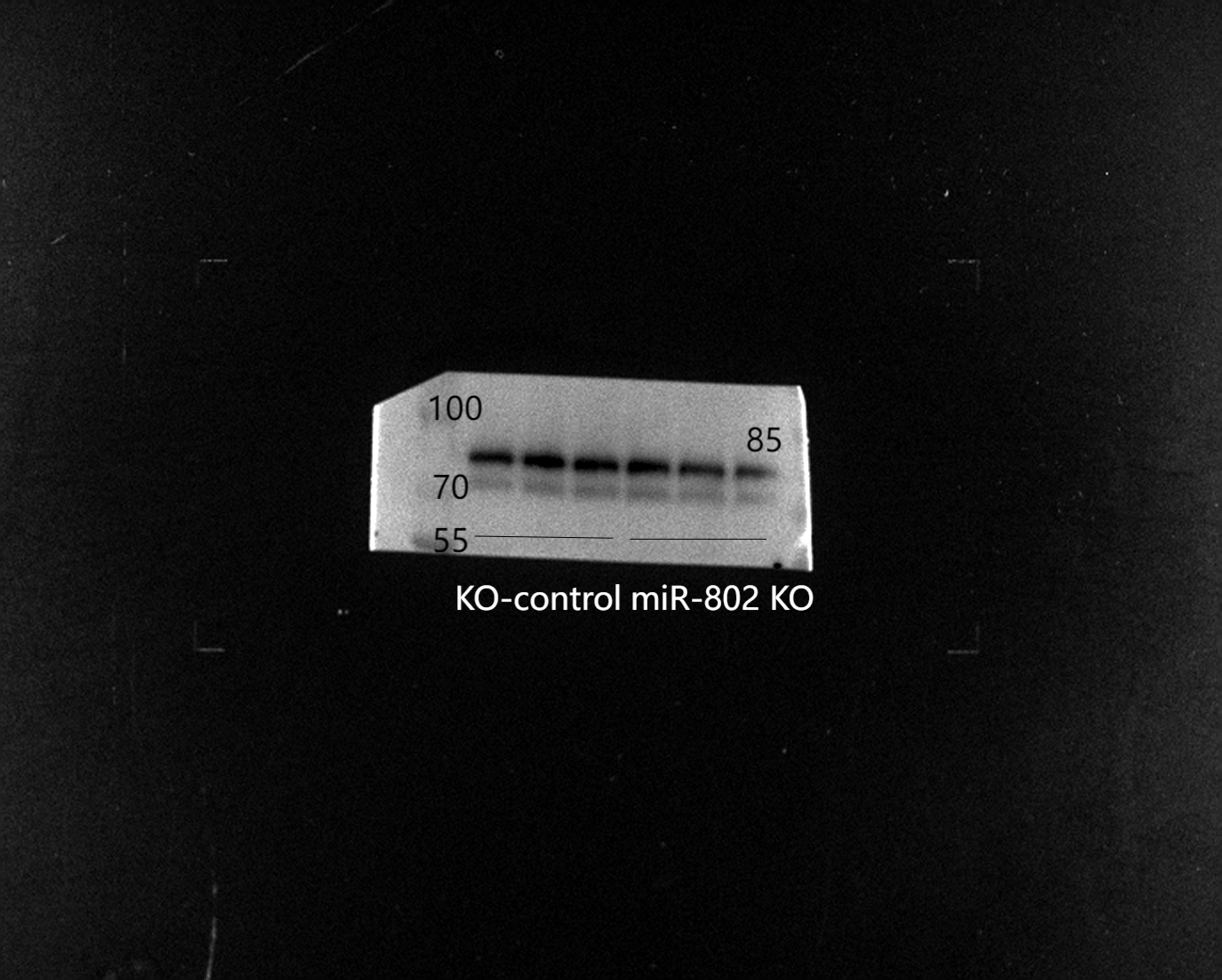

Supplement: Figure 6—figure supplement 1—source data 3. — The original files of the full raw unedited blots of P-IKK-α, IKK-α, and β-Actin in the epiWAT of Mir802 KO mice (n=3). [file elife-99162-fig6-figsupp1-data3.zip › IKK-╬▒.png]

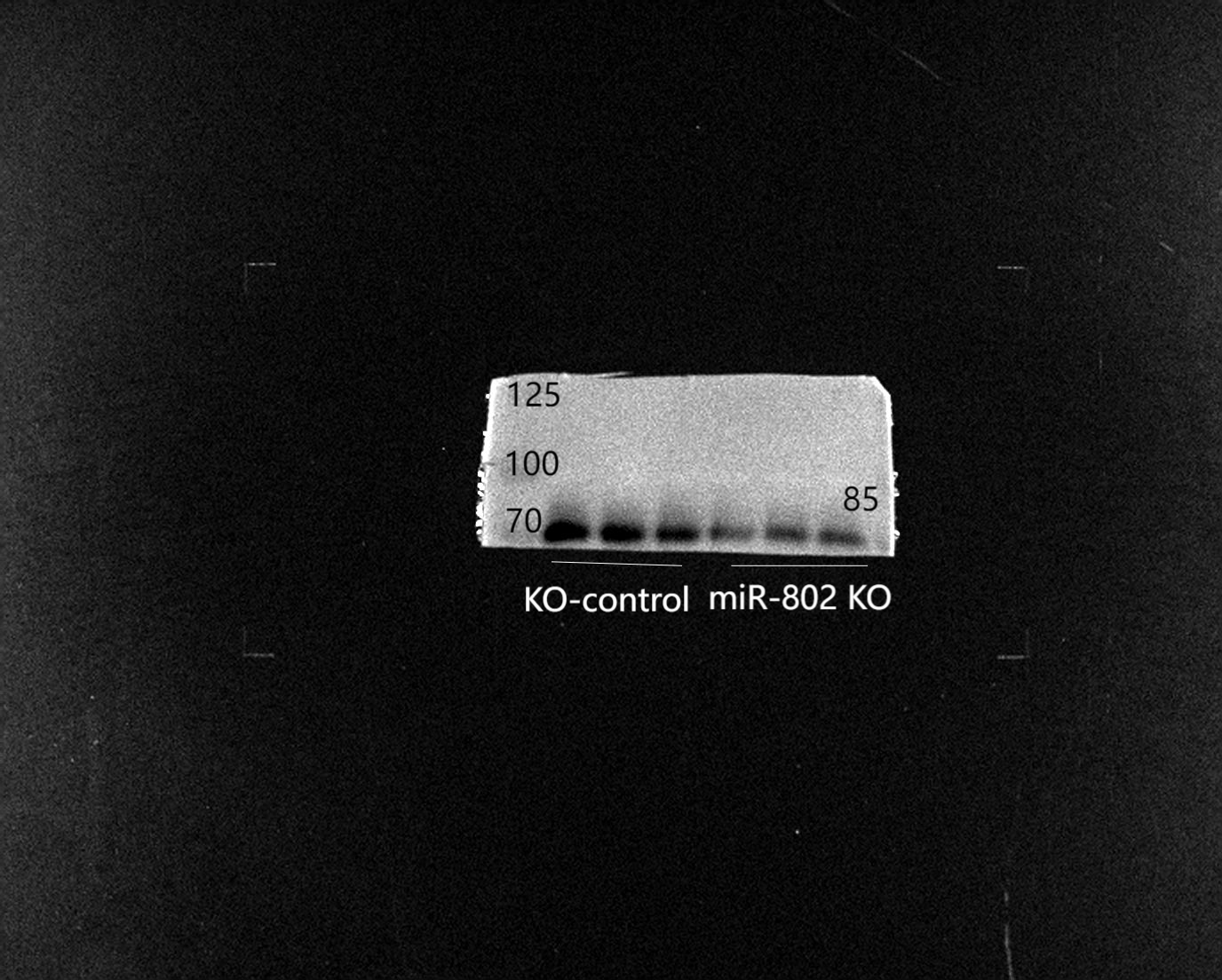

Supplement: Figure 6—figure supplement 1—source data 3. — The original files of the full raw unedited blots of P-IKK-α, IKK-α, and β-Actin in the epiWAT of Mir802 KO mice (n=3). [file elife-99162-fig6-figsupp1-data3.zip › P-IKK-╬▒.png]

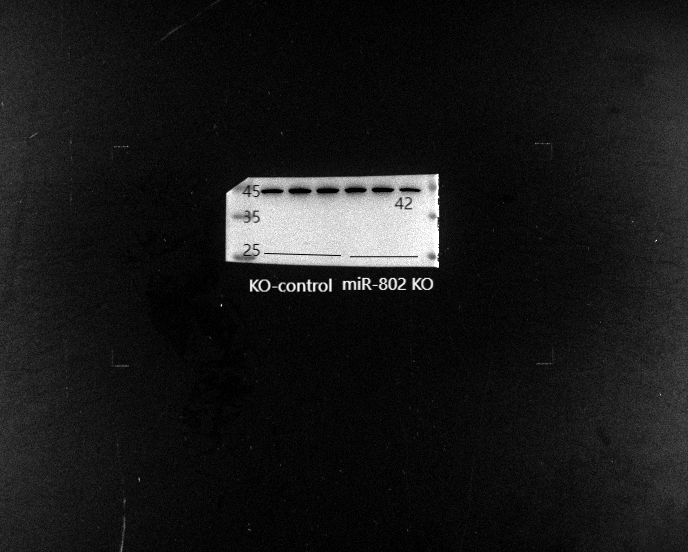

Supplement: Figure 6—figure supplement 1—source data 3. — The original files of the full raw unedited blots of P-IKK-α, IKK-α, and β-Actin in the epiWAT of Mir802 KO mice (n=3). [file elife-99162-fig6-figsupp1-data3.zip › ╬▓-Actin.png]

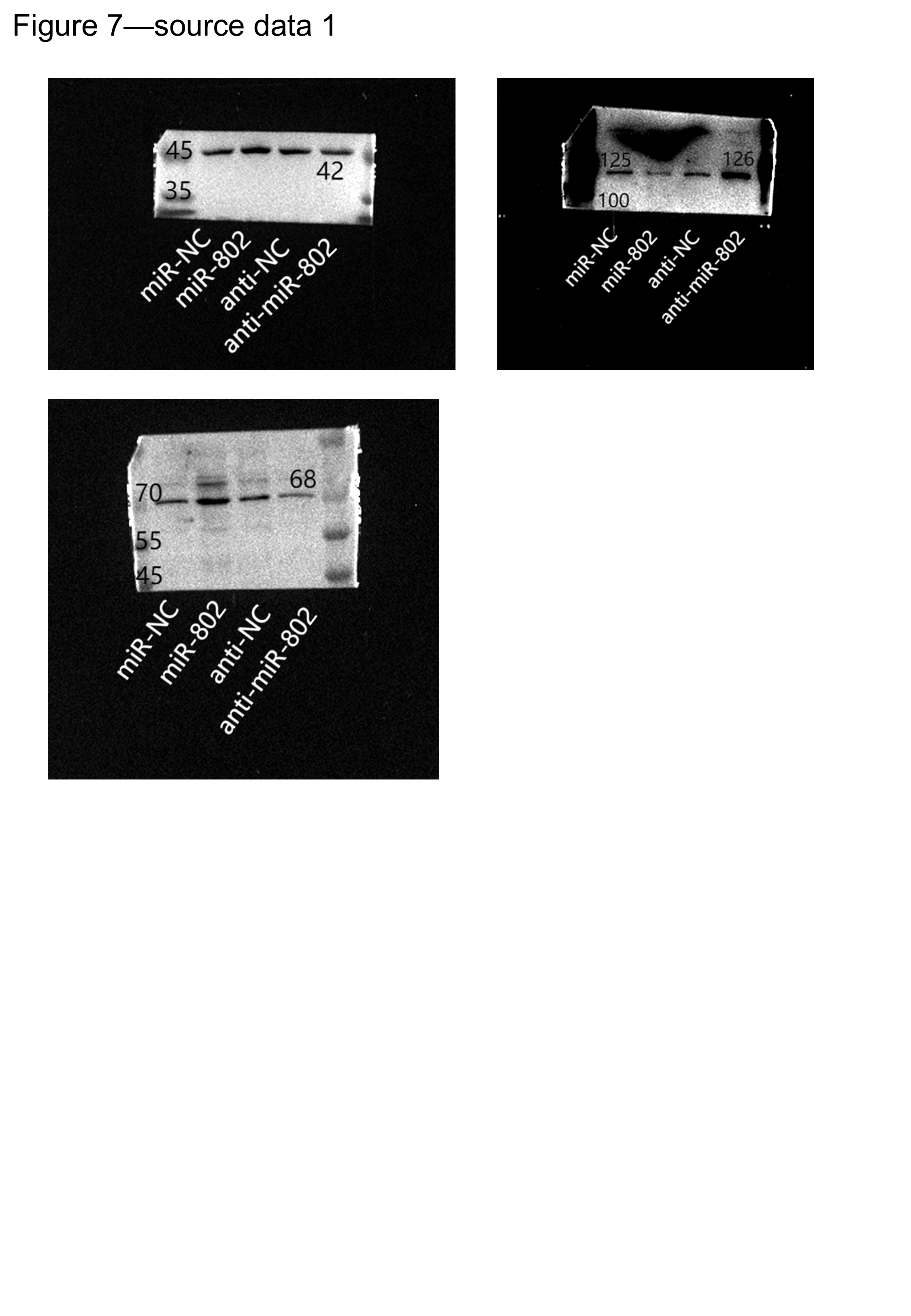

Supplement: Figure 7—source data 1. — The original files of the full raw unedited blots of m-SREBP1, P-SREBP1, and β-Actin in mature 3T3-L1 cells transfected with Mir802 mimics or Mir802 inhibitor. [file elife-99162-fig7-data1.zip › Figure 7ΓÇösource data 1.tif]

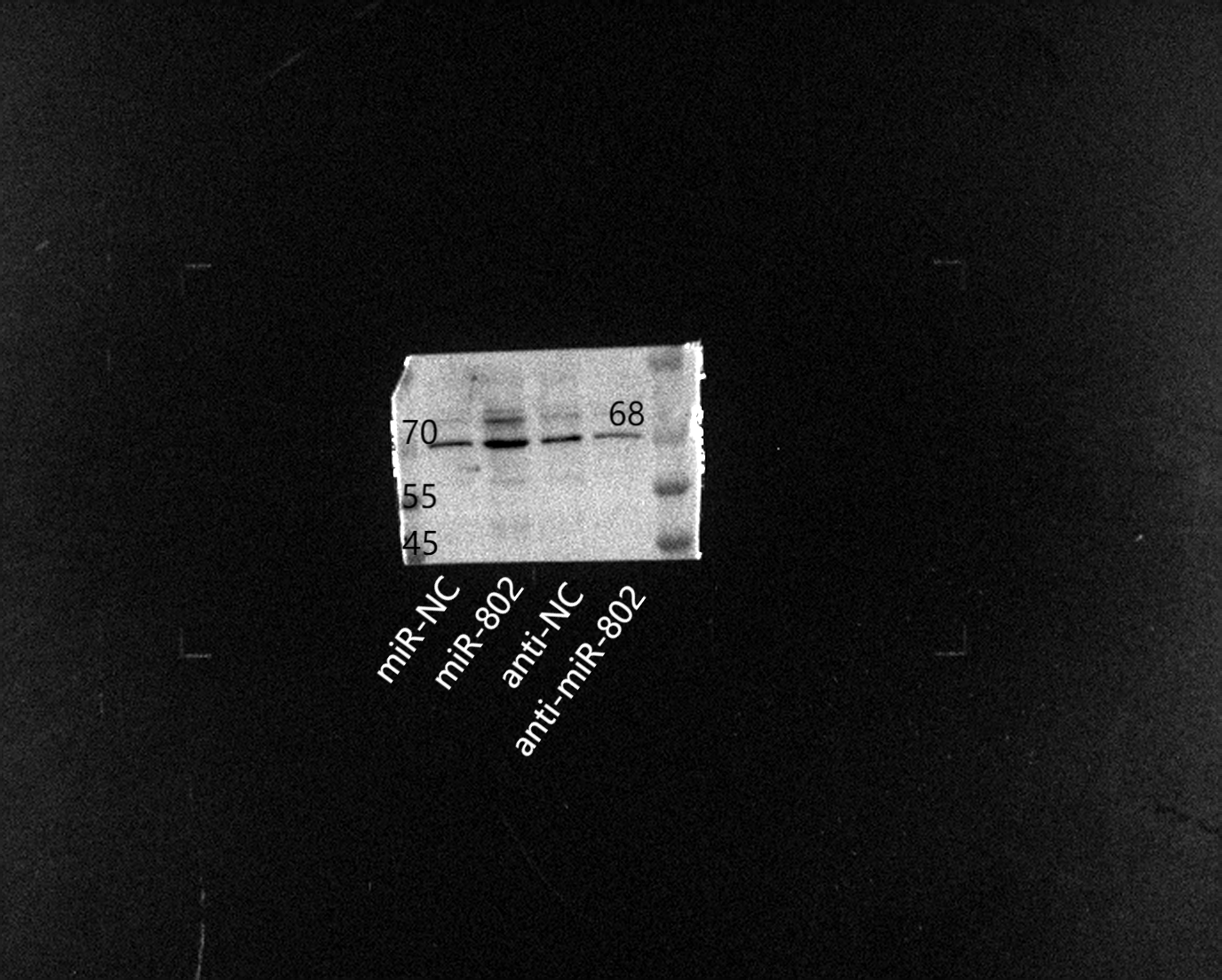

Supplement: Figure 7—source data 1. — The original files of the full raw unedited blots of m-SREBP1, P-SREBP1, and β-Actin in mature 3T3-L1 cells transfected with Mir802 mimics or Mir802 inhibitor. [file elife-99162-fig7-data1.zip › m-SREBP1-4.png]

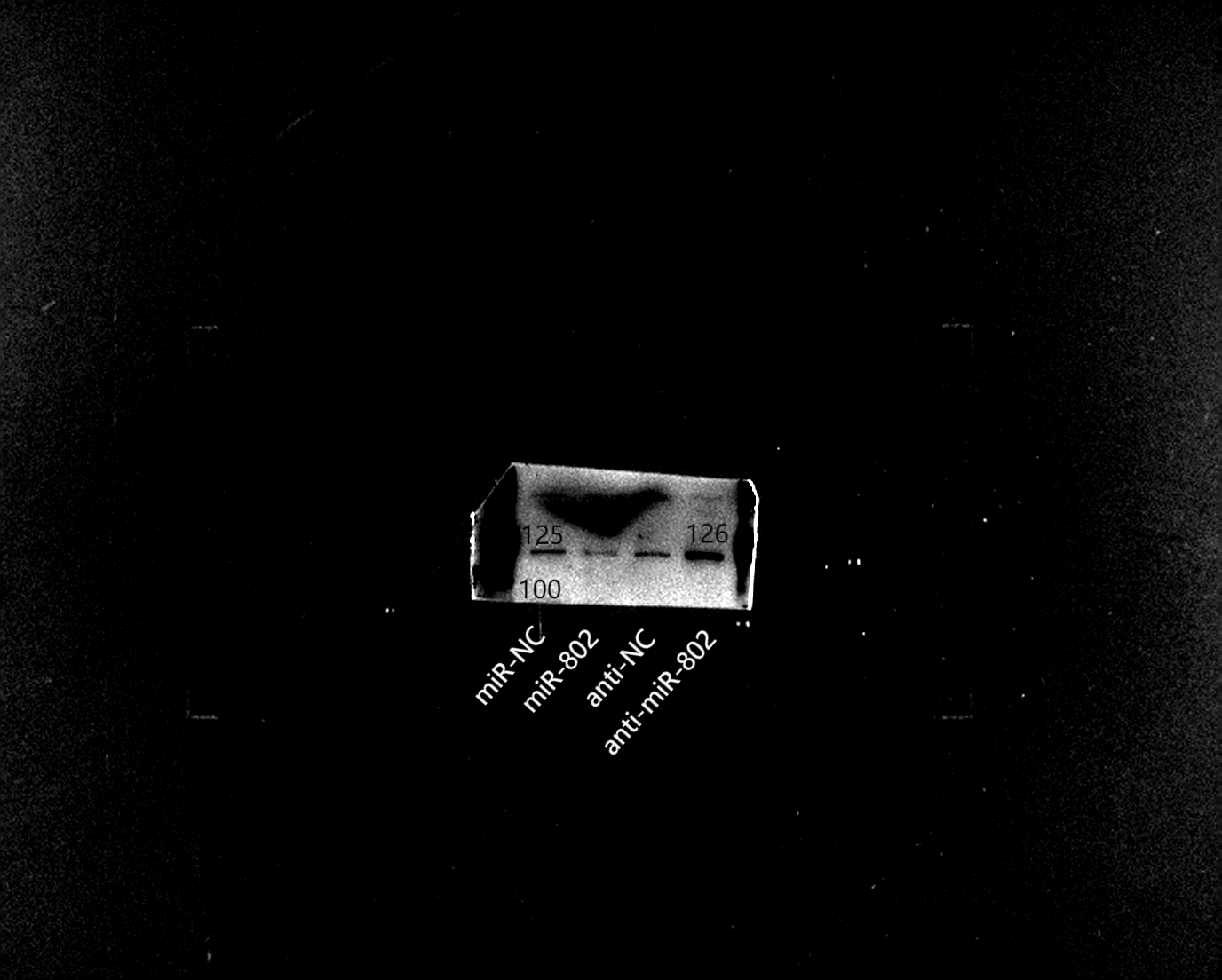

Supplement: Figure 7—source data 1. — The original files of the full raw unedited blots of m-SREBP1, P-SREBP1, and β-Actin in mature 3T3-L1 cells transfected with Mir802 mimics or Mir802 inhibitor. [file elife-99162-fig7-data1.zip › p-SREBP1-4.png]

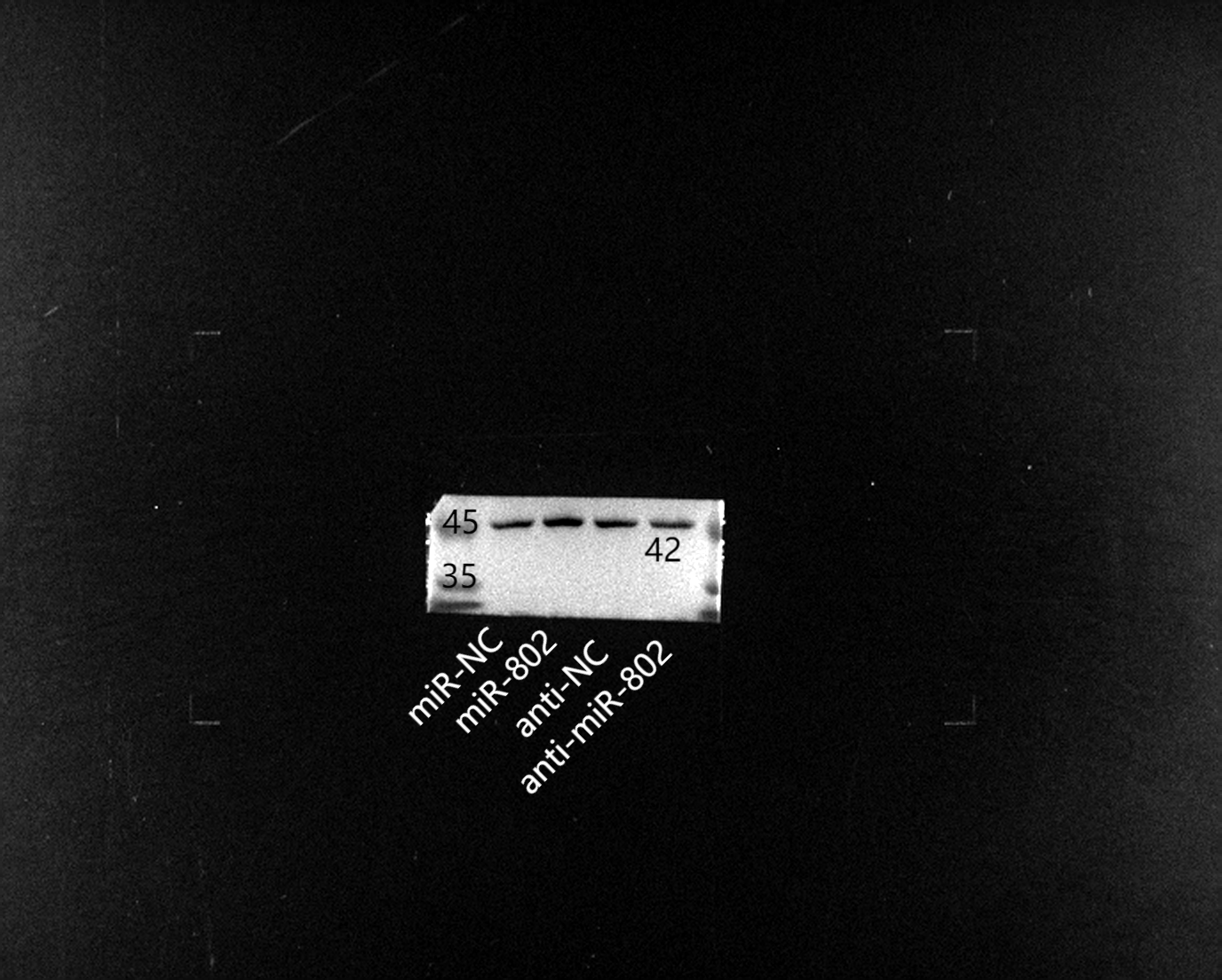

Supplement: Figure 7—source data 1. — The original files of the full raw unedited blots of m-SREBP1, P-SREBP1, and β-Actin in mature 3T3-L1 cells transfected with Mir802 mimics or Mir802 inhibitor. [file elife-99162-fig7-data1.zip › ╬▓-Actin.png]

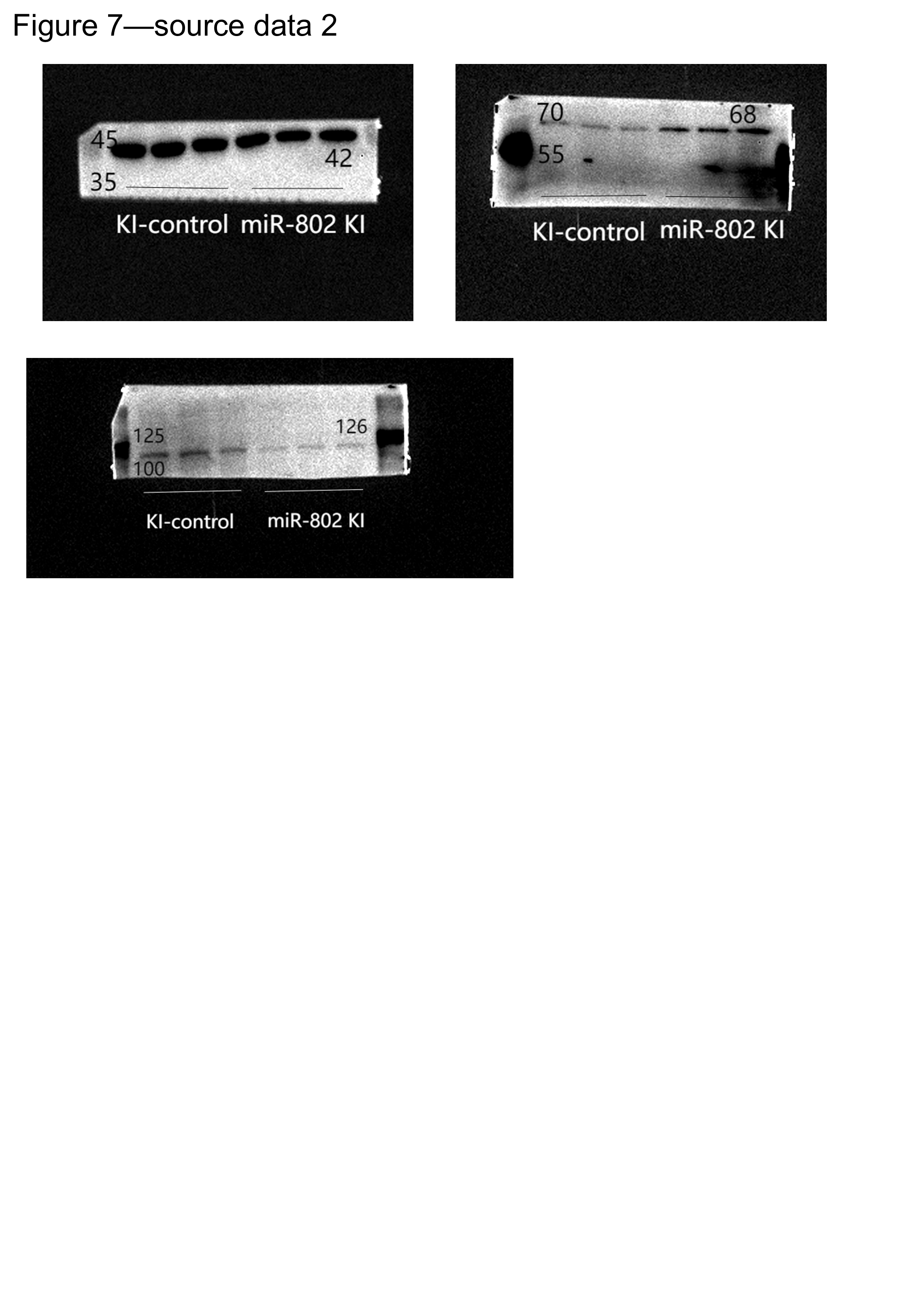

Supplement: Figure 7—source data 2. — The original files of the full raw unedited blots of m-SREBP1, P-SREBP1, and β-Actin in in the epiWAT of Mir802 KI mice (n=3). [file elife-99162-fig7-data2.zip › Figure 7ΓÇösource data 2.tif]

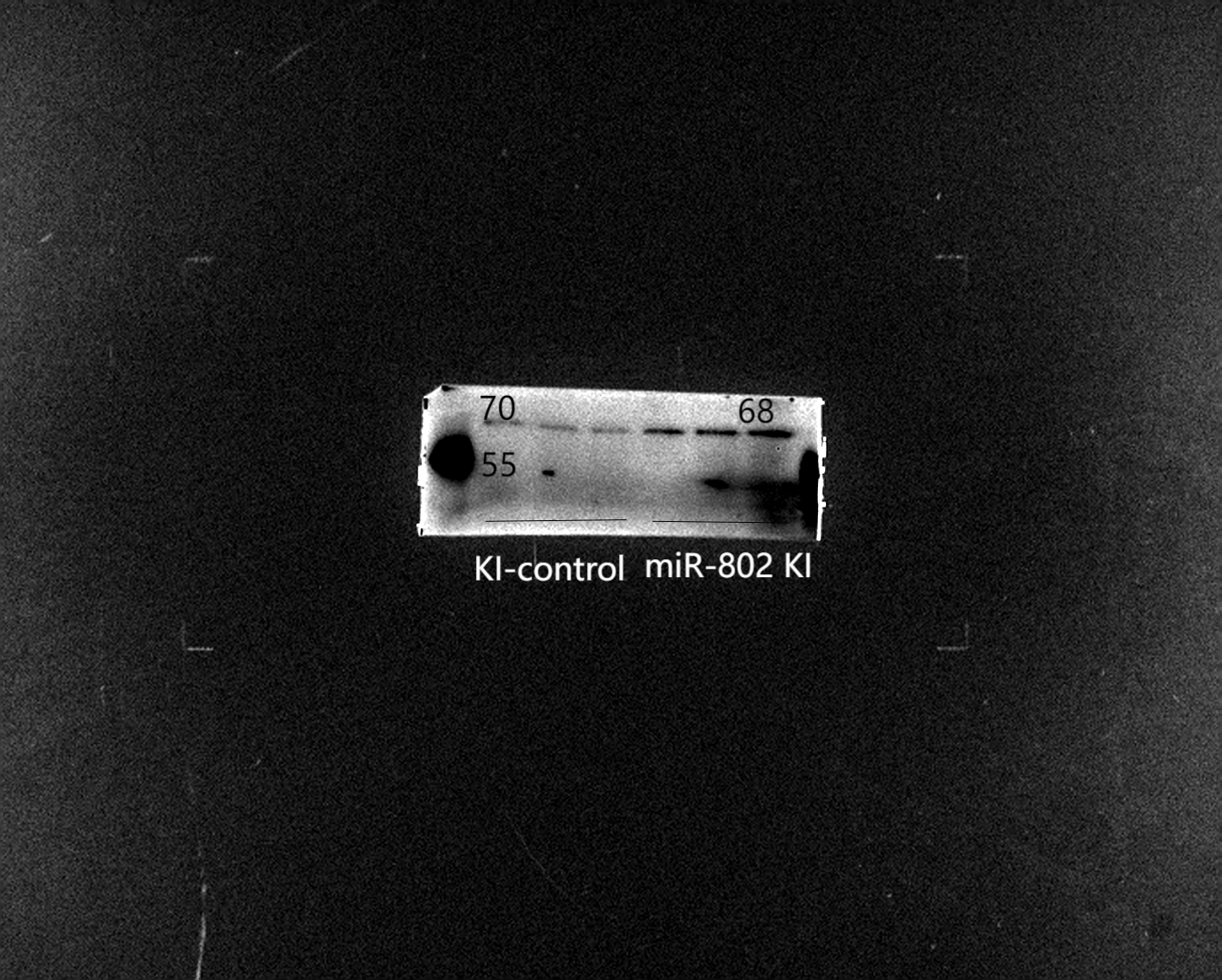

Supplement: Figure 7—source data 2. — The original files of the full raw unedited blots of m-SREBP1, P-SREBP1, and β-Actin in in the epiWAT of Mir802 KI mice (n=3). [file elife-99162-fig7-data2.zip › m-SREBP1-4.png]
