# Supplementary figures and images for: Adipocyte microRNA-802 promotes adipose tissue inflammation and insulin resistance by modulating macrophages in obesity (part 2 of 2)
Source: eLife. 2024 Nov 26;13:e99162. doi: 10.7554/eLife.99162 (PMC11651656; doi:10.7554/eLife.99162)

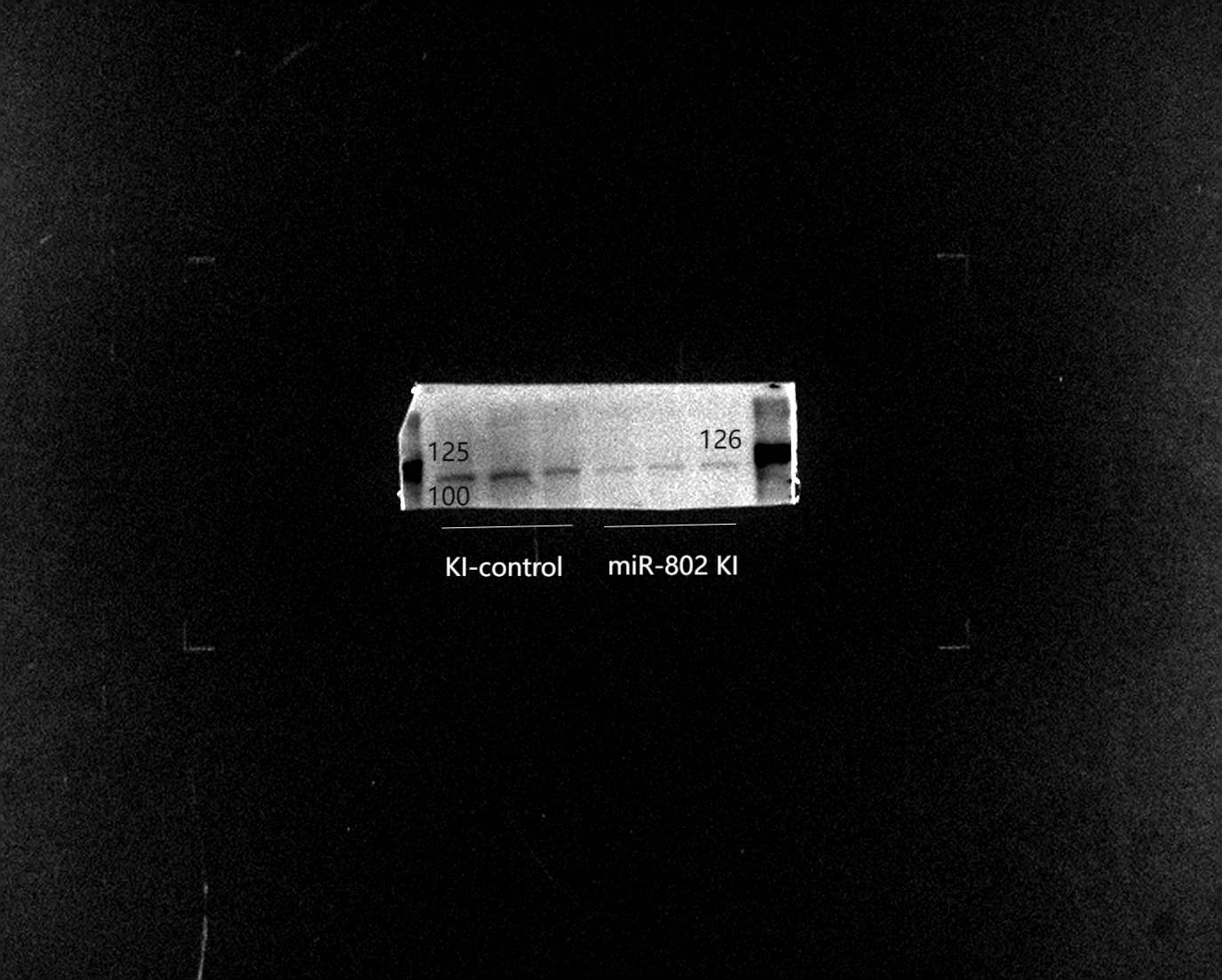

Supplement: Figure 7—source data 2. — The original files of the full raw unedited blots of m-SREBP1, P-SREBP1, and β-Actin in in the epiWAT of Mir802 KI mice (n=3). [file elife-99162-fig7-data2.zip › p-SREBP1-4.png]

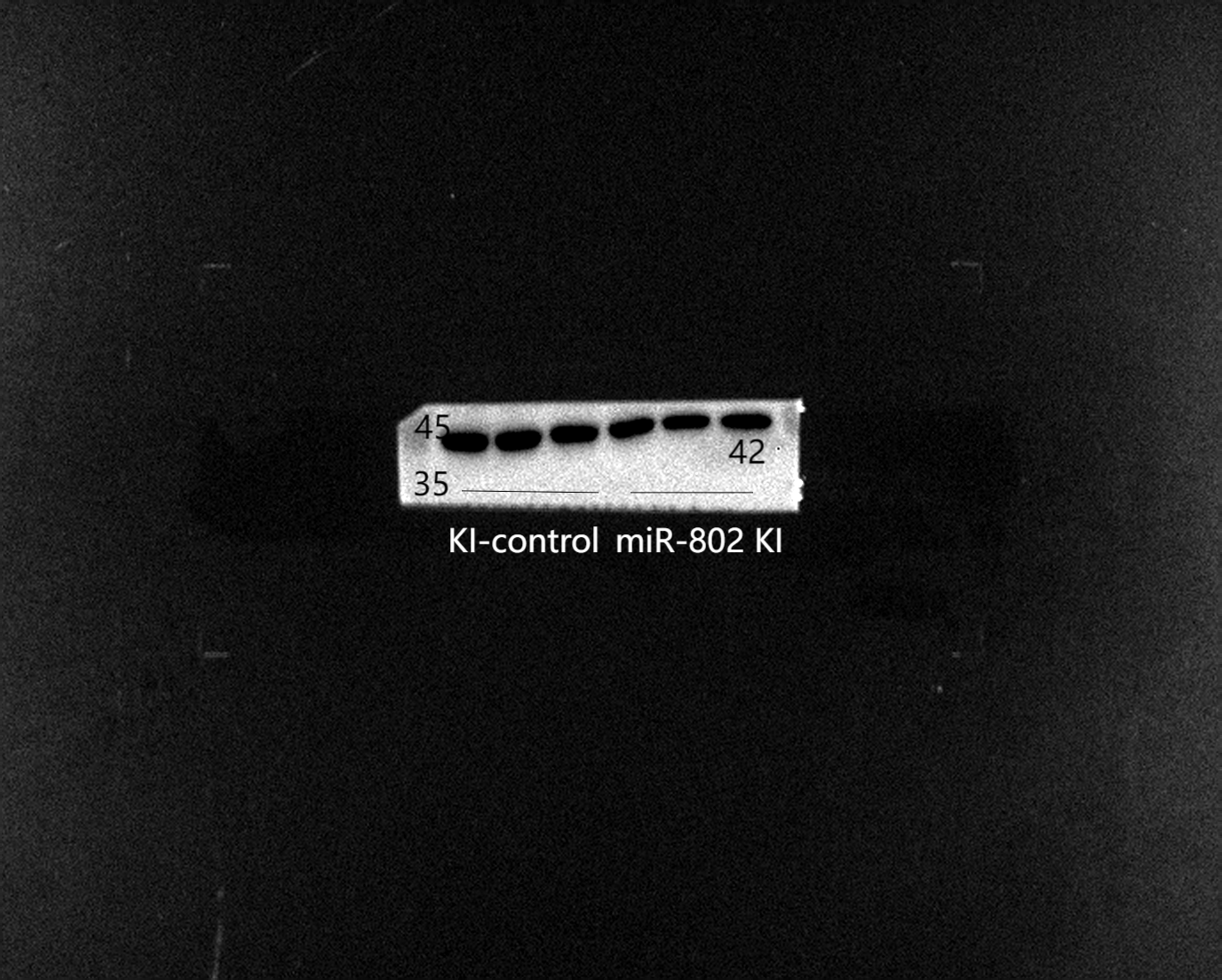

Supplement: Figure 7—source data 2. — The original files of the full raw unedited blots of m-SREBP1, P-SREBP1, and β-Actin in in the epiWAT of Mir802 KI mice (n=3). [file elife-99162-fig7-data2.zip › ╬▓-Actin.png]

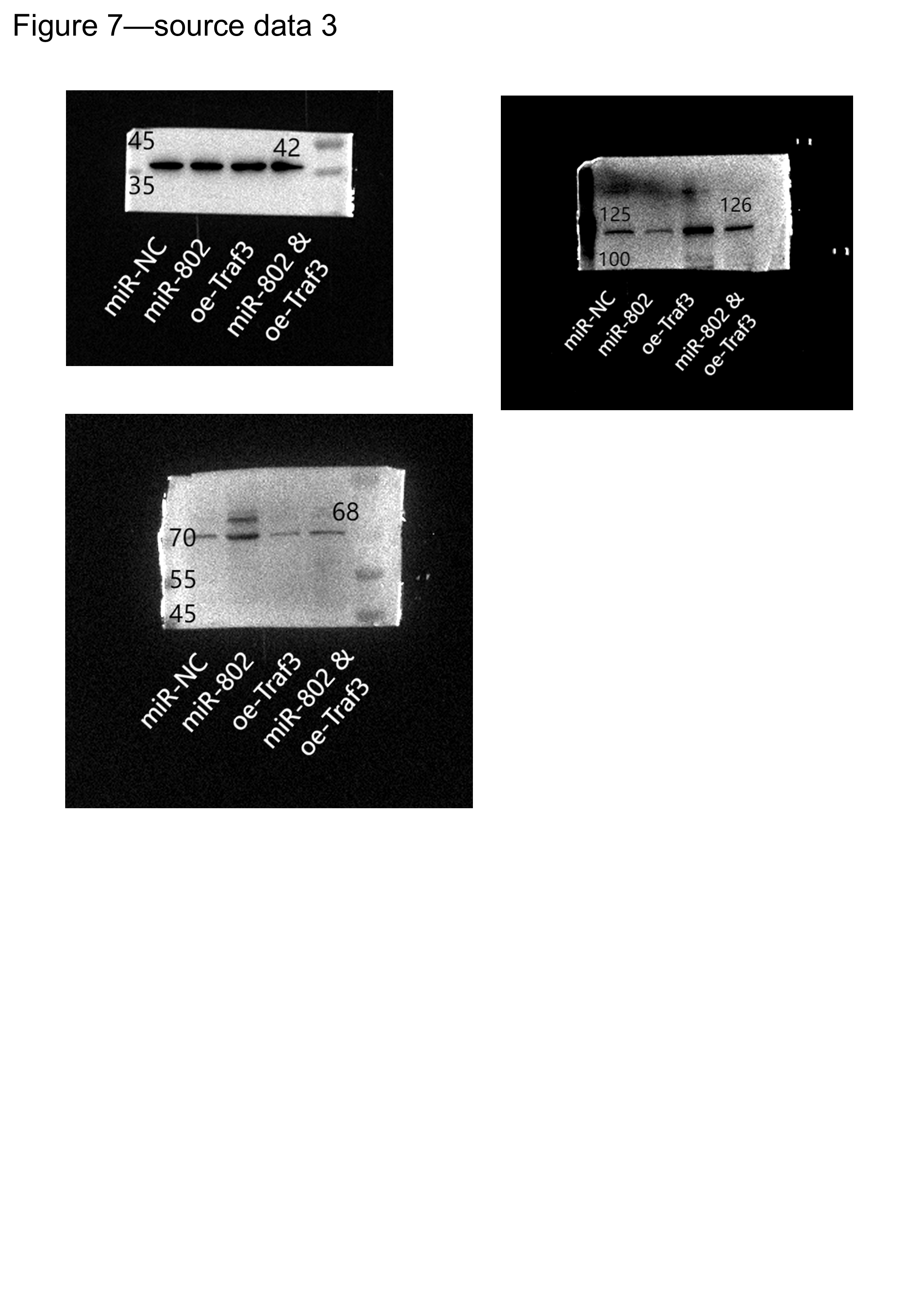

Supplement: Figure 7—source data 3. — The original files of the full raw unedited blots of m-SREBP1, P-SREBP1, and β-Actin in the 3T3-L1 cells. [file elife-99162-fig7-data3.zip › Figure 7ΓÇösource data 3.tif]

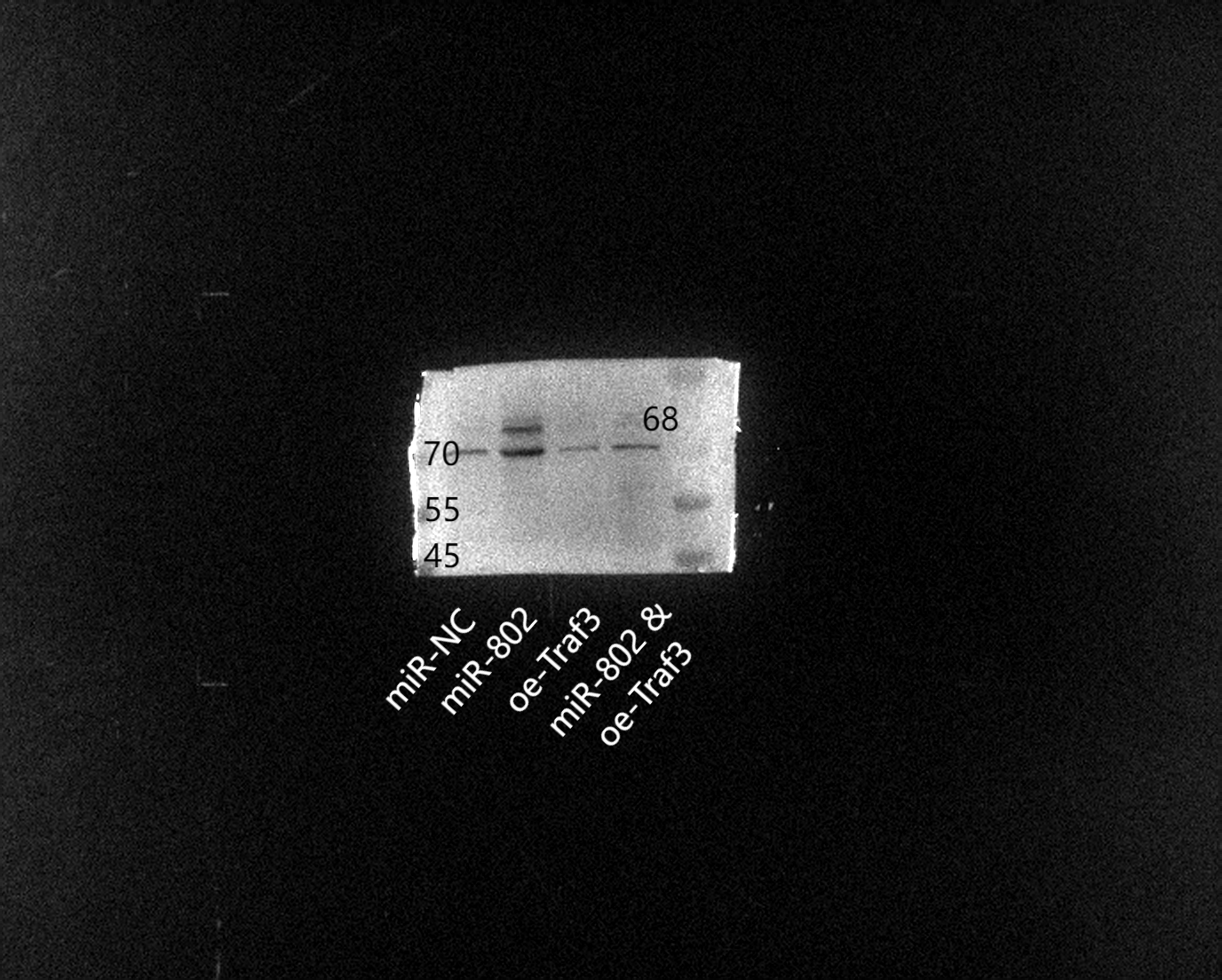

Supplement: Figure 7—source data 3. — The original files of the full raw unedited blots of m-SREBP1, P-SREBP1, and β-Actin in the 3T3-L1 cells. [file elife-99162-fig7-data3.zip › m-SREBP1-4.png]

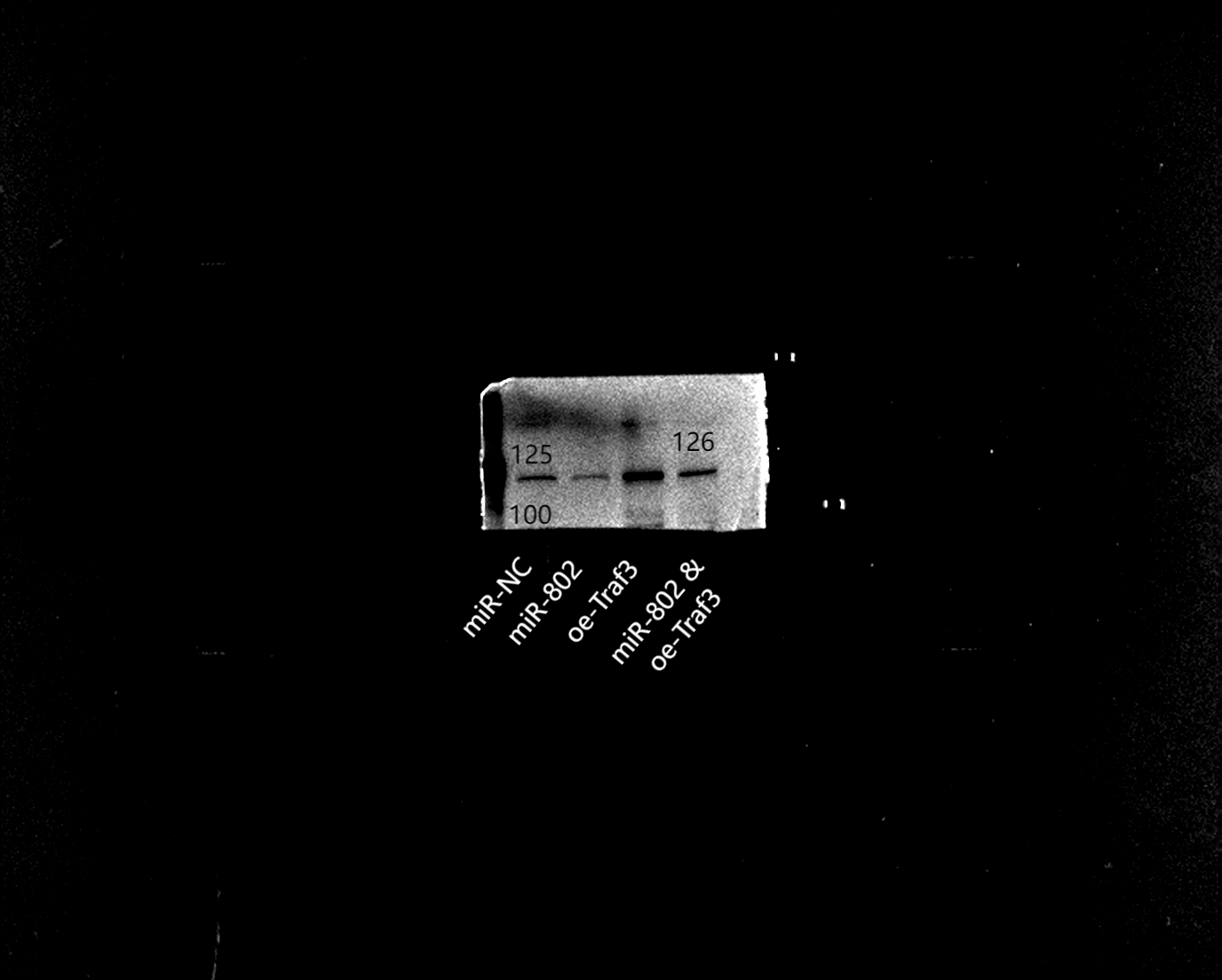

Supplement: Figure 7—source data 3. — The original files of the full raw unedited blots of m-SREBP1, P-SREBP1, and β-Actin in the 3T3-L1 cells. [file elife-99162-fig7-data3.zip › p-SREBP1-4.png]

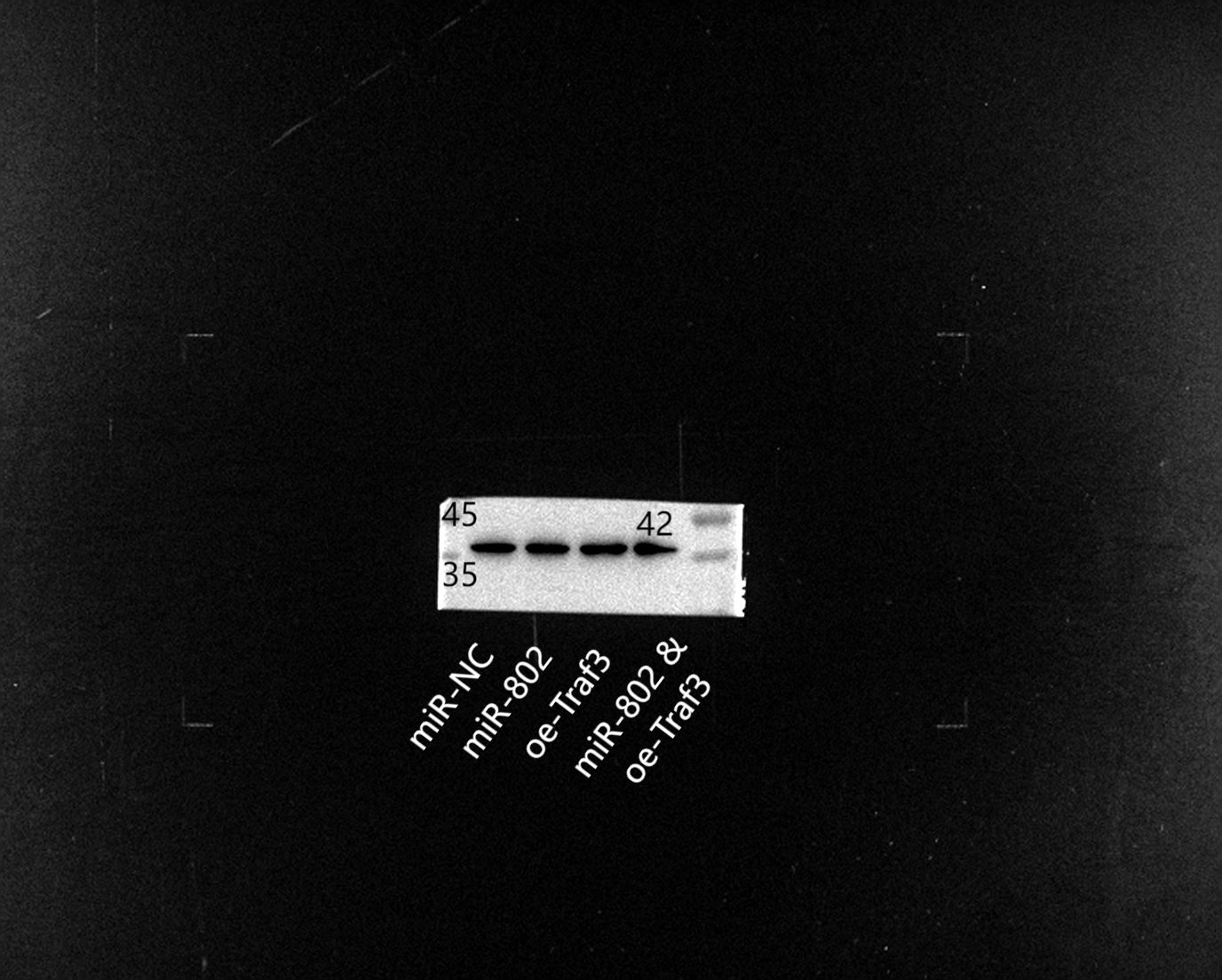

Supplement: Figure 7—source data 3. — The original files of the full raw unedited blots of m-SREBP1, P-SREBP1, and β-Actin in the 3T3-L1 cells. [file elife-99162-fig7-data3.zip › ╬▓-Actin.png]

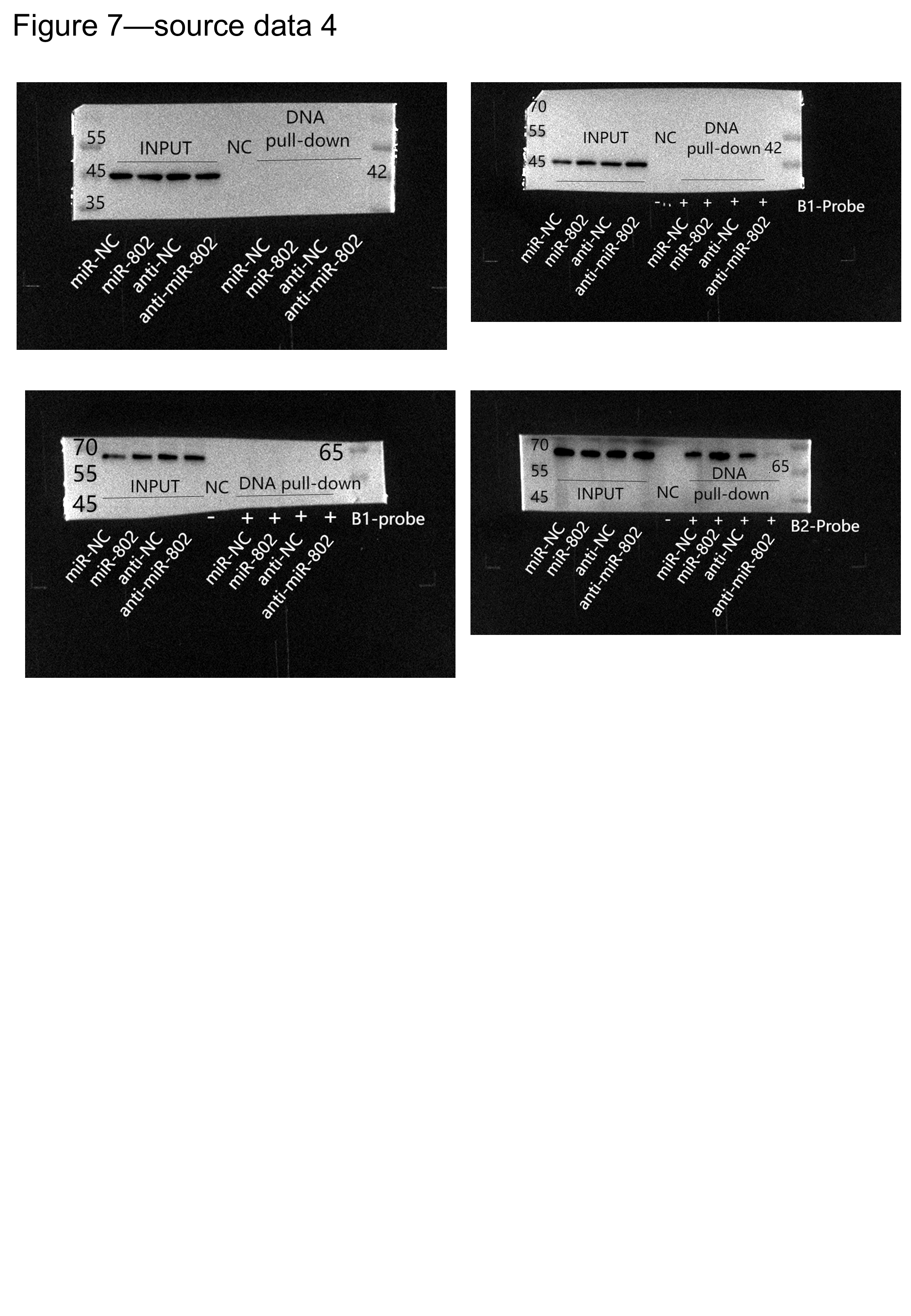

Supplement: Figure 7—source data 4. — The original files of the full raw unedited blots of p65 and β-Actin in 3T3-L1 cells transfected with Mir802 mimics or Mir802 inhibitor. [file elife-99162-fig7-data4.zip › Figure 7ΓÇösource data 4.tif]

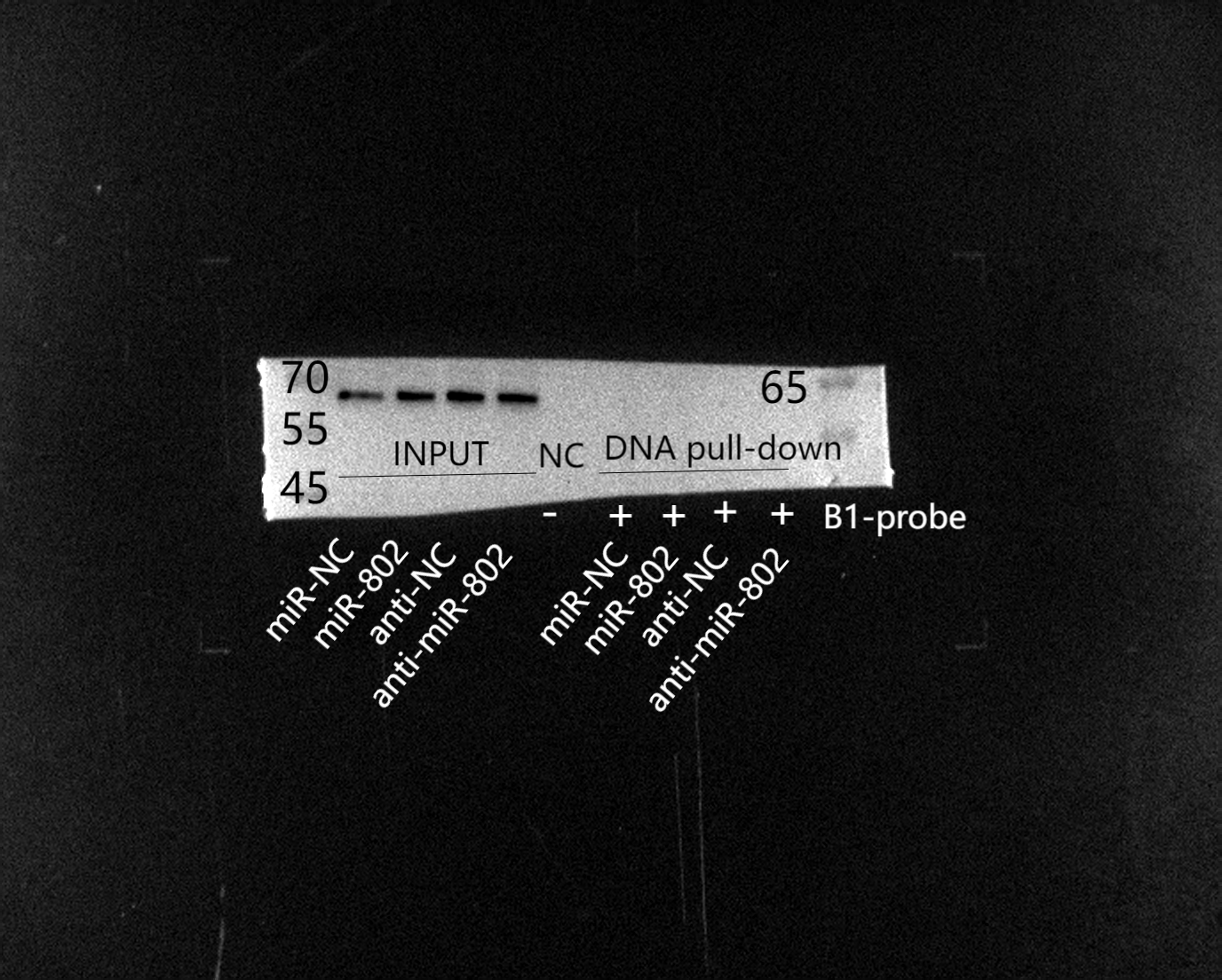

Supplement: Figure 7—source data 4. — The original files of the full raw unedited blots of p65 and β-Actin in 3T3-L1 cells transfected with Mir802 mimics or Mir802 inhibitor. [file elife-99162-fig7-data4.zip › B1-p65.png]

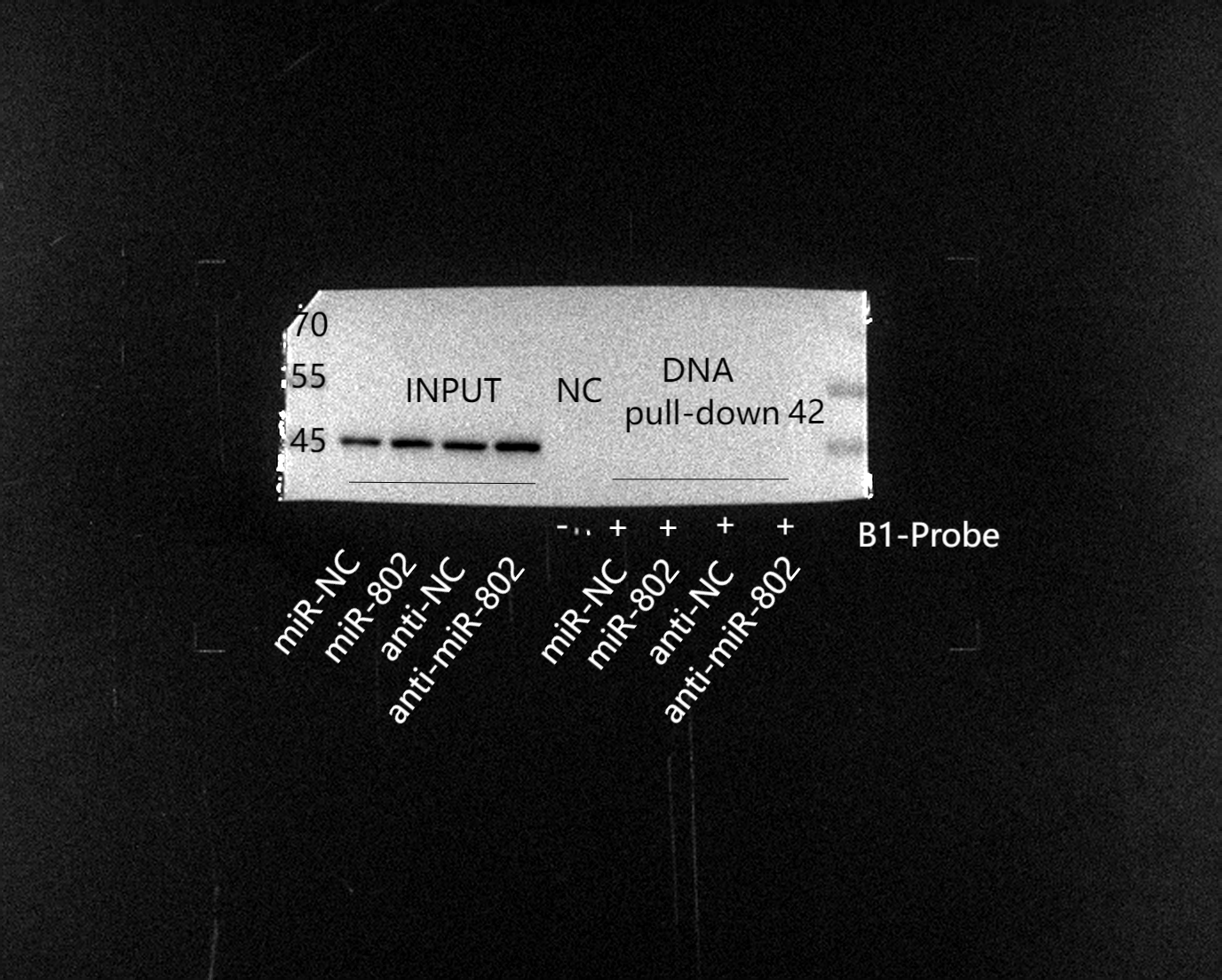

Supplement: Figure 7—source data 4. — The original files of the full raw unedited blots of p65 and β-Actin in 3T3-L1 cells transfected with Mir802 mimics or Mir802 inhibitor. [file elife-99162-fig7-data4.zip › B1-╬▓-Actin.png]

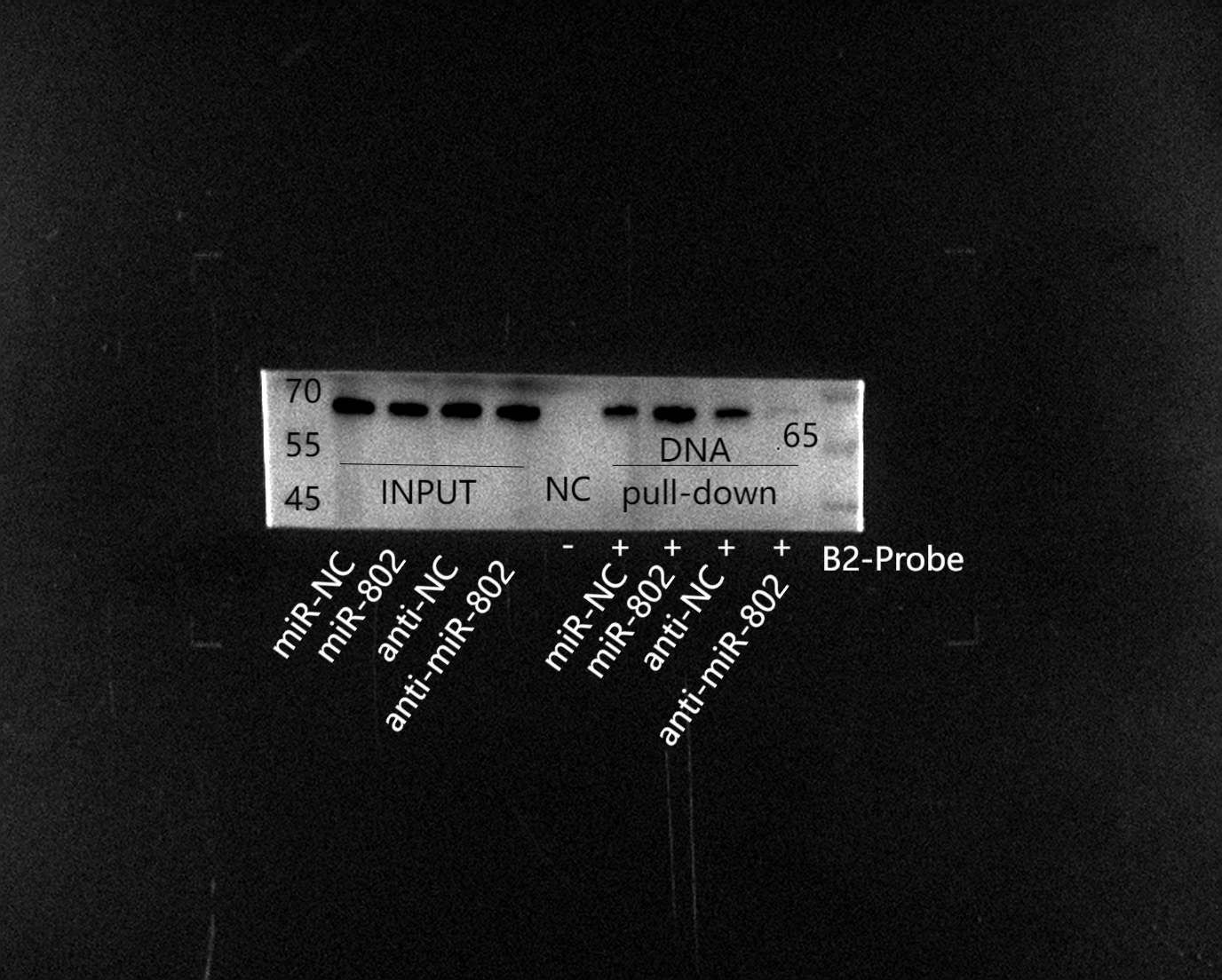

Supplement: Figure 7—source data 4. — The original files of the full raw unedited blots of p65 and β-Actin in 3T3-L1 cells transfected with Mir802 mimics or Mir802 inhibitor. [file elife-99162-fig7-data4.zip › B2-p65.png]

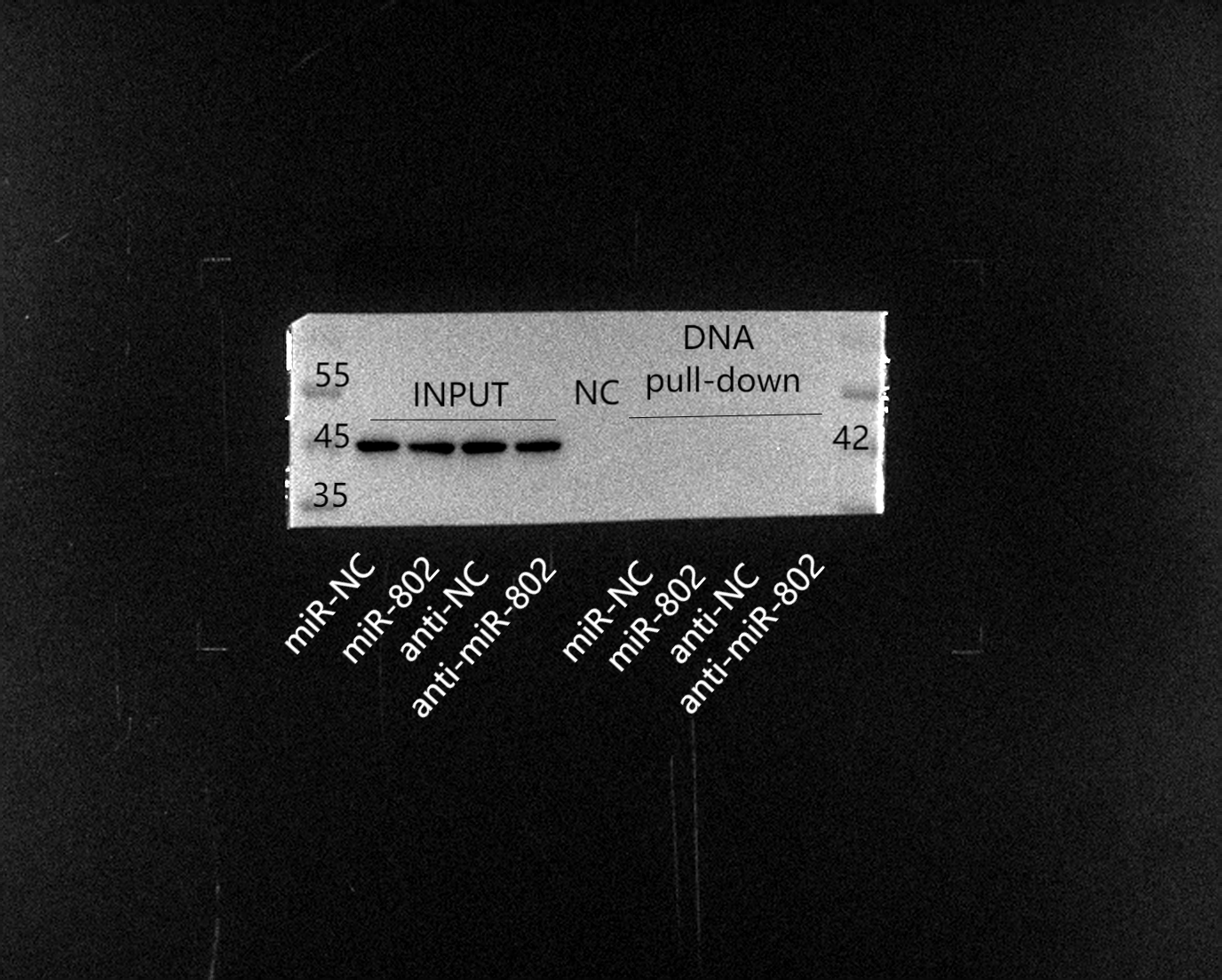

Supplement: Figure 7—source data 4. — The original files of the full raw unedited blots of p65 and β-Actin in 3T3-L1 cells transfected with Mir802 mimics or Mir802 inhibitor. [file elife-99162-fig7-data4.zip › B2-╬▓-Actin.png]

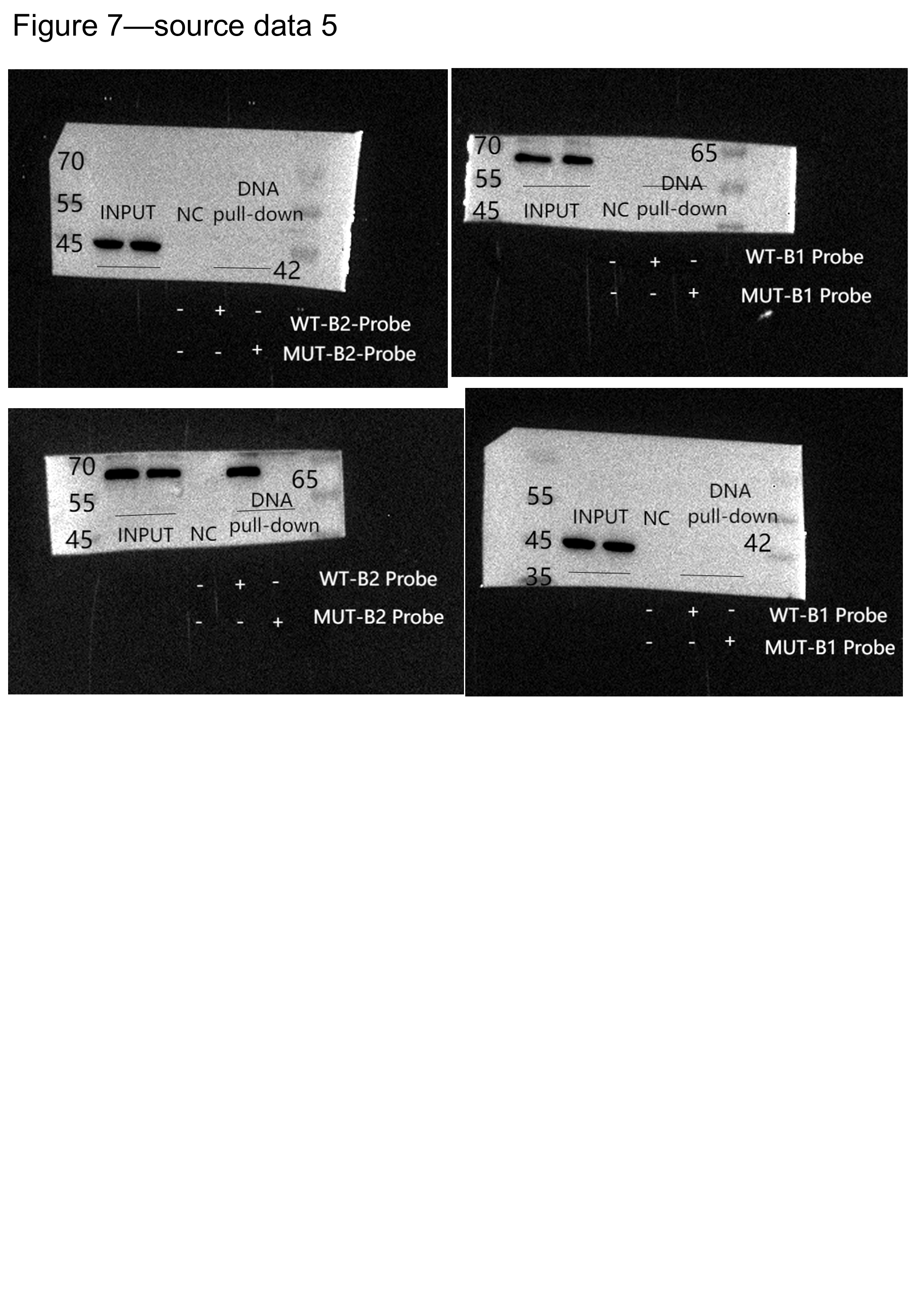

Supplement: Figure 7—source data 5. — The original files of the full raw unedited blots of p65 and β-Actin in 3T3-L1 cells stimulated with Rela plasmid for 48 hr. [file elife-99162-fig7-data5.zip › Figure 7ΓÇösource data 5.tif]

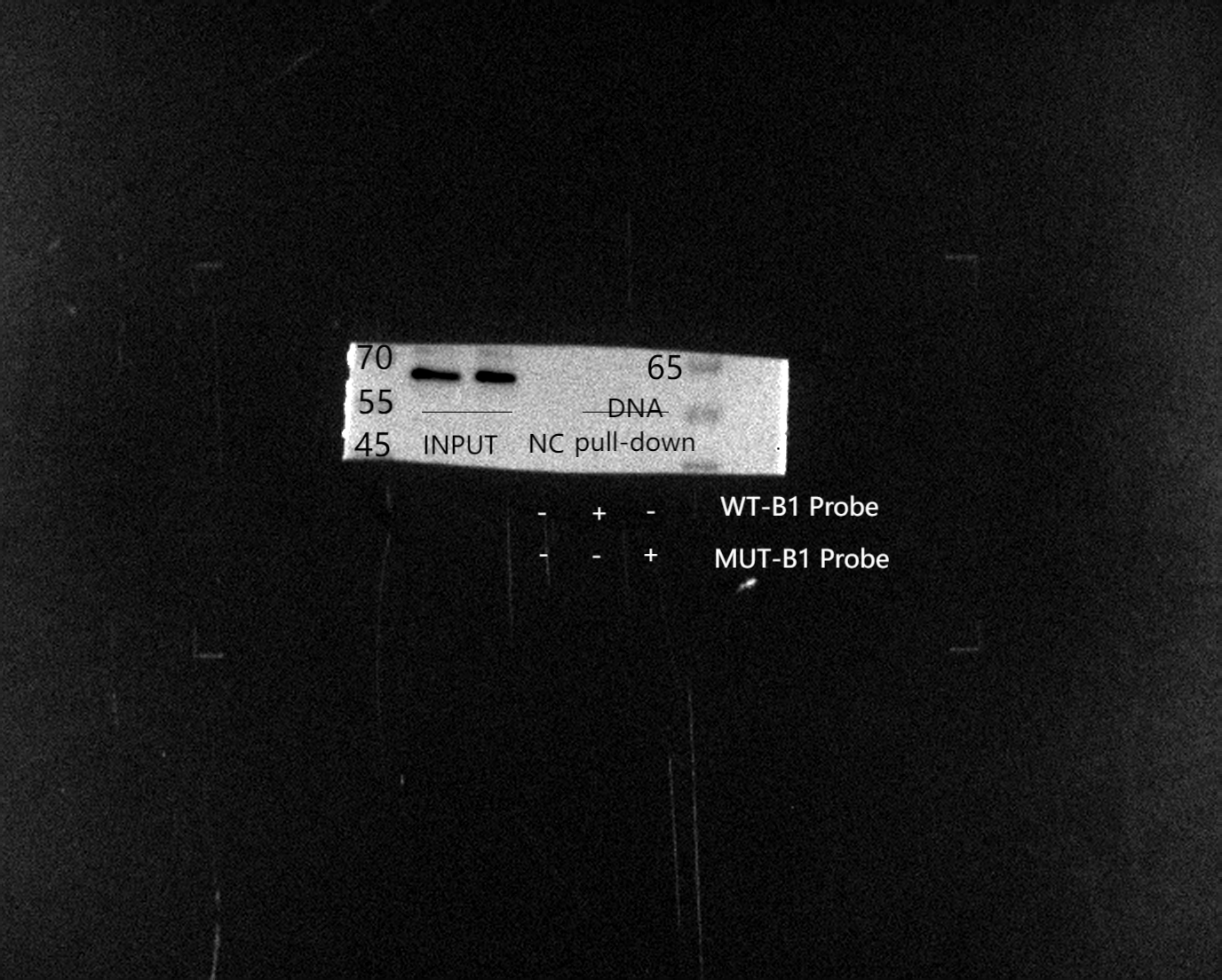

Supplement: Figure 7—source data 5. — The original files of the full raw unedited blots of p65 and β-Actin in 3T3-L1 cells stimulated with Rela plasmid for 48 hr. [file elife-99162-fig7-data5.zip › B1-p65.png]

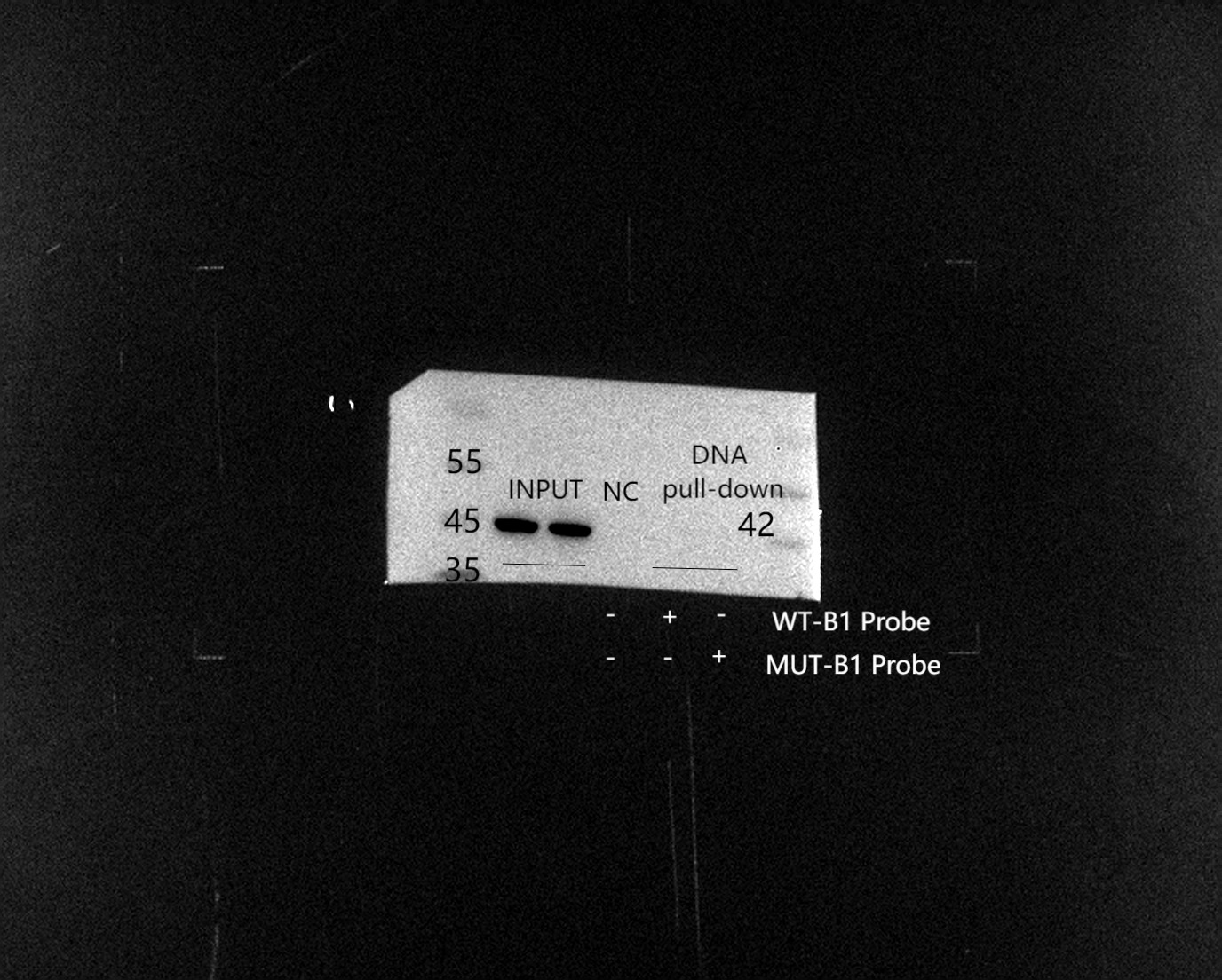

Supplement: Figure 7—source data 5. — The original files of the full raw unedited blots of p65 and β-Actin in 3T3-L1 cells stimulated with Rela plasmid for 48 hr. [file elife-99162-fig7-data5.zip › B1-╬▓-Actin.png]

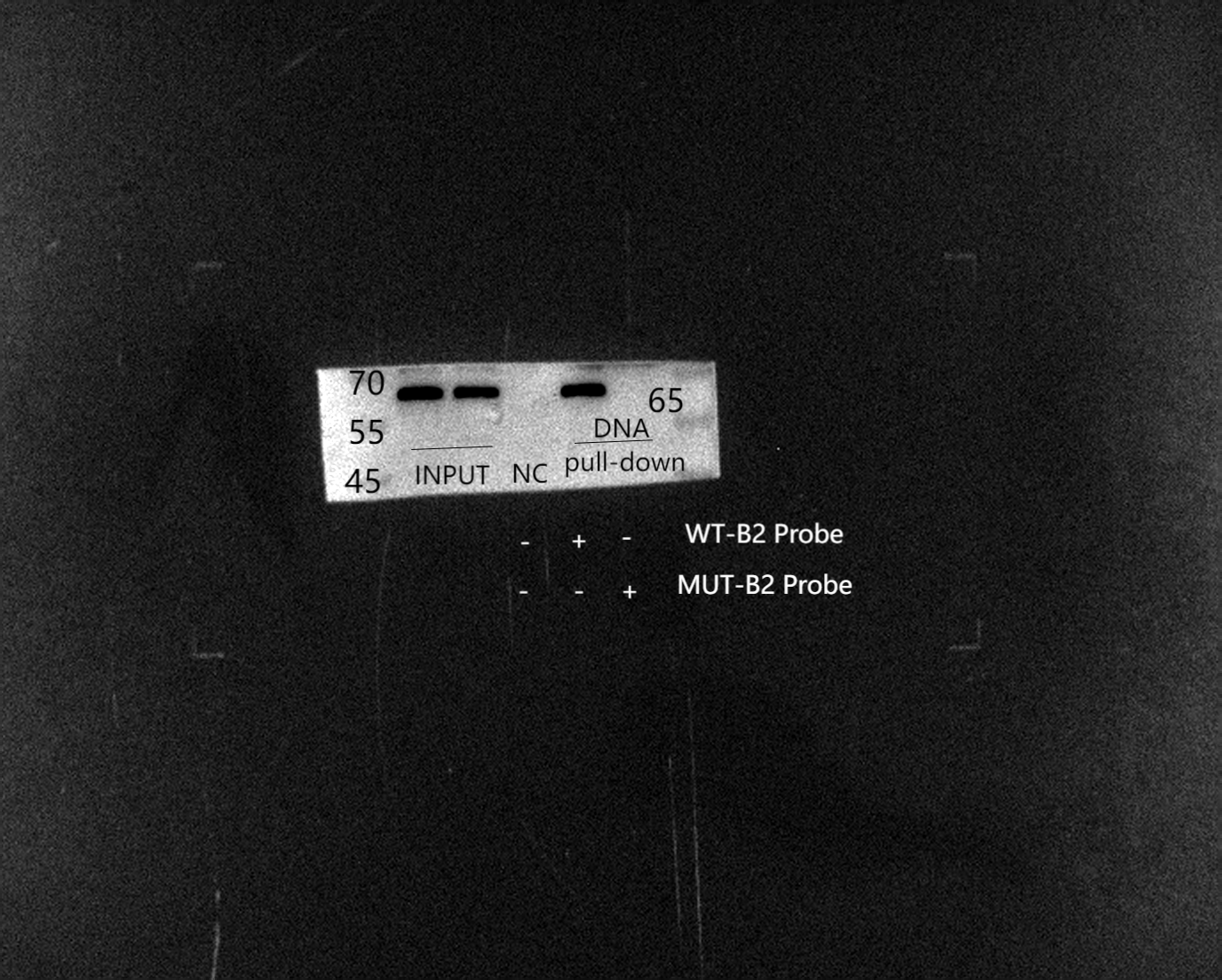

Supplement: Figure 7—source data 5. — The original files of the full raw unedited blots of p65 and β-Actin in 3T3-L1 cells stimulated with Rela plasmid for 48 hr. [file elife-99162-fig7-data5.zip › B2-p65.png]

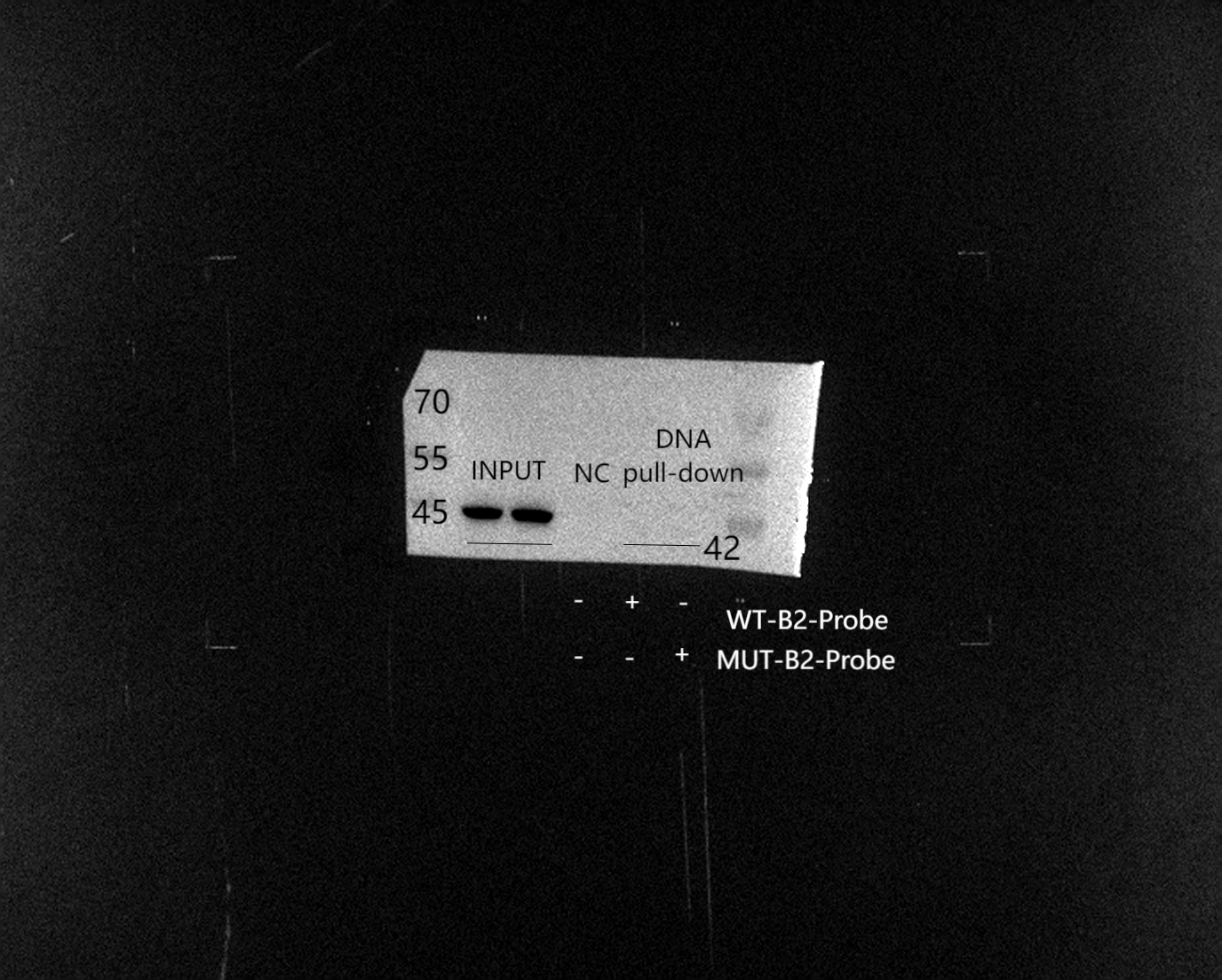

Supplement: Figure 7—source data 5. — The original files of the full raw unedited blots of p65 and β-Actin in 3T3-L1 cells stimulated with Rela plasmid for 48 hr. [file elife-99162-fig7-data5.zip › B2-╬▓-Actin.png]

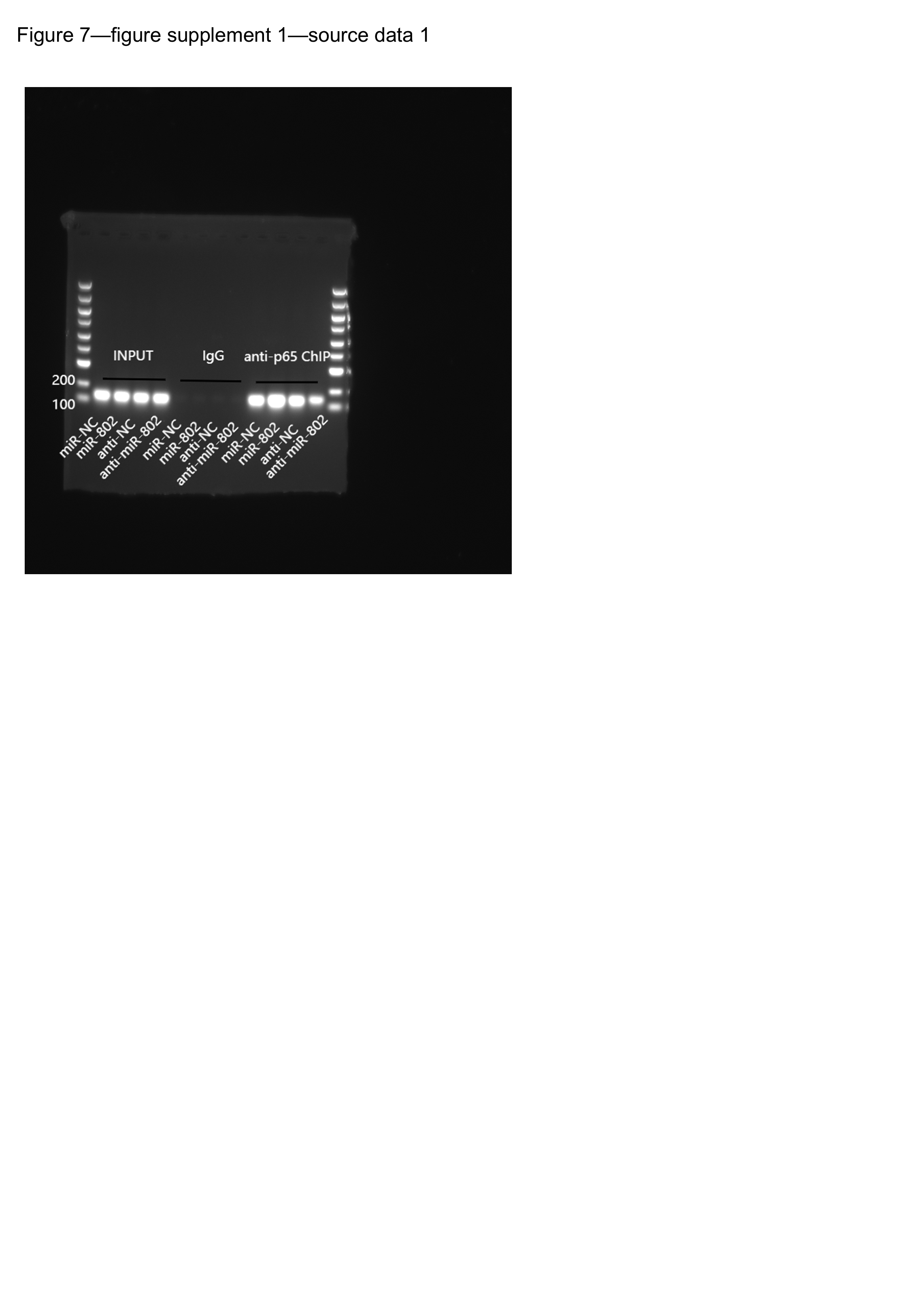

Supplement: Figure 7—figure supplement 1—source data 1. — The original files of the full raw unedited gels by ChIP-PCR experiments in the 3T3-L1 cells. [file elife-99162-fig7-figsupp1-data1.zip › Figure 7ΓÇöfigure supplement 1ΓÇösource data 1.tif]

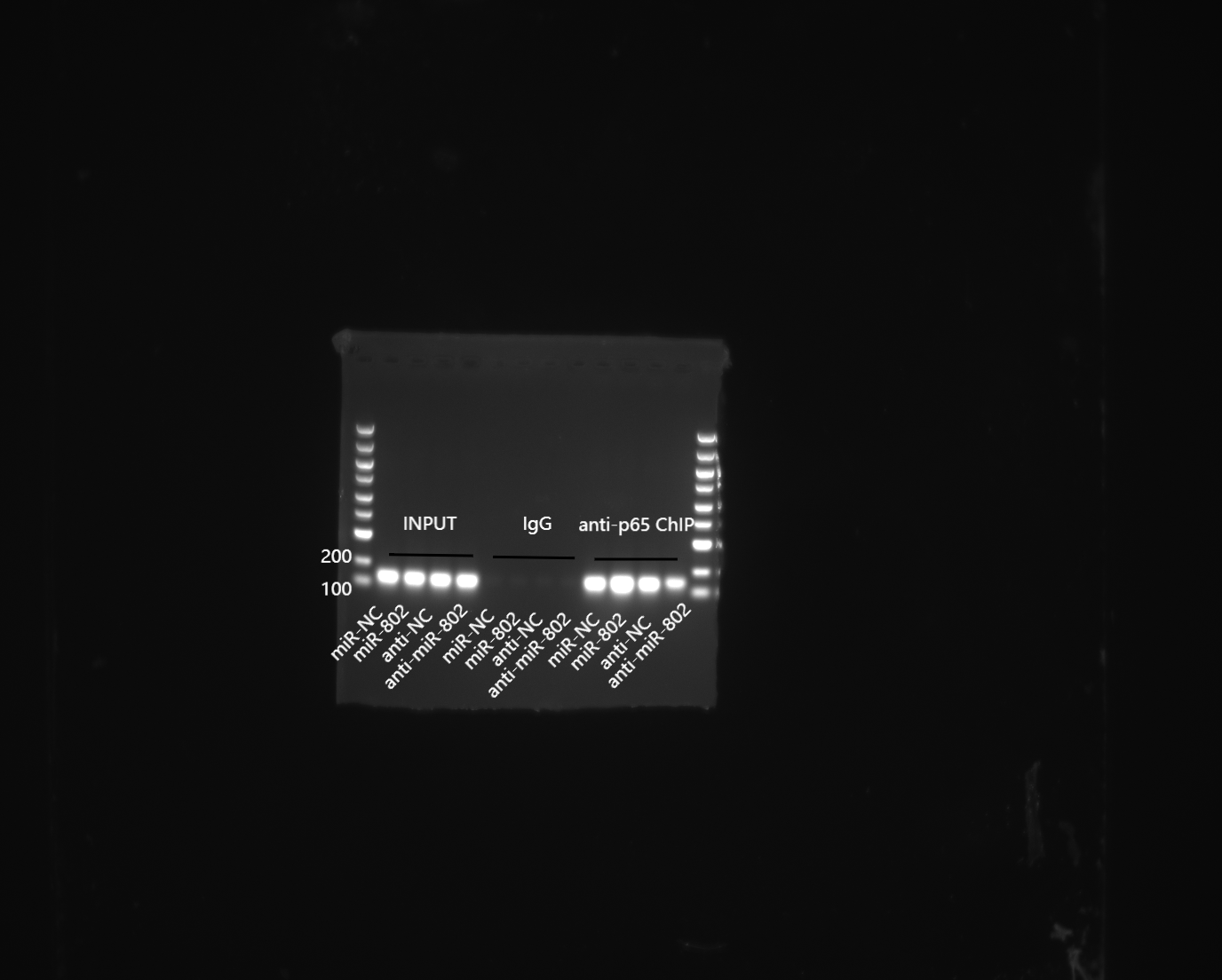

Supplement: Figure 7—figure supplement 1—source data 1. — The original files of the full raw unedited gels by ChIP-PCR experiments in the 3T3-L1 cells. [file elife-99162-fig7-figsupp1-data1.zip › CHIP-1.png]

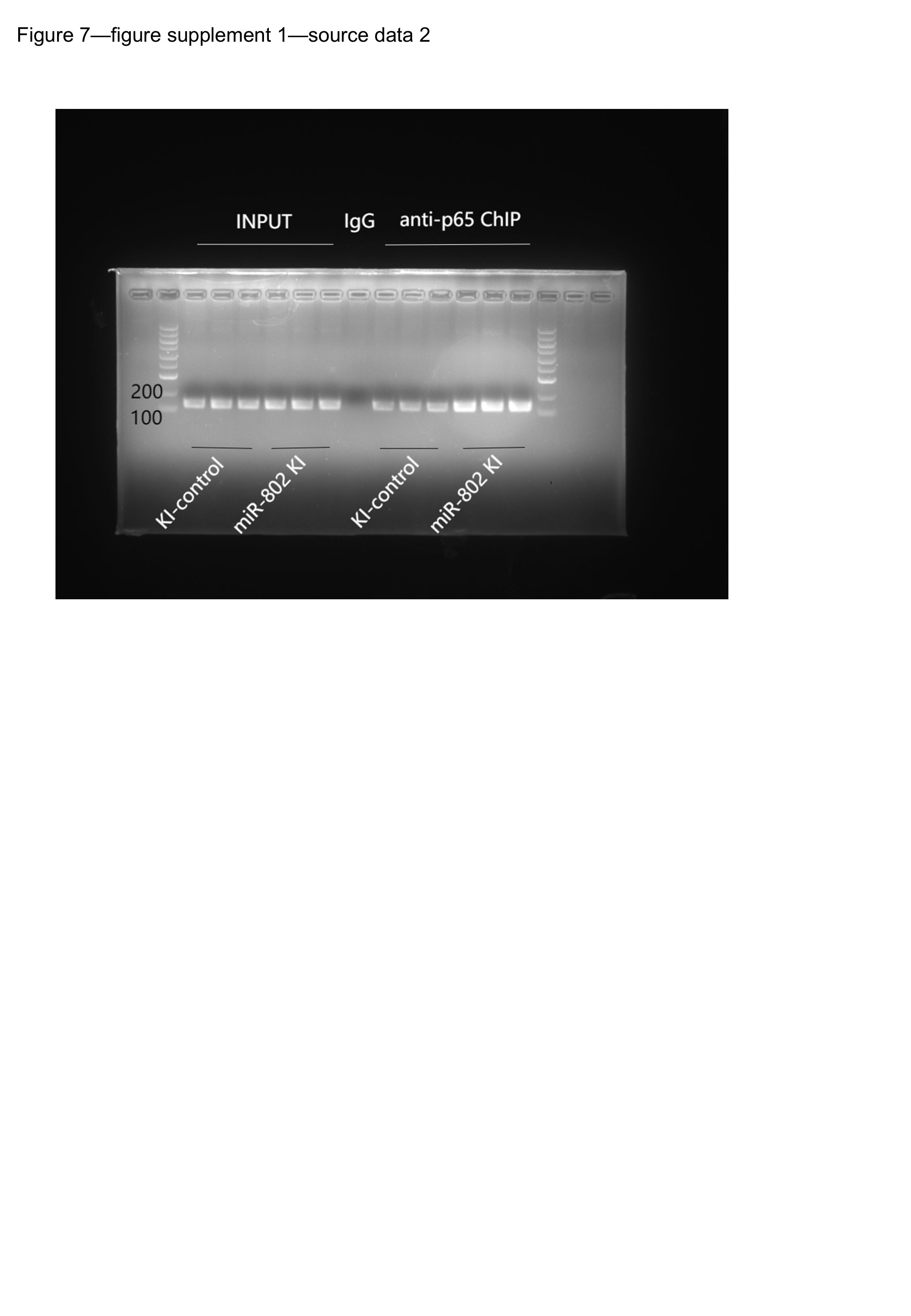

Supplement: Figure 7—figure supplement 1—source data 2. — The original files of the full raw unedited gels by ChIP-PCR experiments in the epiWAT of Mir802 KI mice (n=3). [file elife-99162-fig7-figsupp1-data2.zip › Figure 7ΓÇöfigure supplement 1ΓÇösource data 2.tif]

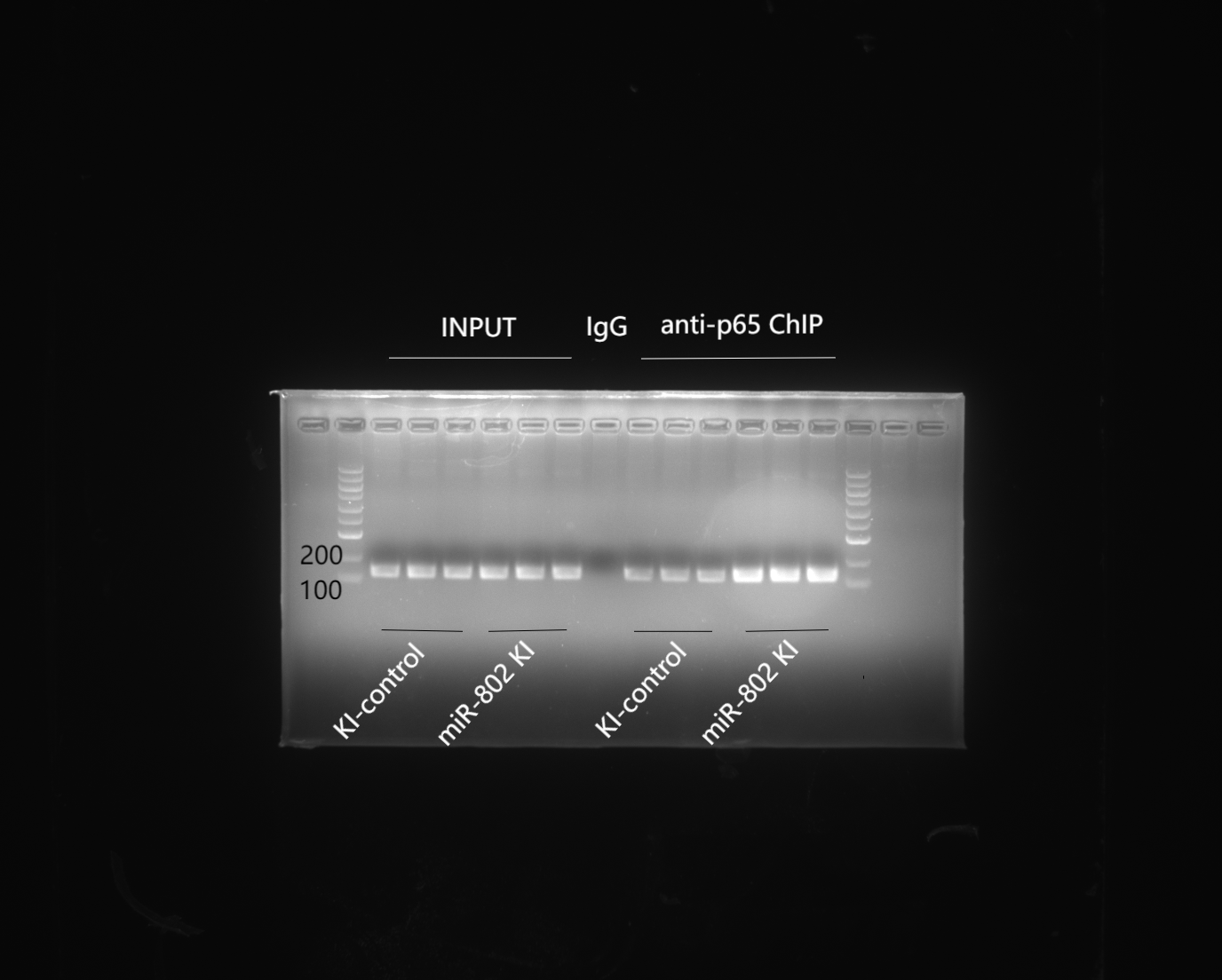

Supplement: Figure 7—figure supplement 1—source data 2. — The original files of the full raw unedited gels by ChIP-PCR experiments in the epiWAT of Mir802 KI mice (n=3). [file elife-99162-fig7-figsupp1-data2.zip › HFD-Chip.png]
