# Supplementary material for: Adipocyte microRNA-802 promotes adipose tissue inflammation and insulin resistance by modulating macrophages in obesity
Source: eLife. 2024 Nov 26;13:e99162. doi: 10.7554/eLife.99162 (PMC11651656; doi:10.7554/eLife.99162)
Supplement: Supplementary file 1. — (a) Clinical characteristics of the patients with obese patients and normal individuals. (b) RNA islolated from epiWAT of wide type mice and Mir802 KI mice, this table shows significantly changed mRNA (Log2 (FPKM (Mir802 KI/WT))≥1). (c) Primer sequences used for RT-PCR. (d) Oligo sequences used for shRNA. € The primers used in Real-time PCR (5’–3’). [file elife-99162-supp1.docx]

**Supplemental information**

**Supplementary Spreadsheets:**

**Supplementary file 1a**

**Supplementary file 1b**

**Supplementary file 1c**

**Supplementary file 1d**

**Supplementary file 1e**

**Supplementary file 1a Clinical characteristics of the patients with obese patients and** **normal individuals.**

|  | Obesity | Normal | Total |
| --- | --- | --- | --- |
| Number (male/female) | 70 (35/35) | 25(13/12) | 95(48/47) |
| Age (years) | 33.90±10.04 | 39.56±12.51 | 35.39±10.96 |
| HOMA-IR | 3.77±1.96 | 0.21±0.06 | 2.84±2.31 |
| Glucose (Mm) | 8.39±1.55 | 4.84±0.54 | 7.46±2.07 |
| BMI | 38.30±5.82 | 22.09±1.09 | 34.04±8.57 |

**Supplementary file 1b RNA islolated from epiWAT of wide type mice and *Mir802* KI mice, this table shows significantly changed mRNA (Log2 (FPKM (*Mir802* KI/WT)) ≥1).**

| GeneName | Ensembl_ID | log2FC | Pvalue | Style |
| --- | --- | --- | --- | --- |
| *Hoxa13* | ENSMUSG00000038203 | 9.651807 | 0.01351 | up |
| *Hoxd11* | ENSMUSG00000042499 | 6.658209 | 0.006495 | up |
| *Hoxc10* | ENSMUSG00000022484 | 4.693734 | 0.007714 | up |
| *En2* | ENSMUSG00000039095 | 4.595353 | 0.035864 | up |
| *srebp1a* | ENSMUSG00000020538 | 4.571764 | 0.009166 | up |
| *Pitx1* | ENSMUSG00000021506 | 4.341437 | 0.04596 | up |
| *Pou2f3* | ENSMUSG00000032015 | 4.150321 | 0.009676 | up |
| *Hoxa11* | ENSMUSG00000038210 | 3.980337 | 0.001692 | up |
| *Isl1* | ENSMUSG00000042258 | 3.860387 | 0.006858 | up |
| *Hand2* | ENSMUSG00000038193 | 3.725467 | 0.008763 | up |
| *Klf14* | ENSMUSG00000073209 | 3.49996 | 0.026366 | up |
| *Smad9* | ENSMUSG00000027796 | 2.841659 | 0.00793 | up |
| *Myc* | ENSMUSG00000022346 | 2.752626 | 0.000554 | up |
| *Hoxa10* | ENSMUSG00000000938 | 2.700145 | 0.011039 | up |
| *Mecom* | ENSMUSG00000027684 | 2.491253 | 0.000465 | up |
| *Wt1* | ENSMUSG00000016458 | 2.457969 | 0.008922 | up |
| *Ikzf3* | ENSMUSG00000018168 | 2.432464 | 0.001121 | up |
| *Id3* | ENSMUSG00000007872 | 2.411512 | 0.000256 | up |
| *Gata5* | ENSMUSG00000015627 | 2.323285 | 0.030167 | up |
| *Hsf3* | ENSMUSG00000045802 | 2.288822 | 0.006623 | up |
| *Atoh8* | ENSMUSG00000037621 | 2.25444 | 1.23E-09 | up |
| *Mxd3* | ENSMUSG00000021485 | 2.230772 | 0.002364 | up |
| *Tbx21* | ENSMUSG00000001444 | 2.182149 | 0.022158 | up |
| *Tcf15* | ENSMUSG00000068079 | 2.121915 | 0.026416 | up |
| *Fosb* | ENSMUSG00000003545 | 2.120021 | 0.009825 | up |
| *Nfatc2* | ENSMUSG00000027544 | 2.101469 | 0.000191 | up |
| *E2f2* | ENSMUSG00000018983 | 2.049752 | 0.003236 | up |
| *Hhex* | ENSMUSG00000024986 | 2.032146 | 0.000615 | up |
| *Meis2* | ENSMUSG00000027210 | 2.02506 | 0.001657 | up |
| *Klf2* | ENSMUSG00000055148 | 1.983177 | 0.000109 | up |
| *Fos* | ENSMUSG00000021250 | 1.962284 | 0.009234 | up |
| *Id4* | ENSMUSG00000021379 | 1.952154 | 0.00125 | up |
| *Id1* | ENSMUSG00000042745 | 1.947201 | 2.84E-05 | up |
| *Nr2f1* | ENSMUSG00000069171 | 1.893459 | 0.000532 | up |
| *Zfp532* | ENSMUSG00000042439 | 1.870363 | 0.010996 | up |
| *Batf2* | ENSMUSG00000039699 | 1.817167 | 0.005769 | up |
| *Tcf7l2* | ENSMUSG00000024985 | 1.785068 | 0.000178 | up |
| *Nfatc4* | ENSMUSG00000023411 | 1.733202 | 0.00053 | up |
| *Zbtb16* | ENSMUSG00000066687 | 1.700151 | 0.001574 | up |
| *Gm9791* | ENSMUSG00000044434 | 1.65468 | 0.049874 | up |
| *Meis1* | ENSMUSG00000020160 | 1.624023 | 8.06E-05 | up |
| *Stat5b* | ENSMUSG00000020919 | 1.588919 | 0.005036 | up |
| *Jdp2* | ENSMUSG00000034271 | 1.528232 | 0.017023 | up |
| *Meis3* | ENSMUSG00000041420 | 1.516706 | 0.000168 | up |
| *Hoxd10* | ENSMUSG00000050368 | 1.51041 | 0.037859 | up |
| *E2f1* | ENSMUSG00000027490 | 1.480872 | 0.007018 | up |
| *Sp100* | ENSMUSG00000026222 | 1.46691 | 0.039014 | up |
| *Stat5a* | ENSMUSG00000004043 | 1.427695 | 0.019569 | up |
| *Zfp811* | ENSMUSG00000055202 | 1.416561 | 0.003906 | up |
| *Carhsp1* | ENSMUSG00000008393 | 1.398672 | 0.004082 | up |
| *Prox1* | ENSMUSG00000010175 | 1.367738 | 0.031229 | up |
| *Fosl1* | ENSMUSG00000024912 | 1.339838 | 0.00617 | up |
| *Zeb1* | ENSMUSG00000024238 | 1.326205 | 0.015982 | up |
| *Prrx2* | ENSMUSG00000039476 | 1.321378 | 0.028109 | up |
| *Tcf4* | ENSMUSG00000053477 | 1.304501 | 0.013775 | up |
| *Klf6* | ENSMUSG00000000078 | 1.287591 | 4.22E-05 | up |
| *Nr2f2* | ENSMUSG00000030551 | 1.279173 | 0.019253 | up |
| *Tsc22d3* | ENSMUSG00000031431 | 1.27762 | 0.017579 | up |
| *Twist2* | ENSMUSG00000007805 | 1.268628 | 0.012384 | up |
| *Hic1* | ENSMUSG00000043099 | 1.267624 | 0.041162 | up |
| *Creb3l1* | ENSMUSG00000027230 | 1.266721 | 0.008765 | up |
| *Elk3* | ENSMUSG00000008398 | 1.233368 | 0.000975 | up |
| *Irf5* | ENSMUSG00000029771 | 1.206849 | 0.028752 | up |
| *E2f7* | ENSMUSG00000020185 | 1.179313 | 0.045124 | up |
| *Irf8* | ENSMUSG00000041515 | 1.157934 | 0.019144 | up |
| *Atf5* | ENSMUSG00000038539 | 1.145464 | 0.002427 | up |
| *Nfatc1* | ENSMUSG00000033016 | 1.145201 | 0.001501 | up |
| *Klf4* | ENSMUSG00000003032 | 1.130278 | 0.003696 | up |
| *Klf11* | ENSMUSG00000020653 | 1.121365 | 0.000285 | up |
| *Zfp808* | ENSMUSG00000074867 | 1.11147 | 0.00275 | up |
| *Nfia* | ENSMUSG00000028565 | 1.099911 | 0.005283 | up |
| *Mitf* | ENSMUSG00000035158 | 1.099293 | 0.004785 | up |
| *Gli3* | ENSMUSG00000021318 | 1.090808 | 0.006217 | up |
| *Jun* | ENSMUSG00000052684 | 1.03333 | 3.60E-14 | up |
| *Zfp874b* | ENSMUSG00000059839 | 1.002869 | 0.027519 | up |
| *E430018J23Rik* | ENSMUSG00000078580 | -1.00115 | 0.00899 | down |
| *Sox4* | ENSMUSG00000076431 | -1.01503 | 2.93E-06 | down |
| *9130019O22Rik* | ENSMUSG00000030823 | -1.02271 | 0.005096 | down |
| *Creb3l2* | ENSMUSG00000038648 | -1.02491 | 0.003621 | down |
| *Zfp458* | ENSMUSG00000055480 | -1.03078 | 0.006384 | down |
| *Zfp316* | ENSMUSG00000046658 | -1.03655 | 0.002049 | down |
| *Ncor2* | ENSMUSG00000029478 | -1.05786 | 0.001175 | down |
| *Cers6* | ENSMUSG00000027035 | -1.06075 | 0.006106 | down |
| *Litaf* | ENSMUSG00000022500 | -1.06154 | 5.26E-05 | down |
| *Pbx4* | ENSMUSG00000031860 | -1.08448 | 0.040166 | down |
| *Bach2* | ENSMUSG00000040270 | -1.08683 | 0.020266 | down |
| *Hoxb5* | ENSMUSG00000038700 | -1.09818 | 0.005408 | down |
| *Zfp1* | ENSMUSG00000055835 | -1.10056 | 1.39E-06 | down |
| *Zfp871* | ENSMUSG00000024298 | -1.10284 | 0.016568 | down |
| *Zfp612* | ENSMUSG00000044676 | -1.11372 | 0.000339 | down |
| *2810021J22Rik* | ENSMUSG00000020491 | -1.11666 | 0.000271 | down |
| *Sall2* | ENSMUSG00000049532 | -1.12955 | 0.004711 | down |
| *Sox13* | ENSMUSG00000070643 | -1.1318 | 0.007724 | down |
| *Plag1* | ENSMUSG00000003282 | -1.14004 | 0.026975 | down |
| *Hmga1* | ENSMUSG00000046711 | -1.16212 | 0.001131 | down |
| *Hoxd4* | ENSMUSG00000079277 | -1.164 | 0.001094 | down |
| *Mzf1* | ENSMUSG00000030380 | -1.19607 | 0.00022 | down |
| *Zfp213* | ENSMUSG00000071256 | -1.21342 | 0.004789 | down |
| *Etv1* | ENSMUSG00000004151 | -1.22741 | 0.000872 | down |
| *Bbx* | ENSMUSG00000022641 | -1.24668 | 6.30E-06 | down |
| *Hey2* | ENSMUSG00000019789 | -1.25209 | 0.021605 | down |
| *Sox12* | ENSMUSG00000051817 | -1.25921 | 0.006839 | down |
| *Vdr* | ENSMUSG00000022479 | -1.26995 | 0.005441 | down |
| *Hoxd8* | ENSMUSG00000027102 | -1.28756 | 0.000209 | down |
| *Erf* | ENSMUSG00000040857 | -1.31185 | 0.000252 | down |
| *Zfp105* | ENSMUSG00000057895 | -1.32159 | 0.000478 | down |
| *Gtf2ird1* | ENSMUSG00000023079 | -1.34195 | 0.007083 | down |
| *Sox9* | ENSMUSG00000000567 | -1.38557 | 0.014846 | down |
| *Hoxb3* | ENSMUSG00000048763 | -1.44753 | 9.20E-06 | down |
| *Zfp13* | ENSMUSG00000062012 | -1.47314 | 4.17E-05 | down |
| *Hoxb8* | ENSMUSG00000056648 | -1.48028 | 0.017419 | down |
| *Klf1* | ENSMUSG00000054191 | -1.48493 | 0.005377 | down |
| *Hoxb6* | ENSMUSG00000000690 | -1.50297 | 0.002964 | down |
| *Zfp189* | ENSMUSG00000039634 | -1.50583 | 0.004554 | down |
| *Runx1* | ENSMUSG00000022952 | -1.50718 | 0.011316 | down |
| *Trp63* | ENSMUSG00000022510 | -1.53182 | 2.35E-06 | down |
| *Sim1* | ENSMUSG00000019913 | -1.58265 | 0.005785 | down |
| *Tcf24* | ENSMUSG00000099032 | -1.59684 | 0.001423 | down |
| *Ebf4* | ENSMUSG00000053552 | -1.61954 | 0.000134 | down |
| *Snai3* | ENSMUSG00000006587 | -1.68222 | 0.047432 | down |
| *Bhlhe40* | ENSMUSG00000030103 | -1.68666 | 0.015231 | down |
| *Gm6104* | ENSMUSG00000062588 | -1.69835 | 0.01131 | down |
| *Bhlhe41* | ENSMUSG00000030256 | -1.73093 | 0.003425 | down |
| *Zfp618* | ENSMUSG00000028358 | -1.73194 | 0.000579 | down |
| *Hmgb3* | ENSMUSG00000015217 | -1.76117 | 0.000174 | down |
| *Hsf5* | ENSMUSG00000070345 | -1.76479 | 0.006223 | down |
| *Trp73* | ENSMUSG00000029026 | -1.79092 | 0.001251 | down |
| *Hoxb1* | ENSMUSG00000018973 | -1.79188 | 0.005758 | down |
| *Hoxb7* | ENSMUSG00000038721 | -1.80942 | 1.85E-06 | down |
| *Prdm6* | ENSMUSG00000069378 | -1.88098 | 0.02543 | down |
| *Gm20939* | ENSMUSG00000095193 | -1.91425 | 0.00028 | down |
| *Pax2* | ENSMUSG00000004231 | -1.94359 | 0.009466 | down |
| *Emx2* | ENSMUSG00000043969 | -1.94656 | 4.23E-09 | down |
| *Zkscan2* | ENSMUSG00000030757 | -1.96299 | 0.015069 | down |
| *Nkx3-1* | ENSMUSG00000022061 | -2.03865 | 0.011156 | down |
| *Pax9* | ENSMUSG00000001497 | -2.05567 | 0.022889 | down |
| *Lef1* | ENSMUSG00000027985 | -2.06109 | 0.003616 | down |
| *Scx* | ENSMUSG00000034161 | -2.10484 | 7.74E-09 | down |
| *Dlx3* | ENSMUSG00000001510 | -2.1411 | 0.011598 | down |
| *Tcf7* | ENSMUSG00000000782 | -2.14481 | 0.001787 | down |
| *Aire* | ENSMUSG00000000731 | -2.16065 | 0.019215 | down |
| *Etv4* | ENSMUSG00000017724 | -2.19505 | 0.023468 | down |
| *Hoxd1* | ENSMUSG00000042448 | -2.20092 | 0.017915 | down |
| *Hnf1a* | ENSMUSG00000029556 | -2.20866 | 0.031263 | down |
| *Zfp941* | ENSMUSG00000060314 | -2.22479 | 0.004722 | down |
| *Nr1h5* | ENSMUSG00000048938 | -2.36293 | 0.014714 | down |
| *Myb* | ENSMUSG00000019982 | -2.39568 | 1.09E-09 | down |
| *Tfcp2l1* | ENSMUSG00000026380 | -2.42119 | 7.41E-13 | down |
| *Bhlhe22* | ENSMUSG00000025128 | -2.45754 | 0.006164 | down |
| *Esrrg* | ENSMUSG00000026610 | -2.46386 | 3.38E-06 | down |
| *Glis1* | ENSMUSG00000034762 | -2.46484 | 0.001618 | down |
| *Pou3f1* | ENSMUSG00000090125 | -2.51799 | 0.001973 | down |
| *Tox3* | ENSMUSG00000043668 | -2.52657 | 1.02E-05 | down |
| *Nfe2l3* | ENSMUSG00000029832 | -2.5395 | 0.000503 | down |
| *Sall1* | ENSMUSG00000031665 | -2.55742 | 1.72E-05 | down |
| *Dbx2* | ENSMUSG00000045608 | -2.56639 | 0.00521 | down |
| *Sp5* | ENSMUSG00000075304 | -2.59169 | 3.22E-06 | down |
| *Esr2* | ENSMUSG00000021055 | -2.62893 | 0.003077 | down |
| *Hes2* | ENSMUSG00000028940 | -2.67322 | 0.001086 | down |
| *Esrrb* | ENSMUSG00000021255 | -2.72137 | 0.000244 | down |
| *Pitx2* | ENSMUSG00000028023 | -2.73104 | 0.024335 | down |
| *L3mbtl1* | ENSMUSG00000035576 | -2.76041 | 0.004755 | down |
| *Pax8* | ENSMUSG00000026976 | -2.82564 | 0.005089 | down |
| *Lhx1* | ENSMUSG00000018698 | -2.93839 | 0.001171 | down |
| *Pou3f4* | ENSMUSG00000056854 | -3.0065 | 0.03371 | down |
| *Foxi1* | ENSMUSG00000047861 | -3.03271 | 0.000474 | down |
| *Evx1* | ENSMUSG00000005503 | -3.05044 | 4.01E-05 | down |
| *Gfi1b* | ENSMUSG00000026815 | -3.11227 | 7.72E-05 | down |
| *Zfp541* | ENSMUSG00000078796 | -3.13686 | 0.000672 | down |
| *Mycn* | ENSMUSG00000037169 | -3.14579 | 5.11E-07 | down |
| *Arnt2* | ENSMUSG00000015709 | -3.14871 | 0.00319 | down |
| *Tcfl5* | ENSMUSG00000038932 | -3.16475 | 0.001862 | down |
| *Mesp2* | ENSMUSG00000030543 | -3.30298 | 0.00088 | down |
| *Foxj1* | ENSMUSG00000034227 | -3.45902 | 2.02E-05 | down |
| *Lhx4* | ENSMUSG00000026468 | -3.47487 | 4.04E-05 | down |
| *Ascl2* | ENSMUSG00000009248 | -3.48941 | 0.000492 | down |
| *Tfap2b* | ENSMUSG00000025927 | -3.51738 | 1.43E-14 | down |
| *Hnf4a* | ENSMUSG00000017950 | -3.58372 | 0.000256 | down |
| *Gm17067* | ENSMUSG00000091594 | -3.60455 | 0.016235 | down |
| *Scrt2* | ENSMUSG00000060257 | -3.63211 | 2.55E-06 | down |
| *Zfp488* | ENSMUSG00000044519 | -3.63919 | 0.04901 | down |
| *Nr1i2* | ENSMUSG00000022809 | -3.79131 | 0.031518 | down |
| *Zfp474* | ENSMUSG00000046886 | -3.88809 | 8.20E-06 | down |
| *Hmgb4* | ENSMUSG00000048686 | -4.01943 | 4.76E-05 | down |
| *Gm6871* | ENSMUSG00000090744 | -4.02229 | 8.57E-07 | down |
| *Nkx2-6* | ENSMUSG00000044186 | -4.04249 | 0.015039 | down |
| *Tbx22* | ENSMUSG00000031241 | -4.19646 | 3.97E-08 | down |
| *Traf3* | ENSMUSG00000021277 | -4.20009 | 3.99E-05 | down |
| *Rnf138rt1* | ENSMUSG00000083695 | -4.55924 | 0.003643 | down |
| *Cdx2* | ENSMUSG00000029646 | -4.59039 | 0.008581 | down |
| *L3mbtl4* | ENSMUSG00000041565 | -5.27874 | 0.006449 | down |

**Supplementary file 1c Primer sequences used for RT-PCR.**

| Gene | Forward strand (5’-3’) | Reverse strand (5’-3’) |
| --- | --- | --- |
| oe*-Traf3* | CCGCTCGAGATGGAGTCAAGCAAAAAG | CGCGGATCCTCAGGGGTCAGGCAGATC |
| oe*-Srebp1* | GGAATTCCATGGACGAGCTGGCCTTCGGTGAGG | TTGCGGCCGCAATAGCTGGAAGTGACGGTGGTTCCG |
| oe*-Rela* | GGAATTCCATGGACGATCTGTTTCCCCT | TTGCGGCCGCAAGGAGCTGATCTGACTCAAAAGA |
| oe*-RelB* | GGAATTCCATGCCGAGTCGCCGCGCTG | TTGCGGCCGCAACGTGGCTTCAGGCCCTGGAGAT |
| *Traf3-*WT | TCGAGCATCCTAAAATTCAAGAGTGCAATCTTGTTTCAAATATAGTATATT | CTAGAATATACTATATTTGAAACAAGATTGCACTCTTGAATTTTAGGATGC |
| *Traf3-*mut | TCGAGGTTAGTGACACTACTTCATCCGTCACATGAGAGGGACAATGCTCAT | CTAGATGAGCATTGTCCCTCTCATAAGGCAGTGTGAAGTAGTGTCACTAAC C |
| *Rela-*WT | AACTGGGTTAGGTAGGGAATGCTTTCTCTGGGGAGCCTG | GCAGGCTCCCCAGAGAAAGCATTCCCTACCTAACCCAGTT |
| *Rela-*mut | AACTGGGTTAGGTCTTTGGCAAGTTCTCTGGGGAGCCTGC | GCAGGCTCCCCAGAGAACTTGCCAAAGACCTAACCCAGTT |
| *Adipoq-*Cre | ACGGACAGAAGCATTTTCCA | GGATGTGCCATGTGAGTCTG |
| *Mir802* KO  allele | GCATCGCATTGTCTGAGTAGGTG | AGTGCAGTTACCCGTCACCA |
| *Mir802* KI allele | CTAGAGCCTCTGCTAACCATGTTC | AAGAGCCTTCAGTAAAGAGCAGG |

**Supplementary file 1d Oligo sequences used for shRNA.**

| Gene | Forward strand (5’-3’) | Reverse strand (5’-3’) |
| --- | --- | --- |
| Sh*-Traf3-*1 | GATCCGAATGAAAGTGTTGAGAAA TTCAAGAGATTTCTCAACACTTTCATTC TTTTTTG | AATTCAAAAAAGAATGAAAGTGTTGAGAAATCTCTTGAATTTCTCAACACTTTCATTC G |
| Sh*-Traf3-*2 | GATCCCGGTGGAAGACAAGTACAA TTCAAGAGATTGTACTTGTCTTCCACCG TTTTTTG | AATTCAAAAAACGGTGGAAGACAAGTACAATCTCTTGAATTGTACTTGTCTTCCACCG G |
| Sh*-Traf3-*3 | GATCCGGAAGATCCGTGACTACAATTCAAGAGATTGTAGTCACGGATCTTCC TTTTTTG | AATTCAAAAAAGGAAGATCCGTGACTACAATCTCTTGAATTGTAGTCACGGATCTTCC G |
| Sh*- Rela-*1 | GATCCGCCTCATCCACATGAACTTGT TTCAAGAGAACAAGTTCATGTGGATGAGGCTTTTTTG | AATTCAAAAAAGCCTCATCCACATGAACTTGTTCTCTTGAAACAAGTTCATGTGGATGAGGC G |
| Sh- *Rela -*2 | GATCCGCGAATCCAGACCAACAATAA TTCAAGAGATTATTGTTGGTCTGGATTCGC TTTTTTG | AATTCAAAAAAGCGAATCCAGACCAACAATAATCTCTTGAATTATTGTTGGTCTGGATTCGC G |
| Sh*- Rela* -3 | GATCCGGACCTATGAGACCTTCAAGATTCAAGAGATCTTGAAGGTCTCATAGGTCCTTTTTTG | AATTCAAAAAAGGACCTATGAGACCTTCAAGATCTCTTGAATCTTGAAGGTCTCATAGGTCC G |
| Sh*-Relb-*1 | GATCCGCTACGGTGTGGACAAGAATTCAAGAGATTCTTGTCCACACCGTAGC TTTTTTG | AATTCAAAAAAGCTACGGTGTGGACAAGAATCTCTTGAATTCTTGTCCACACCGTAGC G |
| Sh*-Relb*-2 | GATCCGGATTTGCCGAATCAACAATTCAAGAGATTGTTGATTCGGCAAATCC TTTTTTG | AATTCAAAAAAGGATTTGCCGAATCAACAATCTCTTGAATTGTTGATTCGGCAAATCC G |
| Sh*-Relb*-3 | GATCCCAGAAATCATCGACGAATATTCAAGAGATATTCGTCGATGATTTCTG TTTTTTG | AATTCAAAAAACAGAAATCATCGACGAATATCTCTTGAATATTCGTCGATGATTTCTG G |
| Sh*-Srebp1-*1 | GATCCGCAGCAGCAAGCACTTCAATTCAAGAGATTGAAGTGCTTGCTGCTGC TTTTTTG | AATTCAAAAAAGCAGCAGCAAGCACTTCAATCTCTTGAATTGAAGTGCTTGCTGCTGC G |
| Sh-*Srebp1*-2 | GATCCGCTACAGCAGCTATTCCAATTCAAGAGATTGGAATAGCTGCTGTAGC TTTTTTG | AATTCAAAAAAGCTACAGCAGCTATTCCAATCTCTTGAATTGGAATAGCTGCTGTAGC G |
| Sh*-Srebp1*-3 | GATCCGGGTCAACAGCAACTCCAA TTCAAGAGATTGGAGTTGCTGTTGACCCTTTTTTG | AATTCAAAAAAGGGTCAACAGCAACTCCAATCTCTTGAATTGGAGTTGCTGTTGACCCG |

**Supplementary file 1e The primers used in Real-time PCR (5’-3’).**

| Gene | Forward Primer | Reverse Primer |
| --- | --- | --- |
| *Mus-Mir802-5p* | CGGCGTCAGTAACAAAGATTC | TATGGTTTTGACGACTGTGTGAT |
| *Pri-mus-Mir802* | TCCCCACCTGACTCTACATAACCT | CGTCCTCTCATCTTCCCTTTCGA |
| *Mus-Traf3* | CAGCCTAACCCACCCCTAAAG | TCTTCCACCGTCTTCACAAAC |
| *Mus-Srebp1a* | ATGGACGAGCTGGCCTTCGGTGAGGCGGC | CA  GGAAGGCTTCCAGAGAGGA |
| *Mus-Tnfa* | CCCTCACACTCAGATCATCTTCT | GCTACGACGTGGGCTACAG |
| *Mus-Il6* | TAGTCCTTCCTACCCCAATTTCC | TTGGTCCTTAGCCACTCCTTC |
| *Mus-Il1b* | GCAACTGTTCCTGAACTCAACT | ATCTTTTGGGGTCCGTCAACT |
| *Mus-Inos* | GTTCTCAGCCCAACAATACAAGA | GTGGACGGGTCGATGTCAC |
| *Mus-Ccl2* | TTAAAAACCTGGATCGGAACCAA | GCATTAGCTTCAGATTTACGGGT |
| *Mus-Fizz1* | CCAATCCAGCTAACTATCCCTCC | ACCCAGTAGCAGTCATCCCA |
| *Mus-Chil3* | CAGGTCTGGCAATTCTTCTGAA | GTCTTGCTCATGTGTGTAAGTGA |
| *Mus-Arg1* | CTCCAAGCCAAAGTCCTTAGAG | AGGAGCTGTCATTAGGGACATC |
| *Mus-Il10* | GCTCTTACTGACTGGCATGAG | CGCAGCTCTAGGAGCATGTG |
| *Mus-F4/80* | TGACTCACCTTGTGGTCCTAA | CTTCCCAGAATCCAGTCTTTCC |
| *Mus-Adipor1* | AGACAACGACTACCTGCTACA | GTGGATGCGGAAGATGCTCT |
| *Mus-Adipor2* | GGAGTGTTCGTGGGCTTAGG | GCAGCTCCGGTGATATAGAGG |
| *Mus-Col1a1* | GCTCCTCTTAGGGGCCACT | CCACGTCTCACCATTGGGG |
| *Mus-Col3a1* | CTGTAACATGGAAACTGGGGAAA | CCATAGCTGAACTGAAAACCACC |
| *Mus-Col4a1* | CTGGCACAAAAGGGACGAG | ACGTGGCCGAGAATTTCACC |
| *Mus-Col6a1* | CTGCTGCTACAAGCCTGCT | CCCCATAAGGTTTCAGCCTC |
| *Ccl5* | GCTGCTTTGCCTACCTCTCC | TCGAGTGACAAACACGACTGC |
| *Ccl7* | GCTGCTTTCAGCATCCAAGTG | CCAGGGACACCGACTACTG |
| *Ccl12* | ATTTCCACACTTCTATGCCTCCT | ATCCAGTATGGTCCTGAAGATCA |
| *Ccl19* | GGGGTGCTAATGATGCGGAA | CCTTAGTGTGGTGAACACAACA |
| *Ccl20* | GCCTCTCGTACATACAGACGC | CCAGTTCTGCTTTGGATCAGC |
| *Ccl21a* | GTGATGGAGGGGGTCAGGA | GGGATGGGACAGCCTAAACT |
| *Cxcl2* | CCAACCACCAGGCTACAGG | GCGTCACACTCAAGCTCTG |
| *U6* | CAGCACATATACTAAAATTGGAACG | ACGAATTTGCGTGTCATCC |
| *Gapdh* | AGGTCGGTGTGAACGGATTTG | TGTAGACCATGTAGTTGAGGTCA |
| *Rn18s* | CCCGAAGCGTTTACTTTGA | ACTTTGGTTTCCCGGAAG |
| *Hus-Mir802-5p* | GCCGCGCAGTAACAAAGATT | TATGGTTGTTCACGACTCCTTCAC |
| *Hus-pri-Mir802-5p* | TTCTCTGCAGCCTCTTGTGTCAT | TTCTCCTTGTTGCATGATGGACA |
| *Hus-Traf3* | TCTTGAGGAAAGACCTGCGAG | GCGATCATCGGAACCTGACT |
| Chip R1 | ACACAATCTTCAGTGTGAA | GTAAAATGGTAAACGTGTGT |
| Chip R2 | TATACACTATATAACGAGG | ACGGCCAGCCCCCACCTGT |
| Chip R3 | AAAGCGTGGACAGAGCTGA | CGTCGTGTGCTCCACAGCA |
| Chip R4 | CGACCCCAGCCAAGGCCAG | GTGCTAGATTCGTGTACAA |
| Chip R5 | TAACATATATATAGAGGGG | GCCTGGCTACCCTCTAGCA |
| Chip R6 | GTTGGTGTTTTGTCTGGGA | TGCGGGCTGGCCTGGGGTG |
| Chip R7 | GGAAAGGGGACAAACAGAA | AGCCTTTCTTAGAGGCCCA |
| Chip R8 | ATGCCAAGTGCTGAGCCAT | GGCAAGTCCCCTGCAAGTT |
| Chip R9 | GGGCACAGCTTGCATGCCA | CTGGCGTGAGGCCTAGGTT |
| Chip R10 | AAGCCTGGCCCCGGTTTTC | TGCCGTTGTAATCCCCGTG |
| Chip R11 | AGCTCTTCTTCCTCGGAGT | AGTTACTTTGCCAGTCTAG |
| Chip R12 | CCTGTCCTTCCACAAGAA | TAAGGTAGGTGAGCAGGC |
| Chip R13 | CTGGGTTAGGTAGGGAAT | TAAGGTAGGTGAGCAGGC |
| Chip R14 | TGGGTTAGGTAGGGAATG | CTCTGCATCACCACAGAAA |
| Chip R15 | GAGCAAAGAAAAGTTTCTG | AGGCCAGGAAGGGACATAG |
| Chip R16 | AGAGGTGCAGCCCCTCTG | ATGAGTGTTCCTTCTCCT |
| Chip R17 | TGGCCATCTCCCTCCTAC | GCAGTTGTCAGTCACCCA |
| Chip R18 | GGTCCATGCTCAGAAAGC | GATCATGAGAGCACACAG |
| Chip R19 | TCTATATTGTAGATCAAT | GGGGGATTCATCTAGACCT |
| Chip R20 | AGACAGCAGTAGGAGGTTA | ATGGCTAGGTTTTCTTATG |
